# An inhibitory brainstem pathway reduces visual detection during background motion

**DOI:** 10.1038/s41467-026-72619-x

**Published:** 2026-05-07

**Authors:** Xiao-lin Chou, Milena Russo, Yingtian He, Loridee De Villa, Sabrina Amato, Bao-hua Liu

**Affiliations:** 1 Department of Biology, University of Toronto Mississauga, Mississauga, ON Canada; 2 Department of Cell and Systems Biology, University of Toronto, Toronto, ON Canada

**Keywords:** Motion detection, Sensory processing, Neural circuits, Inhibition

## Abstract

Moving backgrounds profoundly impact object perception, a crucial process for parsing complex visual scenes. This motion-induced modulation has traditionally been attributed to visual cortical circuits. However, recent evidence that brainstem activity is also influenced by background motion raises the intriguing question of whether subcortical circuits play a role in this perceptual phenomenon. Here, we demonstrate that inhibitory projections from mouse nucleus of the optic tract (NOT)—a brainstem structure mediating reflexive behaviors—impair superior colliculus (SC)-dependent visual detection during background motion. Specifically, the inhibitory NOT projections to SC are selectively activated by global, but not local, background motion to suppress SC activity. Remarkably, silencing this NOT-SC pathway relieves the suppression of SC activity in such motional context and alleviates the motion-induced impairments in visual detection. Our findings reveal that motion-sensitive brainstem circuits suppress subcortical processing to shape visual perception, underscoring the underappreciated role of the brainstem in visual cognition.

## Abstract (web summary)

Background motion profoundly impacts visual perception. Here, the authors show that an inhibitory brainstem pathway, recruited by global background motion, suppresses superior colliculus activity and visual perception mediated by this structure.

## Introduction

Visual perception of objects is profoundly influenced by their surroundings^1–16^. This process, known as contextual modulation, allows the brain to efficiently parse complex visual scenes and adapt to dynamic environments^17–21^. A prime example of this phenomenon is the perceptual effects of background motion, which arises when the visual surroundings move relative to the objects. Psychophysical studies have reported that such background motion can potently impair or enhance object saliency^1–10^, depending on differences in visual features (contrast, direction, speed, etc.) between the object and its background. This modulation is essential for fundamental visual operations, from the recognition of object shape, figure-ground segregation, depth perception to perceptual constancy^2,3,17,22–25^. Yet, the circuit mechanisms underlying the perceptual effects of background motion on object saliency remain poorly understood.

For decades, research on the neural substrates underlying motion modulation of visual perception has focused on the visual cortex, given its importance in motion processing^26–28^. Specifically, along the dorsal stream of cortical visual processing, neurons become increasingly responsive to background motion^28–32^. Further, consistent with perceptual effects of background motion, the responses of neurons in various visual cortices to stimuli presented within their receptive fields can be enhanced or suppressed by background motion presented in surrounding regions^33–41^. The polarity and strength of this modulation depend on the relative orientation, motion direction and contrast of the center and surround stimuli and are accounted for by several cortical mechanisms including feedback inputs from higher visual areas and local inhibition^42–49^. These findings, while not causally linking activity changes to perceptual effects, have led to the idea that modulatory effects of background motion on perception primarily arise from the visual cortex^1–6^. However, several recent studies imply a potential contribution of phylogenetically older subcortical structures to these modulatory effects. As in the visual cortex, visual activities in subcortical structures, like the dorsal lateral geniculate nucleus (dLGN) and superior colliculus (SC), are also prominently enhanced or suppressed by background motion^50–57^. Notably, this subcortical motion modulation appears to be computed within subcortical circuits, because it does not require visual cortical activity^53,58–60^, and motion modulation of visual activity also exists in various non-mammalian species, which lack a neocortex^61–66^. Furthermore, the perturbation of subcortical circuits compromises the suppression of visual cortical activity by background motion^67,68^, suggesting that even cortical motion modulation may partly rely on subcortical circuits. Nevertheless, the circuits underlying subcortical motion modulation and their precise roles in visual perception remain unknown.

Among subcortical visual nuclei, the nucleus of the optic tract (NOT) in the brainstem is an appealing candidate, as this primitive structure preferentially responds to global optic flow to drive reflexive eye movements^69–71^, making it well-suited to encode background motion^72–75^. Such response property raises the intriguing possibility that the NOT may also contribute to the modulatory effects of background motion. To test this hypothesis, we developed a visually guided behavioral task in mice to assess whether background motion impairs or enhances subcortically driven visual perception. Using this paradigm, we aimed to define the contribution of NOT to this perceptual effect and to pinpoint the underlying neural circuits. Potential findings would broaden our understanding of how ancient brainstem circuits participate in the cognitive processes of visual perception.

## Results

### Background motion impairs detection of luminance changes by disrupting sSC activity

**Fig. 1:**
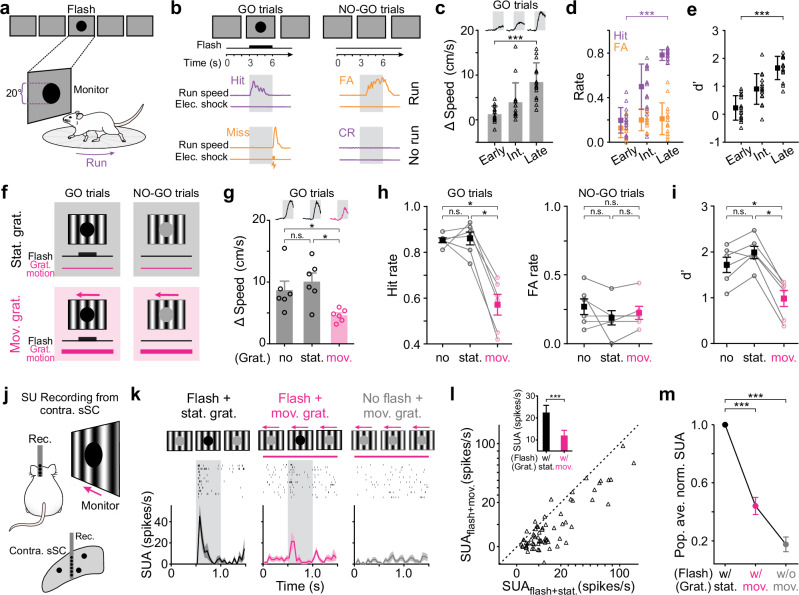
Background motion impairs detection of luminance changes by disrupting sSC activity. **a**–**e** Mouse behavioral paradigm of detecting local luminance changes (flashes). **a** Schematic of behavioral setup. A mouse is trained to run on a treadmill when the luminance of a circular region (20°) in a uniform background decreases (black flash). **b** Behavioral contingencies. Top, schematic of visual stimulation in GO (black flash) or NO-GO trials (blank). Bottom, example traces of running speed and electric (Elec.) shock in hit, false alarm (FA), miss, and correct rejection (CR) trials. Shades, timing of flashes or blank stimuli. **c**–**e** Increment of mean running speeds during black flashes (**c**), hit/false alarm rates (**d**) and d prime (d’, **e**) in early (first two), intermediate (Int., middle two) and late (last two) training sessions (*n* = 15 mice). Top in **c** average speed traces in GO trials of an example animal in the 3 training sessions. Shade width=3 sec (timing of flashes); shade height=12.1 cm/s. One-sided Wilcoxon signed-rank test, *p* = 3.1E-05 for **c**, for hit rates in **d** and for **e**. Two-sided Wilcoxon signed-rank test, *p* = 0.12 for false alarm rates in **d**. **f**–**i** Impact of background motion on flash detection. **f** Schematic of visual stimulation. Note that a static (Stat.) or moving (Mov.) grating (grat.) surrounds the circular region where black flashes appear. **g**–**i** Increment of mean running speed during flashes (**g**), hit/false alarm rates (**h**) and d’ (**i**) in trials with static- or moving-grating backgrounds, compared to those with a uniform background in last training session (*n* = 6 mice). Top in **g** is presented as top in **c**. Shade width=3 sec; shade height=25.1 cm/s. One-sided Wilcoxon signed-rank test; *p* = 0.031, no versus moving gratings in **g**; *p* = 0.016, static versus moving gratings in **g**, no/static versus moving gratings for hit rate in **h**, and no/static versus moving gratings in **i**. Two-sided Wilcoxon signed-rank test; *p* = 0.56, no versus static gratings in **g**; *p* = 0.84, no versus static gratings for hit rate in **h**; *p* = 0.63, no versus moving gratings for false alarm rates in **h**; *p* = 0.25, no/moving versus static gratings for false alarm rates in **h**; *p* = 0.063 for no versus static gratings in **i**. No multiple-comparison adjustments are made for **g**–**i**. **j**–**m** Impact of background motion on black flash-evoked activity in the superficial superior colliculus (sSC). **j** Schematic of single-unit (SU) recording. Pink arrow, grating motion. **k** Activity of an example sSC neuron evoked by flashes with static gratings (left), flashes with moving gratings (middle) and moving gratings only (right). Note that a moving grating surrounding the center flashes does not activate the sSC neuron. Top, schematic of visual stimulation. Pink arrow, grating motion. Bottom, raster plots and peristimulus time histograms (PSTHs) of spikes. Pink bars, timing of moving gratings. Shades, timing of flashes. **l** Comparison of single-unit activity (SUA) evoked by black flashes between moving- and static-grating trials. Dashed line, unity line. Inset, population-averaged SUA (*n* = 67 neurons, one-sided Wilcoxon signed-rank test, *p* = 1.8E-11). **m** Population-averaged normalized SUA evoked by the three stimuli (*n* = 67 neurons, one-sided Wilcoxon signed-rank test, *p* = 3.4E-11 for flashes with static versus flashes with moving gratings, *p* = 8.6E-13 for flashes with static gratings versus moving gratings only). *p < 0.05; ***p < 0.001. n.s., not significant. Traces and summary data are shown as mean ± s.e.m.

We first developed a visually guided behavioral task to assess subcortically-mediated visual perception. Given the sensitivity of subcortical nuclei to local luminance changes^76–81^, the task used a target stimulus consisting of a 20° black circular patch briefly appearing on a gray background (that is, a local black flash; see Methods, Fig. 1a). A head-fixed mouse was trained to run when the local black flash was presented to the animal’s right eye (Fig. 1a, b Hit in GO trials, Supplementary Fig. 1a-b). If the animal did not react to the flash, it was penalized with an electrical tail shock (see Methods, Fig. 1b Miss in GO trials, Supplementary Fig. 1a-b). To estimate false alarm and correct rejection rates, in 20% of randomized trials no flash was presented (blank stimulus, Fig. 1b NO-GO trials). Mice learned this task quickly, showing a dramatic increase in the increment of running speed during flashes, hit rate, and d prime (d’), with little change in the false-alarm rate (Fig. 1c–e, Supplementary Fig. 1c-h).

To examine whether background motion affects flash detection in successfully trained (hereafter, expert) mice, we added a sinusoidal grating surrounding the area where the flash appeared (hereafter referred to as the background), and made the grating move temporo-nasally in reference to animal’s right eye in half of trials (see Methods, Fig. 1f, Supplementary Videos 1–4). Since moving gratings can elicit eye movements^69,82^ that may interfere with flash detection, we selected grating parameters that would not induce eye movements (SF, 0.08 cpd; TF, 2 Hz; size, 90°×60° with a 20°-diameter gray patch; Supplementary Fig. 1i,j). Intriguingly, while static gratings had no effect on animals’ task performance, moving gratings substantially impaired their performance (Fig. 1g–i). Specifically, the increment of running speed was reduced by 54% (Δspeed from 10.1 ± 1.4 to 4.6 ± 0.4 cm/s); hit rate dropped from 0.86 ± 0.03 to 0.57 ± 0.05 but the false alarm rate did not change, leading to a 51% reduction in d’ (Z-score of Hit rate - Z-score of FA rate, from 1.99 ± 0.13 to 0.98 ± 0.17; Fig. 1g–i, Supplementary Movies 1–4). The reduced increment of running speed was primarily accounted for by the reduced hit rate, but not locomotor capability, since locomotory parameters in hit trials were unaffected by background motion (Supplementary Fig. 1k,l). To examine whether the impairment in task performance depends on the strength of flash stimuli, we systematically varied the luminance change, namely the contrast, of the central circular patch. We found that when the contrast decreased, the hit rate dropped dramatically. Notably, the background motion impaired the task performance in a contrast-dependent manner, producing the largest reduction in hit rate at 100% contrast (Supplementary Fig. 1m). Overall, these results indicate that background motion disrupts visual perception of local luminance changes, particularly at high contrast. These findings are reminiscent of previous reports that background motion impairs object saliency in human subjects^6–9^.

Next, we sought to understand what underpins the perceptual impairment by background motion. The SC is a strong candidate due to its essential role in the perception of various visual features^76,83–87^ and its sensitivity to local flashes^77,81^. To qualify as a candidate, SC should meet two criteria: (1) its activity must be necessary for animals to perform the visual detection task; (2) its activity should be modulated by background motion. To examine the first criterion, we silenced the SC of an expert mouse by optogenetically activating its inhibitory SC neurons with channelrhodopsin-2 (ChR2) in half of the behavioral trials (Supplementary Fig. 2a). SC silencing severely compromised animals’ task performance, shown by the decreased increment of running speed during flashes, hit rate and d’ (Δ speed: 11.2 ± 3.5 to 3.5 ± 0.8 cm/s, hit rate: from 0.80 ± 0.02 to 0.38 ± 0.06, d’: from 2.08 ± 0.09 to 0.81 ± 0.15), but did not change the locomotor program of the behavior (Supplementary Fig. 2b–h). These results indicate that SC activity is indeed required for detection of flashes, which is consistent with a recent report on the importance of mouse SC in visual perception of luminance changes^76^.

To evaluate the second criterion, we performed single-unit recordings from the superficial SC (sSC)—the visual layer of SC—in awake head-fixed mice while presenting local flashes on a static or moving grating background to their contralateral eye (see Methods, Fig. 1j, Supplementary Fig. 3a). Receptive field locations of sSC neurons were estimated according to multi-unit activity evoked by square flashes presented one at a time within a 6 × 8 grid (see Methods). In agreement with previous reports^77,81^, most (76.1%) sSC neurons exhibited strong responses to black flashes (OFF) and/or white flashes (increase of local luminance, ON) presented within their receptive fields (Fig. 1k left, Supplementary Fig. 3a,b left), characterized by a short onset latency (49.1 ± 14.5 ms; Supplementary Fig. 3c). The sSC activity showed a clear preference for black flashes (FR: 7.28 ± 1.13 vs 21.27 ± 3.08 spikes/s, percentage of neurons: 51.1% vs 69.8%, ON response vs OFF response, Supplementary Fig. 3d). Remarkably, when background gratings drifted, the vast majority (89%) of these responsive sSC neurons exhibited a substantial reduction in activities evoked by either black or white flashes (Fig. 1k–m, Supplementary Fig. 3b,e,f), despite the gratings alone eliciting minimal responses (Fig. 1k right,m, Supplementary Fig. 3b right,f). On average, background motion suppressed flash-evoked sSC activity by about 60% (Fig. 1m, Supplementary Fig. 3f), with noticeable variation across individual cells (Supplementary Fig. 3g). This suppression could not be attributed to either eye movements or locomotion (Supplementary Fig. 4). Together, these results indicate that background motion impairs the detectability of local luminance changes, at least partly by disrupting visual processing in the sSC. Thus, subcortical structures contribute to the suppressive effects of background motion on visual perception.

### Inhibitory NOT neurons provide functional inhibition to sSC neurons

**Fig. 2:**
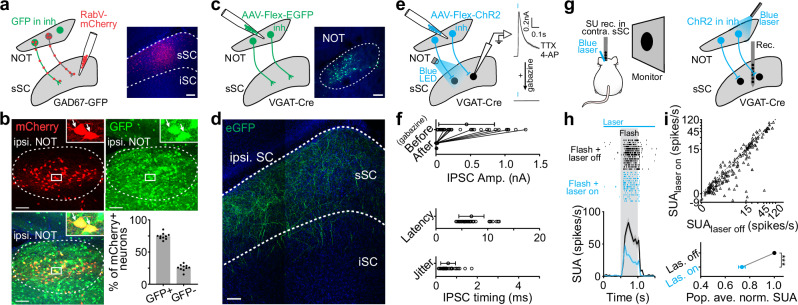
NOT provides functional inhibition to sSC neurons. **a**, **b** RabV-mCherry virus injected into the sSC retrogradely transfects inhibitory (inh.) NOT neurons preferentially, compared to excitatory NOT neurons. Blue, DAPI. **a** Left, schematic of experimental design. Right, coronal slice containing the injection site in sSC. sSC, superficial layer of SC; iSC, intermediate layer of SC. **b** Coronal slice of the NOT and the quantification of sSC-projecting (mCherry + ) inhibitory (GFP + ) NOT neurons. Inset, higher magnification of the enclosed area. White arrows, example sSC-projecting inhibitory NOT neurons. *n* = 10 slices from 3 mice. **c**, **d** Inhibitory NOT neurons project to the sSC preferentially, compared to iSC (*n* = 3 mice). Blue, DAPI. **c** Left, schematic of experimental design. Right, coronal slice containing the injection site in NOT. **d** Coronal slice of the SC (3 mice). **e**, **f** GABA_A_ receptor-mediated IPSCs in sSC neurons evoked by optogenetic stimulation of inhibitory NOT-sSC projections on slice preparations. **e** Left, schematic of experimental setup. Right, IPSCs in an example sSC neuron before and after the bath application of gabazine. Note that poly-synaptic components are blocked by tetrodotoxin (TTX) and 4-aminopyridine (4-AP) in the bath. Blue bar, timing of LED illumination. **f** Summary of the amplitude (Amp., before and after gabazine application, top), latency (middle) and jitter (bottom) of light-evoked IPSCs (*n* = 34 neurons). **g**–**i** Optogenetic activation of inhibitory NOT neurons suppresses flash-evoked sSC activity. **g** Schematic of experimental setup. **h** Example sSC neuron. Raster plots (top) and peristimulus time histograms (PSTH, bottom) of spiking responses to black flashes, in activation (Las. on) or control (Las. off) trials. Shade, timing of flashes. Blue bar, timing of laser. **i** Comparison of single-unit activity (SUA, top) and population-averaged normalized SUA (bottom) in response to local flashes, between activation (Las. on) and control trials (Las. off, *n* = 183 neurons, one-sided Wilcoxon signed-rank test, *p* = 4.6E-12 for SUA, *p* = 2.1E-16 for normalized SUA). Dashed line, unity line. Scale bars in **a**–**d** 100 µm. Summary data in **b** and **i** shown as mean±s.e.m. Traces in **e** and summary data in **f** shown as mean±s.d. ***p < 0.001.

To investigate the subcortical mechanisms underlying the perceptual impairment by background motion, we searched for potential sources of inhibitory signals transmitted to the sSC during background motion. First, to identify the inhibitory projections to the sSC, we injected retrograde rabies viruses encoding mCherry into the sSC of GAD67-GFP mice unilaterally (Fig. 2a). Among the structures with retrogradely labeled neurons (Fig. 2b, and Supplementary Fig. 5a), the NOT emerged as a prime candidate for the source of motion-associated inhibitory signals, given its strong responses to large moving gratings^72–75^, and the predominantly inhibitory (74%) nature of NOT-sSC projections (Fig. 2b)^88,89^. Next, we examined the projection targets of inhibitory NOT neurons by conditionally expressing GFP in these inhibitory neurons of Vgat-Cre mice (Fig. 2c). We observed dense fluorescent axons in ipsilateral sSC, but very sparse ones in the intermediate layer of ipsilateral SC (ipsilateral iSC, Fig. 2d). Similar layer-specific axons were present in contralateral SC, albeit at a much lower density (Supplementary Fig. 5b). Thus, inhibitory NOT neurons preferentially target the ipsilateral sSC. Interestingly, we also found fluorescent axons in visual perception-related structures other than sSC, such as the dLGN, ventral lateral geniculate nucleus (vLGN) and lateral posterior thalamic nucleus (LP), but not in oculomotor or locomotor structures (Supplementary Fig. 5b,c)^76,85,90–92^. This innervation pattern suggests that inhibitory projections from the NOT to these visual nuclei form a specific circuit module involved in visual perception rather than in motor control.

To determine whether inhibitory NOT-sSC projections innervate neurons in the sSC, we conditionally expressed ChR2 in inhibitory NOT neurons of Vgat-Cre mice and performed whole-cell recordings from sSC neurons in acute slices from these mice (Fig. 2e left). Photo-stimulation of ChR2-expressing NOT axons in the sSC elicited GABA_A_ receptor-mediated inhibitory postsynaptic currents (IPSCs, 443 ± 402pA, mean±s.d.) in the majority of sSC neurons (34 out of 51), characterized by short latency (6.9 ± 2.4 ms, mean±s.d.) and minimal jitter (0.59 ± 0.36 ms, mean±s.d., Fig. 2e, f). Finally, to assess whether inhibitory NOT-sSC projections can suppress sSC activity, we conducted in vivo single-unit recordings to monitor flash-evoked sSC activity while optogenetically activating inhibitory NOT neurons in half of the trials (see Methods, Fig. 2g). Given the sSC’s preference for black flashes^77^ (Supplementary Fig. 3d), we used these stimuli in subsequent experiments. Optogenetic activation resulted in a 26% decrease in flash-evoked firing rates of sSC neurons (Fig. 2h, i), which could not be attributed to eye movements, locomotion, or blue light illumination alone (Supplementary Fig. 6). Overall, these results demonstrate that inhibitory NOT neurons provide direct inhibition to sSC neurons, which is sufficient to downregulate visual processing in the sSC.

### Inhibitory NOT-sSC projections prefer global temporo-nasal background motion

**Fig. 3:**
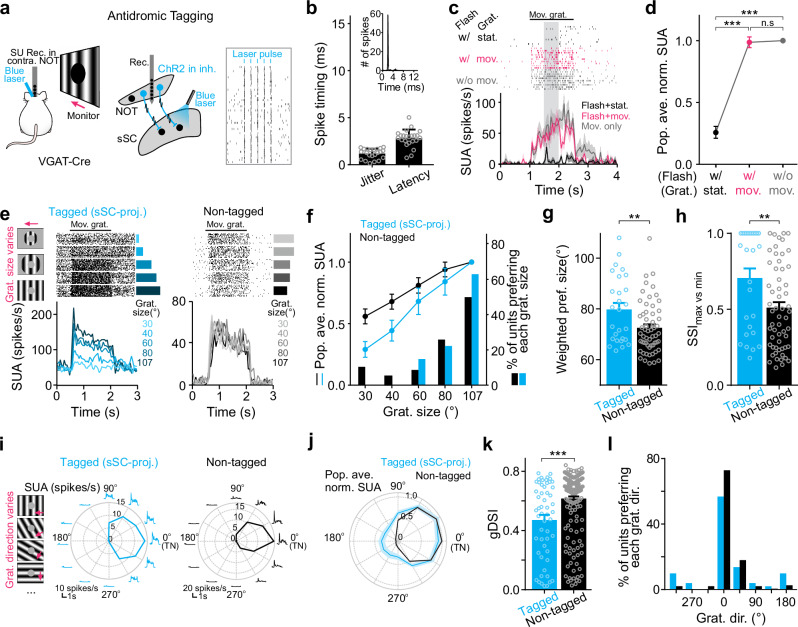
Inhibitory NOT-sSC projections prefer global temporo-nasal background motion. **a** Antidromic tagging of sSC-projecting inhibitory (inh.) NOT neurons. Left & middle, schematic of experimental setup. Note that single-unit recording is carried out in contralateral NOT. Pink arrow, grating motion. Right, raster plot of antidromic spikes from an example neuron. Blue bars, timing of laser pulses. **b** Summary of jitter and latency of antidromic spikes (*n* = 23 neurons). Inset, histogram of spike timing in reference to laser pulses from the neuron in **a**. Data shown as mean ± s.d. **c** Raster plot (top) and peristimulus time histogram (PSTH, bottom) of spiking responses of an example sSC-projecting inhibitory NOT neuron to flashes with static gratings (stat. grat.), flashes with moving gratings (mov. grat.) and moving gratings only. w/, with black flashes; w/o, without black flashes. Shade, timing of flashes. Bar, timing of moving gratings. **d** Population-averaged normalized single-unit activity (SUA, *n* = 23 neurons) of sSC-projecting inhibitory NOT neurons evoked by the three visual stimuli in **c**. One-sided Wilcoxon signed-rank test, *p* = 1.4E-05, flashes with static gratings versus flashes/no flash with moving gratings. Two-sided Wilcoxon signed-rank test, *p* = 0.89, flashes versus no flash with moving gratings. No multiple-comparison adjustments are made. **e**–**h** Comparison of size tuning between tagged (sSC-projecting inhibitory, *n* = 27 neurons) and non-tagged NOT neurons (*n* = 58 neurons). **e** Firing of example neurons evoked by moving gratings of various sizes. Top, raster plots of spikes (left) and bar plots of SUA normalized to the firing rate at preferred size (right). Bottom, PSTHs. Bar, timing of moving gratings. **f** Population-averaged normalized size tuning curves of NOT neurons (lines, 2-way ANOVA, *p* = 0.0008) and histograms of their preferred grating sizes (bars, one-sided Fisher’s exact test, *p* = 0.03). **g** Weighted preferred size of NOT neurons. **h** Size selectivity index_max vs min_ of NOT neurons. One-sided Wilcoxon signed-rank test, *p* = 0.0078 for **g**, *p* = 0.0015 for **h**. **i**–**l** Comparison of direction tuning between tagged (sSC-projecting inhibitory) or non-tagged NOT neurons (*n* = 54 vs 197 neurons for **j** and **k**; 51 vs 195 neurons for **l**). **i** Direction tuning curves of example neurons. Surrounding traces, PSTHs of spikes evoked by corresponding motion directions. TN, temporo-nasal. **j**, Population-averaged normalized direction tuning curves. **k** Global direction selectivity index (gDSI). One-sided Wilcoxon signed-rank test, *p* = 1.8E-05. **l** Histograms of preferred directions determined by vector summation (one-sided Fisher’s exact test, *p* = 0.0005). **p < 0.01; ***p < 0.001. n.s., not significant. Data in **c**,**d**,**f**,**h**,**j**,**k** shown as mean ± s.e.m.

Response properties of neurons are supportive of their functional roles in neural circuits. Do sSC-projecting inhibitory NOT neurons possess response properties that enable them to suppress sSC activity during background motion? To address this, we compared the activity of this NOT population in response to local black flashes and/or surrounding moving gratings, as previously described (Fig. 1k schematic). To identify sSC-projecting inhibitory NOT neurons in single-unit recordings, we employed optogenetic antidromic tagging, where photo-stimulation of ChR2-expressing inhibitory NOT axons in the sSC elicited antidromic spikes with minimal jitter (1.2 ± 0.5 ms, mean±s.d.) and short latency (2.8 ± 0.9 ms, mean±s.d., Fig. 3a, b). We found that these inhibitory projection neurons exhibited an opposite preference for visual features compared with flash-sensitive sSC neurons (Fig. 1k-m): they responded weakly to central black flashes (SUA = 6.4 ± 1.5 spikes/s), but robustly to surrounding moving gratings regardless of the presence of flashes (SUA = 24.7 ± 3.8 vs 25.3 ± 4.0 spikes/s, with vs without flashes, Fig. 3c, d). This indicates that sSC-projecting inhibitory NOT neurons are sensitive to background motion, but not to local luminance changes. Moreover, these neurons responded to moving background gratings simultaneously with sSC activation by flashes (Supplementary Fig. 7a), suggesting that this projection can suppress early sSC activity.

We then tested whether sSC-projecting inhibitory NOT neurons specialize in encoding global, rather than local, background motion by varying the size of moving background gratings (Fig. 3e). Most sSC-projecting inhibitory neurons (tagged) preferred large gratings over small ones (85% prefer 80/107° vs 0% prefer 30/40°; weighted pref. size, 79.8 ± 2.5°; Fig. 3e–g), and had monotonically increasing size-tuning curves (Fig. 3e left,f, Supplementary Fig. 7b,c), making them distinct from visual cortical or SC neurons^44,51,93,94^. Moreover, tagged neurons showed strong selectivity for grating size (SSI_max vs min_: 0.71 ± 0.06, slope: 0.009 ± 0.001, Fig. 3h, Supplementary Fig. 7b–d). In contrast, NOT neurons not antidromically stimulated (non-tagged) had more diverse size-tuning curves, with some even showing monotonically decreasing responses with increasing size (Fig. 3e right, Supplementary Fig. 7c); these neurons were relatively biased toward smaller gratings (Fig. 3f, g) and had weaker size selectivity (SSI_max vs min_: 0.51 ± 0.04, slope: 0.006 ± 0.001, Fig. 3h, Supplementary Fig. 7c,d). These results indicate that sSC-projecting inhibitory NOT neurons constitute a functionally distinct subpopulation that preferentially respond to large moving gratings, thereby conveying signals encoding global background motion to the sSC.

In addition to its sensitivity to global motion, another unique property of the NOT is its bias toward temporo-nasal motion (relative to the contralateral eye)^70,73,82,95^. We next investigated whether sSC-projecting inhibitory NOT neurons share this property by systematically varying the direction of moving background gratings. Tagged NOT neurons exhibited obvious direction selectivity, albeit weaker than that of non-tagged neurons (gDSI: 0.47 ± 0.03 vs 0.62 ± 0.01; tagged vs non-tagged, Fig. 3i–k, Supplementary Fig. 7e–g). Among the direction selective neurons (gDSI>0.05), the majority (70.6%) of tagged NOT neurons preferred gratings moving temporo-nasally, similar to the non-tagged population (0° and 45°, Fig. 3l). Taken together, sSC-projecting inhibitory NOT neurons are tuned to global temporo-nasal background motion, making this population well-suited to mediate the impairment of sSC functions by background motion (Fig. 1).

### sSC activity is preferentially suppressed by global temporo-nasal background motion

**Fig. 4:**
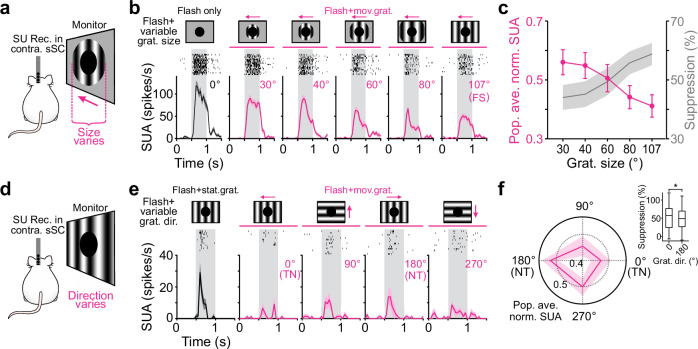
sSC activity is preferentially suppressed by global temporo-nasal background motion. **a**–**c** Size-dependence of the suppressive effect of background motion on flash-evoked sSC activity. **a** Schematic of experimental setup. Note that single-unit recording is carried out in contralateral sSC. Pink arrow, grating motion. **b** Single-unit activity (SUA) of an example sSC neuron to black flashes surrounded by moving gratings (mov. grat.) of various sizes. Top, schematic of visual stimuli. Bottom, raster plots and peristimulus time histograms (PSTHs) of spiking responses. Pink bars, timing of moving gratings. Shades, timing of flashes. Grat., grating; FS, full screen. **c** Population-averaged normalized size tuning curves of flash-evoked SUA (pink) and motion-induced suppression of flash-evoked SUA (gray, *n* = 76 neurons, one-sided linear regression analysis, *p* = 0.002). **d**–**f** Direction-dependence of the suppressive effect of background motion on flash-evoked sSC activity. **d** Schematic of experimental setup. **e** Single-unit activity (SUA) of an example sSC neuron to black flashes surrounded by moving gratings (mov. grat.) of various directions. Data presented as in **b**. **f** Population-averaged normalized direction tuning curve of flash-evoked SUA (*n* = 82 neurons, one-sided Wilcoxon signed-rank test, *p* = 0.042). TN, temporo-nasal. NT, naso-temporal. Inset, Tukey box-whisker plot of suppression of flash-evoked SUA by TN (0°) and NT (180°) directions; center line, box edges and whiskers represent median, 25% and 75% quantile and minimum and maximum values within 1.5 times interquartile range from the quartile. *p < 0.05. Traces and summary data shown as mean±s.e.m.

Given the preference of sSC-projecting inhibitory NOT neurons for large gratings moving in the temporo-nasal direction (Fig. 3), we wondered whether the motion-induced suppression of sSC activity also depends on the size and direction of moving background gratings. To explore this, we first examined how the size of surrounding moving gratings affects the motion-induced suppression of sSC activity (Fig. 4a–c). A small moving grating of 30° in diameter suppressed flash-evoked sSC activity by 44.0% (Fig. 4b, c). As we gradually increased the grating size, the suppression of sSC activity increased monotonically, with full-field gratings (107°) producing a suppression 34.1% stronger than that induced by 30° gratings (Fig. 4b,c). This result indicates that the suppressive effect on sSC activity favors global motion over local motion, consistent with the size preference of sSC-projecting inhibitory NOT neurons (Fig. 3e–g). Next, we investigated whether the motion-induced suppression of sSC activity depends on the direction of moving gratings (Fig. 4d–f). While naso-temporally moving background gratings (180°) suppressed flash-evoked sSC activity by 47.7%, temporo-nasally moving background gratings (0°) resulted in a suppression 11.0% stronger (Fig. 4e, f). This result indicates that the suppressive effect on sSC activity is biased toward temporo-nasal motion, consistent with the direction preference of sSC-projecting inhibitory NOT neurons (Fig. 3i–l). Overall, our findings further support the idea that inhibitory NOT-sSC projections may mediate the motion-induced suppression of sSC activity.

### Inhibitory NOT-sSC projections suppress sSC activity specifically during global temporo-nasal background motion

**Fig. 5:**
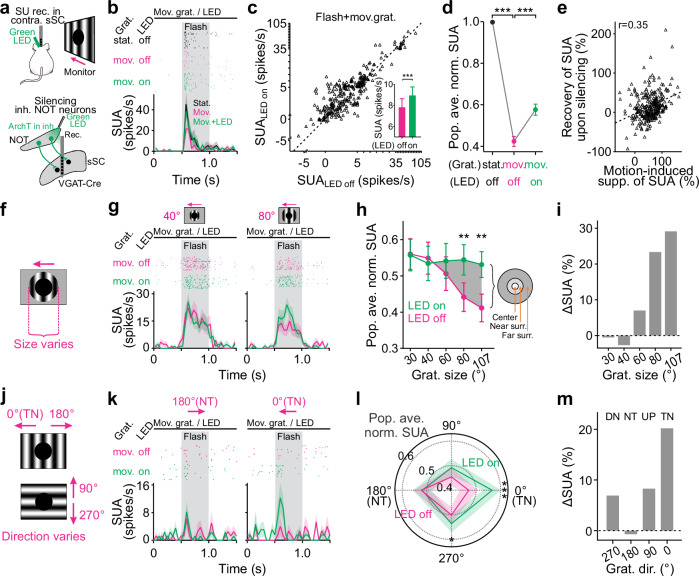
Inhibitory NOT-sSC projections suppress sSC activity specifically during global temporo-nasal background motion. **a** Schematic of experimental setup. Pink arrow, grating motion. Note that inhibitory (inh.) NOT neurons are optogenetically silenced by inhibitory opsin ArchT, while single-unit recording is carried out in contralateral sSC. **b**–**e** Contribution of inhibitory NOT neurons to the suppression of sSC activity by background motion (*n* = 283 neurons). **b** Raster plots (top) and peristimulus time histograms (PSTHs, bottom) of flash-evoked spiking responses of an example sSC neuron under three visual/optogenetic conditions. Shade, timing of black flashes. Bar, timing of moving gratings and LED. Grat., grating; stat., static; mov., moving. **c** Comparison of single-unit activity (SUA) in response to flashes with moving gratings between silencing (LED on) and control conditions (LED off). Dashed line, unity line. Inset, population-averaged normalized SUA. One-sided Wilcoxon signed-rank test, *p* = 3.4E-07. **d** Population-averaged normalized flash-evoked SUA under three visual/optogenetic conditions. One-sided Wilcoxon signed-rank test, *p* = 5.6E-45 for static versus moving gratings with LED off, *p* = 8.8E-11 for LED off versus LED on with moving gratings. **e** Correlation between the recovery in sSC activity upon silencing inhibitory NOT neurons and the suppression of sSC activity exerted by background motion. Dashed line, best-fit line of linear regression. r, correlation coefficient of the linear regression (One-sided linear regression analysis, *p* = 4.6E-10). **f**–**i** Size-dependent contribution of inhibitory NOT-sSC projections to the motion-induced suppression of sSC activity. **f** Schematic of visual stimulation. **g** SUA of an example sSC neuron to black flashes surrounded by small (left, 40°) or large (right, 80°) moving gratings, under silencing (LED on, green) or control conditions (LED off, pink). Top, schematic of visual stimulation. Bottom, raster plots and PSTHs of spikes. **h** Population-averaged size tuning curves of normalized SUA, under silencing (LED on) or control conditions (LED off, *n* = 76 neurons, one-sided Wilcoxon signed-rank test, *p* = 0.0018 for size 80°, *p* = 0.0014 for size 107°). Shade, the suppression exerted by inhibitory NOT-sSC projections. Right, schematic showing that these projections primarily mediate the suppression applied by background motion in the far surround of the center flash. Near surr., near surround (20°–40°). Far surr., far surround ( > 40°). **i** Size-dependence of percentage change in population-averaged normalized SUA upon silencing inhibitory NOT neurons. **j**–**m** Direction-dependent contribution of inhibitory NOT-sSC projections to the motion-induced suppression of sSC activity. Data presented as in **f**–**i** (*n* = 82 neuro*n*s, one-sided Wilcoxon signed-rank test in **l** *p* = 0.0002 for direction 0°, *p* = 0.03 for direction 270°). TN, temporo-nasal. NT, naso-temporal. UP, upwards. DN, downwards. *p < 0.05; **p < 0.01; ***p < 0.001. Traces and summary data shown as mean ± s.e.m.

To evaluate the role of inhibitory NOT-sSC projections in the suppression of sSC activity by background motion, we used archaerhodopsin (ArchT) to optogenetically silence inhibitory NOT neurons (Fig. 5a). Green light illumination induced robust hyperpolarization in ArchT-expressing inhibitory NOT neurons (−22.1 ± 14.8 mV, mean ± s.d., Supplementary Fig. 8a–c), effectively suppressing their spiking activities in response to current injections (Supplementary Fig. 8d). Remarkably, silencing these neurons shifted the distribution of flash-evoked sSC activity rightwards (Supplementary Fig. 8e,f) and increased its strength by 36% (norm. SUA: from 0.42 ± 0.02 to 0.58 ± 0.03; Fig. 5b–d), alleviating a considerable portion of the motion-induced suppression of sSC activity (26%, Fig. 5d). Notably, the amount of sSC activity recovered upon silencing was positively correlated with the amount suppressed by the moving gratings (Fig. 5e). This correlation indicates that the cell-to-cell variation in motion-induced suppression of sSC activity (Supplementary Fig. 3g) can be partly explained by the differential contribution of inhibitory NOT neurons. Additionally, this recovery could not be attributed to eye movements, locomotion, or green light illumination alone (Supplementary Fig. 9). Together, these findings demonstrate that inhibitory NOT neurons play a substantial role in the suppression of flash-evoked sSC activity exerted by background motion.

Since inhibitory NOT neurons project not only to the sSC, but also to other nuclei (Supplementary Fig. 5a,b)^50,96^, background motion may suppress sSC activity via direct NOT-sSC projections and/or via polysynaptic relays through these nuclei. To assess the contribution of direct projections to the motion modulation of sSC activity, we used a targeting-enhanced mosquito homolog of vertebrate encephalopsin (eOPN3), which can effectively disrupt synaptic transmission (Supplementary Fig. 10a–d). Using this tool, we selectively silenced the direct inhibitory NOT-sSC projections by shining green light on eOPN3-expressing inhibitory NOT axons within the sSC (Supplementary Fig. 10e). This projection-specific silencing recapitulated the effects of silencing inhibitory NOT neurons with ArchT (Fig. 5a–e). First, it significantly enhanced sSC activity evoked by local flashes in the presence of background motion (firing rate: 13.6 ± 1.9 spikes/s to 15.9 ± 2.1 spikes/s), thereby mitigating the motion-exerted suppression of sSC activity by 32% (Supplementary Fig. 10f–j). Second, it recovered sSC activity to an extent that positively correlated with the degree of suppression caused by background motion (Supplementary Fig. 10k), suggesting that inhibitory NOT-sSC projections contribute to the neuronal variation observed in motion-induced suppression of sSC activity (Supplementary Fig. 3g). Further, the mitigation of motion-induced suppression upon projection-specific silencing matched that upon general silencing of inhibitory NOT neurons (norm. SUA_mov./LEDon_ − norm. SUA_mov./LEDoff_: 0.15 ± 0.02 vs 0.15 ± 0.03, ArchT vs eOPN3; Fig. 5d, Supplementary Fig. 10h). Thus, inhibitory NOT neurons utilize their direct projections to the sSC to suppress the activities of sSC neurons when background motion is present.

Next, since both inhibitory NOT-sSC projections and the motion-induced suppression of sSC activity prefer large background gratings, we hypothesized that the stronger suppression of sSC activity by larger background gratings may arise from these inhibitory projections. To test this hypothesis, we examined the contribution of inhibitory NOT-sSC projections to the motion-induced suppression of sSC activity across various grating sizes. Using the ArchT method, we silenced inhibitory NOT neurons while recording sSC activity and systematically varying grating size (Fig. 5f, g). For small gratings (30° and 40°), silencing these neurons did not affect flash-evoked sSC activity (Fig. 5g left,h,i, Supplementary Fig. 11a). However, as grating size increased to 80° or full screen (107°), optogenetic silencing significantly enhanced flash-evoked sSC activity (norm. SUA: from 0.44 ± 0.04 to 0.54 ± 0.04 for 80°; from 0.41 ± 0.04 to 0.53 ± 0.04 for 107°; Fig. 5g right,h,i, Supplementary Fig. 11a), recapitulating the recovery of suppressed sSC activity observed previously (Fig. 5d). These results indicate that inhibitory NOT-sSC projections contribute to the suppression of sSC activity applied by large, but not small, gratings, consistent with the size preference of their responses (Fig. 3e–g). Notably, under silencing conditions, the suppression of flash-evoked sSC activity no longer increased with grating size (Fig. 5h green vs pink). This loss of size dependence suggests that in sSC neurons inhibition evoked by background motion comes from two concentric regions surrounding the central flashes—near surround (inner grating region within 40° diameter) and far surround (outer grating region beyond 40°)—as visual cortical neurons do^97,98^. In addition, our result identifies the neuronal basis of the far surround: its suppressive effect is mediated by inhibitory NOT-sSC projections, while the suppressive effect from the near surround likely originates from other circuits (Fig. 5h schematic). In summary, these findings underscore the exquisite functional specificity of inhibitory NOT-sSC projections in suppressing sSC activity in the presence of global, but not local, background motion.

Furthermore, we tested the hypothesis that the stronger suppression by temporo-nasal motion may arise from the temporo-nasal bias of inhibitory NOT-sSC projections, by silencing inhibitory NOT neurons when background gratings moved in four cardinal directions (temporo-nasal, naso-temporal, upward, downward, Fig. 5j). Consistent with our hypothesis, optogenetic silencing enhanced flash-evoked sSC activity most prominently when the gratings moved temporo-nasally (0°, from 0.47 ± 0.04 to 0.57 ± 0.04, Fig. 5k–m, Supplementary Fig. 11b). These results indicate that this pathway suppresses sSC activity most effectively in the presence of temporo-nasal motion compared with other directions. It is worth noting that under silencing conditions, some remaining suppression of sSC activity still existed for all tested motion directions, without a temporo-nasal bias (Fig. 5l green). This remaining suppression likely comes from circuits other than the inhibitory NOT-sSC projections. Overall, the contribution of inhibitory NOT-sSC projections to the motion-induced suppression of sSC activity (Fig. 5) aligns with the size and direction bias of their responses (Fig. 3), further indicating that this pathway is a key mediator of the modulatory effects of background motion. Thus, these findings reveal a fundamental mechanism by which the NOT suppresses sSC activity: its inhibitory projections convey unique signals encoding global temporo-nasal motion to the sSC, dampening its visual processing.

The receptive field size of sSC neurons varies widely, ranging from a few degrees to tens of degrees in diameter^77,81,94^. For sSC neurons with receptive fields smaller than 20°, the background gratings were confined to regions outside their receptive fields. In contrast, for sSC neurons with receptive fields larger than 20°, the background gratings extended into and partially overlapped their receptive fields. In which scenario(s), do inhibitory NOT-sSC projections suppress sSC activity? To address this, we mapped the receptive fields of individual sSC neurons using single-unit activity (Supplementary Fig. 12a,b, see Methods) and found that indeed the receptive field size of sSC neurons was highly variable, with 31.3% of receptive fields larger than 20° (Supplementary Fig. 12b,c). Interestingly, regardless of receptive field size, background gratings, which alone barely activated sSC neurons (Supplementary Fig. 12d,e,g,h), strongly suppressed their responses to black flashes (Supplementary Fig. 12d,f,g,i). Importantly, in both scenarios silencing inhibitory NOT neurons partially restored these flash-evoked responses (Supplementary Fig. 12d,f,g,i). Together, these results indicate that inhibitory NOT-sSC projections suppress flash-evoked sSC activity irrespective of receptive field size.

### Inhibitory NOT-sSC projections mediate the motion-induced impairment in visual detection

**Fig. 6:**
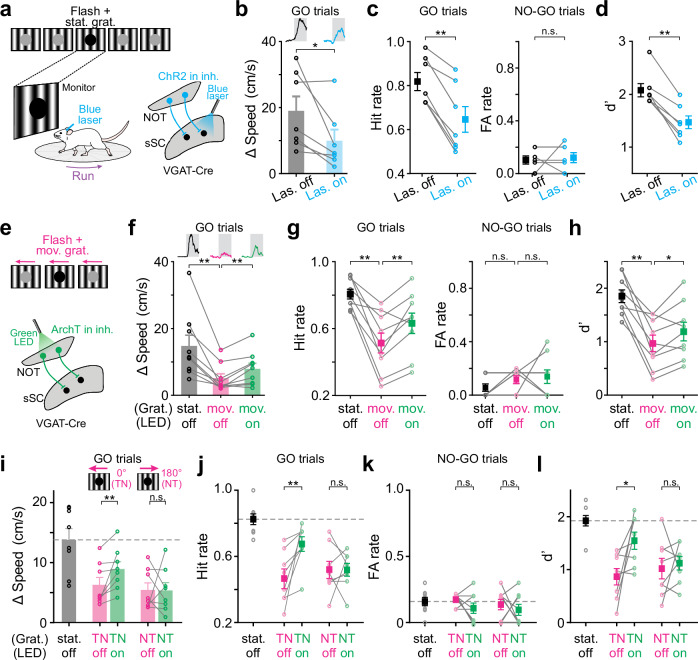
Inhibitory NOT-sSC projections mediate background motion-induced impairment in visual detection. **a**–**d** Inhibitory NOT-sSC projections can impair sSC-dependent visual detection. **a** Schematic of experimental setup. Note that inhibitory (inh.) NOT-sSC projections are activated by blue light illumination on ChR2-expressing NOT axons in the sSC. **b**–**d** Increment of mean running speed during black flashes (**b**), hit/false alarm rates (**c**), and d prime (**d**) under activation (Laser on) or control conditions (Laser off, *n* = 7 mice). Top inset in **b**, average speed traces in GO trials of an example animal. Shade width=3 sec (timing of flashes); shade height=14.8 cm/s. One-sided Wilcoxon signed-rank test, *p* = 0.016 for **b**, *p* = 0.0078 for hit rates in **c** and for **d**. Two-sided Wilcoxon signed-rank test, *p* > 0.99 for false alarm rates in **c**. **e**–**h** Inhibitory NOT-sSC projections contribute to background motion-induced impairment in visual detection. **e** Schematic of experimental setup. Note that inhibitory NOT neurons are silenced by inhibitory opsin ArchT. **f**–**h** Summary of behavioral parameters in 3 visual/optogenetic conditions (*n* = 9 mice). O*n*e-sided Wilcoxon signed-rank test; *p* = 0.002, static versus moving gratings with LED off in **f**, in hit rates in **g** and in **h**; *p* = 0.0039 (**f**), 0.0098 (**g**), 0.02 (**h**), LED off versus LED on with moving gratings. Two-sided Wilcoxon signed-rank test; *p* = 0.25, static versus moving gratings with LED off for false alarm rates in **g**; *p* > 0.99, LED off versus LED on with moving gratings for false alarm rates in **g**. Data presented as in **b**–**d**. **f** Shade width=3 sec (timing of flashes); shade height=46.7 cm/s. **i**–**l** Inhibitory NOT-sSC projections impair flash detection only during temporo-nasal background motion. Data presented as in **f**–**h**. Gray dash lines, mean values of behavioral parameters when static gratings surround flashes. One-sided Wilcoxon signed-rank test, *p* = 0.0039 for TN in **i** and **j**, *p* = 0.02 for TN in **l,** *p* = 0.27 for NT in **i**, *p* = 0.15 for NT in **j,** *p* = 0.32 for NT in **l**. Two-sided Wilcoxon signed-rank test*, p* = 0.22 for TN in **k**, *p* = 0.66 for NT in **k**. *p < 0.05; **p < 0.01. n.s., not significant. Grat., grating; mov., moving; stat., static. Data shown as mean ± s.e.m. TN, temporo-nasal. NT, naso-temporal.

Thus far, we have demonstrated that the inhibitory NOT-sSC projections suppress sSC activity in the context of background motion (Fig. 5), and that such motion suppresses sSC activity to impair visual detection (Fig. 1). Based on these findings, we hypothesized that the inhibitory NOT-sSC projections are responsible for the impaired visual detection observed during background motion. To directly test this hypothesis, we first optogenetically activated inhibitory NOT-sSC projections in expert mice by illuminating ChR2-expressing inhibitory NOT axons in the sSC (Fig. 6a) while the mice performed the task of detecting local flashes with a static-grating background. Consistent with the reduction in sSC activity upon activating inhibitory NOT neurons (Fig. 2h, i), activation of this inhibitory pathway significantly impaired animals’ task performance, as evidenced by reductions in running speed increment, hit rate and d’ (Δspeed: from 19.0 ± 4.4 to 9.9 ± 3.4 cm/s, hit rate: from 0.82 ± 0.04 to 0.65 ± 0.06, d’: from 2.08 ± 0.13 to 1.47 ± 0.12, Fig. 6b–d). These behavioral results resemble the effects of background motion on task performance (Fig. 1g–i), indicating that the inhibitory NOT-sSC pathway can indeed impair the detection of local luminance changes.

Next, to determine the necessity of this pathway for the motion-induced impairment of visual detection, we optogenetically silenced inhibitory NOT neurons while expert mice performed the visual task with a moving-grating background (Fig. 6e). This optogenetic perturbation partially rescued the behavioral deficits caused by background motion: running speed increment, hit rate and d’ in silencing trials were noticeably higher than in control trials (speed increment: 8.0 ± 1.7 vs 5.2 ± 1.3 cm/s, hit rate: 0.63 ± 0.06 vs 0.51 ± 0.06, d’: 1.19 ± 0.17 vs 0.97 ± 0.15, moving grating/LED on vs moving grating/LED off, Fig. 6f–h). Moreover, animals’ locomotor ability remained unaffected by either activating or silencing inhibitory NOT neurons (Supplementary Fig. 13), consistent with the finding that inhibitory NOT neurons project exclusively to visual processing structures, not locomotion-related areas (Supplementary Fig. 5b,c). Therefore, these results indicate that inhibitory NOT-sSC projections are indeed required for background motion to effectively impair the detection of flashes.

Last, since inhibitory NOT-sSC projections suppress flash-evoked sSC activity most effectively during temporo-nasal motion (Fig. 5j–m), we asked whether they would also impair flash detection most strongly in this context. To address this, we optogenetically silenced inhibitory NOT neurons while expert mice performed the visual detection task under either naso-temporal or temporo-nasal background motion. We found that although both motion directions markedly impaired task performance, optogenetic silencing partially rescued the behavioral deficits only when background gratings drifted temporo-nasally, but not naso-temporally (Fig. 6i–l). Collectively, these results indicate that inhibitory NOT-sSC projections play a significant role in the background motion-induced impairment in flash detection. Notably, under silencing conditions, background gratings moving either temporo-nasally or naso-temporally still impaired flash detection, suggesting that circuits other than the inhibitory NOT-sSC projections contribute to the motion modulation of visual perception. Overall, our findings uncover a previously unrecognized subcortical mechanism underlying the perceptual effects of global background motion: background motion recruits inhibitory NOT projections to disrupt subcortical visual processing, thereby impairing subcortically driven visual perception.

## Discussion

In this study, we found that NOT, a brainstem structure previously known for mediating reflexive eye movements, also modulates visual perception in the presence of background motion, a fundamental operation in vision. Importantly, we show that inhibitory projections from the NOT to sSC transmit a functionally specific signal encoding global background motion to suppress sSC visual processing, thereby compromising sSC-dependent visual perception in such motional context.

Our findings call for a revisit of the functional specialization of global motion processing systems^74,99–105^. Neural signals encoding global background motion emerge along two anatomically distinct pathways, the retina-dLGN-visual cortex pathway^32,106,107^ and retina-accessory optic system (including the NOT) pathway^108–110^. Traditionally, these two pathways are thought to support different visual functions. In the former pathway, visual cortices play a major role in motion-related perceptual phenomena^1–6,111–113^. In contrast, in the latter pathway, the NOT specializes in driving the optokinetic reflex (OKR), a reflexive compensatory eye movement^69–71^. However, our study demonstrates that the NOT also mediates the impairment of visual processing and perception by global background motion (Figs. 5, 6), independent of its role in reflexive eye movements (Supplementary Figs. 1i,j, 4b-e, 6b-e, 9b-e). Therefore, the NOT should be regarded not only as a sensory-motor interface for reflexive behaviors, but also as a visual processing center essential for visual perception. Moreover, since NOT and its projections to subcortical visual nuclei are conserved across vertebrates^114–118^, this brainstem structure may also underlie the motion modulation in non-mammalian species^61–66^, representing an evolutionarily conserved circuit mechanism.

The distinct anatomy and physiology of inhibitory NOT neurons (Figs. 2, 3, Supplementary Figs. 5, 7) suggest that this population has a unique visual function. Anatomically, inhibitory NOT projections avoid the pre-oculomotor brainstem nuclei (Supplementary Fig. 5c) that are innervated by excitatory NOT projections and involved in OKR^82,119,120^. Instead, inhibitory NOT projections target subcortical visual nuclei (Supplementary Fig. 5b) that are essential for visual perception but not for OKR^76,85,88–92,121^. Physiologically, the preference of sSC-projecting inhibitory NOT neurons for larger grating sizes (Fig. 3e–g) supports their role in contextual modulation by global motion, whereas the stronger temporo-nasal bias of the remaining NOT neurons (non-tagged, presumably excitatory projection neurons, Fig. 3i–l) aligns with their role in driving OKR behavior^120,122^. The anatomical and physiological specificities of sSC-projecting inhibitory NOT neurons support the idea that inhibitory NOT projections suppress visual processing and perception, but do not mediate reflexive eye movements, while excitatory projections function oppositely. Thus, this highlights a division of labor among distinct NOT outputs.

A classical receptive field in the visual cortex is typically surrounded by two concentric inhibitory regions: the near and far surrounds^97,98,123^, where background motion can suppress the activity evoked by stimuli within the receptive field (Fig. 5h schematic). We found that inhibitory NOT-sSC projections are selectively activated by large gratings (Fig. 3e–g) to suppress sSC activity in this context (Fig. 5f–i), indicating that this inhibition primarily represents the far, but not near, surround. This is reminiscent of previous reports that feedback inputs from motion sensitive cortical areas MT/MST form the far inhibitory surround in the primary visual cortex (V1)^97,124^. Despite this similarity, the surround suppression mediated by the NOT or MT/MST may differ in two ways. First, while the modulation of V1 activity by the feedback inputs from MT/MST emerges with a long delay (tens of milliseconds after V1 neurons are activated)^125–128^, the feedforward inhibitory inputs from the NOT arrive in the sSC and affect its activity without an obvious delay (Supplementary Fig. 7a). Second, MT/MST are highly plastic^129–131^, and therefore their feedback projections likely contribute to the experience-dependent adaptability of cortical contextual modulation. Conversely, NOT circuits are hard-wired^132,133^ and may support a reliable circuit operation. Overall, these two pathways may complement each other in motion modulation: NOT projections instinctively impact vision during the early onset of motion, while MT/MST projections adaptively influence vision during the later sustained period.

Motion-induced impairment in flash detection observed in this study (Fig. 1) is reminiscent of a common perceptual phenomenon in humans, motion-induced blindness (MIB), where small static objects intermittently disappear from visual awareness when embedded within global moving patterns^7,8^. Although often viewed as a failure of visual processing, MIB may instead arise from global motion processing^134,135^. For example, it has been proposed that the static objects can be occluded when the global moving patterns are filled in by surface completion or perceptual scotomas^134,136^. In this proposal, global motion processing acts as a suppressor to hinder the perception of the static objects, reflecting a competition between the systems computing global motion and local static objects^137–140^. Our findings align with this idea from several angles. First, in the brainstem global motion and local flashes are processed by distinct structures, since NOT neurons preferentially respond to global motion (Fig. 3)^72–75^, whereas sSC neurons favor local flashes (Fig. 1k,m, Supplementary Figs. 3b, 3f, 12e, 12h)^77,79,81^. Second, the NOT circuit processing global motion is linked to the sSC circuit processing local flashes by direct inhibitory projections (Fig. 2). Third, these projections convey global motion signals to the sSC (Fig. 3c–h), where they suppress local flash processing (Figs. 5, 6). Thus, like MIB, motion-induced impairment in flash detection may reflect the dominance of global motion processing. Additionally, similar precedence of global motion processing over local static processing is evident in other perceptual phenomena, including motion-induced fill-in^141–143^, motion-induced position shift^2,144^ and motion-induced contrast suppression^145,146^. Collectively, these global motion–related phenomena, including motion-induced impairment in flash detection, highlight the critical role of motion processing in supporting animal survival^26,32,147^.

The suppressive role of NOT revealed in this study is likely an intrinsic feature of subcortical circuits, independent of the visual cortex, despite the NOT receiving innervation from this cortical area^69,70,148^. First, the temporo-nasal direction bias of NOT neurons remains largely intact when visual cortical activity is disrupted^149,150^, suggesting that the motion signals carried by inhibitory NOT-sSC projections are inherited from the retina, but not from the cortex. Second, the effects of perturbing inhibitory NOT-sSC projections on sSC activity and sSC-dependent visual detection (Figs. 2, 5, 6) indicate that their direct inhibitory innervation of sSC neurons serves as a mechanism through which the NOT impairs visual perception. Nevertheless, since inhibitory NOT neurons also project to subcortical structures that innervate the visual cortex directly, like the dLGN and LP (Supplementary Fig. 5b), the NOT may also impair cortical-dependent perception.

Inhibitory NOT-sSC projections have a powerful impact on visual processing and the perception of local luminance changes (Figs. 5, 6). In addition to this fundamental visual feature, sSC circuits, which consist of different neuronal populations^84,94,151,152^, also effectively respond to other visual features, such as orientation, motion and looming^52,77,153–156^, and mediate various visual behaviors^76,83–87,157,158^. Since most sSC neurons receive direct inhibition from NOT-sSC projections (Fig. 2f), this inhibitory pathway is likely to influence sSC processing of other visual features and associated visual behaviors. For example, the activation of SC triggers contraversive eye and head movements in the orienting behavior, while the activation of NOT on the same side triggers ipsiversive eye movements in the OKR. Thus, when the NOT drives OKR eye movements to stabilize retinal images, its inhibitory projections to SC may function to counteract stimulus-triggered orienting behavior mediated by the SC.

## Methods

### Mice

All experimental procedures performed in this study were approved by the Biological Sciences Local Animal Care Committee, in accordance with guidelines established by the University of Toronto Animal Care Committee and the Canadian Council on Animal Care (protocol # 20012152 and 20013111).

We used the following mouse lines: Vgat-ChR2-EYFP^159^ (Jackson Laboratory #014548) for behavioral experiments involving silencing sSC neurons, Vgat-Cre^160^ (Jackson Laboratory #016962) for electrophysiology, behavioral and anterograde tracing experiments, Vglut2-Cre^160^ (Jackson Laboratory #028863) for experiments of activating eOPN3 in dLGN, GAD67-GFP^161^ for retrograde tracing, C57BL/6J (Jackson Laboratory #000664) and CD-1 (Charles River #022) for breeding purposes. Mice of both sexes aged from 3-5 months were used for most experiments, except that only females were used for retrograde tracing. Experimental mice were bred by crossing homozygous Vgat-Cre, Vgat-ChR2-EYFP or Vglut2-Cre males with wild-type CD-1 females, and heterozygous GAD67-GFP males with C57BL/6J females. Mice were housed in a vivarium with a reversed light cycle (12 h day/12 h night), ambient temperature at 22 °C, and humidity at 50%.

### Viral injections

We used the following adeno-associated viruses (AAV) for Cre recombinase (Cre) dependent expression of ChR2^162^, eGFP, ArchT^163^ and eOPN3^164^: AAV1-Ef1a-DIO-ChR2(E123T/T159C)-eYFP (AAV-DIO-ChR2, neurophotonics 648-AAV1), AAV1-CAG-FLEX-EGFP (AAV-FLEX-EGFP, Addgene 51502-AAV1), AAV1-CAG-FLEX-ArchT-EGFP (AAV-FLEX-ArchT, UNC), and AAV1-hSyn1-SIO-eOPN3-mScarlet-WPRE (AAV-SIO-eOPN3, Addgene 125713-AAV1). We used oG-pseudotyped G-deleted rabies virus B19-deltaG-mCherry-oG (RV-mCherry, neurophotonics 996-oG) to retrogradely trace the inputs to the sSC.

To optogenetically perturb inhibitory NOT neurons or NOT-sSC projections, 2-4-month-old Vgat-Cre mice were anesthetized with 1.5-2% isoflurane (vol/vol) in O_2_. The depth of anesthesia was monitored with the toe-pinch response. Eyes were protected with lubricant eye ointment (Systane, Alcon). The animal’s body temperature was maintained by a heating pad (HTP-1500, Kent Scientific). Carprofen (Rimadyl, Pfizer) was administered subcutaneously at a dose of 20 mg/kg to reduce pain. Furs over the head were shaved and the exposed skin was disinfected with 70% isopropyl alcohol and Chlorhexidine alcohol. The scalp was cut open and a small hole was drilled on the skull over the left NOT ( ~ 300–500 μm diameter), through which a beveled glass pipette (diameter 32–42 μm) was inserted into the NOT (Coordinates: anteroposterior axis (AP) relative to bregma, −2.7 mm; mediolateral axis relative to midline (ML), 1.13 mm; depth, 1.68 mm; the actual coordinates were calculated according to a standard bregma-lambda distance of 4.21 mm). A bolus of AAV-DIO-ChR2, AAV-FLEX-ArchT or AAV-SIO-eOPN3 (100nL) were then injected by a microinjection pump (UMP3T, WPI). Throughout the procedure, mice were subcutaneously injected with 1 mL of lactated ringer’s solution per hour (JB2324, BAXTER). After the completion of the viral injection, the scalp was sutured with sterile 6-0 nylon suture (916B, LOOK). For control experiments, a bolus of AAV-FLEX-EGFP (100 nl) was instead injected into the left NOT of Vgat-Cre mice. Experiments were performed 3-4 weeks after the viral injection.

To anterogradely trace the projection targets of inhibitory NOT neurons, AAV-FLEX-EGFP was injected into the left NOT of Vgat-Cre mice by an iontophoresis pump ( + 4 μA, 7 s on/7 s off, for 3 min, BAB-600, Kation Scientific) at postnatal 2–4 months. Mice were transcardially perfused 3-4 weeks after the virus injection.

To reveal inhibitory neurons that project to the sSC, a bolus of RV-mCherry (50 nL) was stereotaxically injected into the left sSC of GAD67-GFP mice (Coordinates: AP, -3.8 mm; ML, 0.5 mm; depth, 0.85 mm; the actual coordinates were calculated according to a standard bregma-lambda distance of 4.21 mm) at postnatal 2–4 months. Mice were transcardially perfused 5 days after the virus injection.

To examine the effectiveness of eOPN3 in projection-specific silencing, a bolus of AAV-SIO-eOPN3 (100 nL) was stereotaxically injected into the left dLGN of Vglut2-Cre mice (Coordinates: AP, −2.15 mm; ML, 2.25 mm; depth, 2.85 mm; the actual coordinates were calculated according to a standard bregma-lambda distance of 4.21 mm) at postnatal 2–4 months. Experiments were performed 3–4 weeks after the viral injection.

### Visually guided behavior

#### Animal preparation

A T-shaped head bar was implanted on an animal’s skull for head fixation. The temporalis muscle was separated from the skull to further increase the contact surface for attaching head bars. We scraped the skull to remove the fascia and cleaned it with PBS. After being dried with compressed air, the exposed skull was then covered with a thin layer of superglue (All Purpose, Krazy Glue). Then, the T-shaped head bar was glued onto the skull with acrylic resin (B1356, Lang Dental) mixed with black powder (iron oxide, Alpha Chemicals). Carprofen (dose 20 mg/kg) was administered daily for 3 days after the surgery. Animals were allowed to recover for 5–7 days, then were familiarized with head fixation and running on a round treadmill disk for at least 3 sessions before being used in experiments.

#### Visual stimulation

All visual stimuli used in this study were generated with PsychoPy (1.90.2)^165^ running in Python 2. Three computer monitors (Samsung S27R35AFHN or Acer BW257, refresh rate 60 Hz, gamma-corrected) were mounted orthogonally to each other to form a square enclosure. With a discrete graphic card (AMD Radeon Pro WX5100 or NIVIDIA GeForce RTX1660), three monitors were merged into a single display to ensure the frames were synchronized across multiple monitors. The enclosure covered ~270° of the visual space along the azimuth and 60° vertically. Animal’s head was fixed at the center of the enclosure, and its body axis was oriented 60° to the middle monitor so that this monitor is on the right side of the animal. The luminance of the three monitors was set to 40 cd/m^2^. During behavioral training, the target stimulus was a black circular patch (diameter, 20°; local luminance changes from 40 to 0 cd/m^2^ corresponding to a contrast of 100%) presented on the uniform background of the middle monitor at 45° in azimuth, and 11° in elevation to animals’ right eye (black flashes, GO trials). To estimate the false alarm and correct rejection rate, in 20% of randomly selected trials (80-125 total trials), no flash was presented (blank stimulus, NO-GO trials). GO and NO-GO trials had three consecutive phases: a pre-flash/blank period of 11–21 s (uniform distributions), a flash/blank period of 3 s, and a post-flash/blank period of 1 s. During behavioral testing, to examine the effects of moving backgrounds on this visually guided behavior, we added a sinusoidal grating (SF, 0.08 cpd; contrast, 100%; mean luminance, 40 cd/m^2^) outside the target stimulus region on the middle monitor. The left and right monitors had uniform background throughout the experiments. We made the grating move (TF, 2 Hz; temporo-nasal or naso-temporal direction in reference to animals’ right eyes) in half of the trials (GO and NO-GO) throughout the duration of the trial, and kept it static in the other half. Trials with static or moving gratings in the surrounds were randomly interleaved (total trials = 100 for experiments without optogenetics; 120 for experiments with optogenetics with background motion in a single direction, 150 for experiments with optogenetics with background motion in two directions). To examine the psychometric function of this detection task, we varied the contrast of the center flash randomly among 0%, 32%, 64%, 85%, 95% and 100%. Contrast = Δluminance/initial luminance. In the experiments of silencing sSC or activating inhibitory NOT-sSC projections, static gratings surrounded the region where black flashes were presented (flashes with static gratings) during both behavioral training and testing.

#### Behavioral training and testing

We designed a behavioral paradigm of visual detection based on a previous study^166^. In GO trials, when a local black flash appeared on the monitor, a head-fixed mouse was required to increase its instantaneous running speed by more than 10 cm/sec during the 3-s flash window in comparison to the average running speed during the 3-s window immediately before the flash onset (Hit); otherwise, the mouse would receive a tail shock by a stimulus isolator (ISO-FLEX, A.M.P.I., current 0.3 mA, duration 0.2 s, Miss) when the flash disappeared. If an animal was running when a flash appeared, it needed to run faster by at least 10 cm/s to avoid a tail shock. In NO-GO trials, the animal was spared from the electrical shocks, even if it ran during the 3-s blank stimuli. Since the SC is known to mediate visually guided avoidance/escape behaviors^167^, we chose this punishment-based training paradigm to mimic this SC function. There was no additional cue at the start of a trial, so animals would not know when a NO-GO trial started. When an animal reached a hit rate of ≥ 70%, its training was considered successful (expert). In the experiments involving optogenetic circuit perturbation, laser or LED was turned on in every other trial. Locomotion was monitored by an optical encoder (H5-360-IE-S, US Digital) connected to an Arduino board (Arduino Due). The control of the timing of electric shocks and optogenetics, the monitoring of the timing of visual stimulation and instantaneous running speed were carried out by the Arduino board running custom codes. All these signals were recorded by a data acquisition board (PCIE-6251, National Instruments) under the control of custom LabView software (National Instruments, 2014). Movement of animal’s right eye was monitored and recorded via an infrared camera (G3-GM12-M0640, Teledyne Dalsa, frame rate 120 Hz) during the whole period of each trial. All raw data were stored on a computer hard drive for further offline analysis.

### In vivo electrophysiological recording

#### Animal preparation

Two weeks after viral injection, a T-shaped head bar was stereotaxically implanted on a mouse’s skull for head fixation. The procedure of head bar implantation for in vivo electrophysiological recording was similar to the one for visually guided behavior, except that an inclinometer (551-1002-1-ND, Digi-Key electronics) was used to calibrate the inclination of the two axes of the T-shaped head bar relative to the AP and ML axes of the skull. And a fiber-optic cannula (200 µm in diameter, 0.39 NA, 3 or 3.5 mm in length, R-FOC-BL200C-39NA, RWD) was also inserted above either the left NOT or sSC (See details in “In vivo Optogenetics”). 1 week after the surgery, the mouse was familiarized with head fixation in the recording setup for 3 days without visual stimuli. One day before recording, we made a craniotomy of about 2 × 2 mm centered above the left NOT (AP, −2.7 mm; ML, 1.13 mm), sSC (AP, −3.75 mm; ML, 0.6 mm) or V1 (AP, −3.8 mm; ML, 2.7 mm) while the animal was anesthetized by 1.5–2% isoflurane. The actual coordinates were calculated according to a standard bregma-lambda distance of 4.21 mm. The exposed brain was kept moist and protected by the application of a drop of PBS and a piece of wet Gelfoam (344-ET1972, Surgifoam) on top of the craniotomy.

#### Recording

The next day, we head-fixed the animal on the recording setup. A 32-channel poly2 silicon probe (ASSY-37 H6b, Cambridge NeuroTech) mounted on a manipulator (7600-XYZL, Siskiyou) was slowly advanced into the left brain to a depth of 2000–2500 µm (NOT), 1300–1900 µm (sSC) or 450–550 µm (V1) from the surface of the brain. Electrophysiological signals were amplified, filtered and digitized at 30 kHz by an extracellular amplifier (C3315, Intan Technologies) and recorded by a USB interface board (C3100, Intan Technologies) under the control of Intan RHD 2000 interface V1_5_2. Locomotion and eye movement of the animal were recorded using an optical encoder (H5-360-IE-S, US Digital), an infrared camera (G3-GM12-M0640, Teledyne Dalsa, frame rate 120 Hz) and a data acquisition board (PCIE-6251, National Instruments) under the control of custom LabView software (National Instruments, 2014). All raw data were stored on a computer hard drive for further offline analysis.

#### Visual stimulation

One 27-inch monitor (ASUS VG279QM, refresh rate 60 Hz, gamma-corrected) was placed 19 cm away from an animal’s right eye. The monitor was placed with its center at 45° in azimuth and 11° in elevation and its surface perpendicular to the line connecting animal’s right eye and the monitor center. As a result, the monitor covered 112° horizontally and 80° vertically of animal’s visual space. To map spatial receptive fields (RFs) of multi-unit activity, the monitor was divided into a 6 × 8 grid with a spacing of 14° horizontally and 13.3° vertically. White (ON) and black (OFF) rectangles (14° horizontally × 13.3° vertically, 40 cd/m^2^ background luminance, ±100% contrast) were presented briefly (duration, 200 ms; interstimulus interval, 100 ms), one at a time within the 6 × 8 grid in a pseudo-random sequence. Each grid location was stimulated 10 times by ON stimuli and 10 times by OFF stimuli. To map spatial RFs of individual sSC neurons, two types of visual stimulation were used. First, as described above individual black rectangles were presented within the 6 × 8 grid for 20 repetitions. Second, small black squares (2°, 40 cd/m^2^ background luminance, 100% contrast) drifted along the rows and columns of the 6 × 8 grid at a speed of 30°/s. Each stimulus was repeated 10 times. If a sSC neuron was responsive to flashes, the first mapping method was used for RF analysis. Otherwise, the second mapping method was used for RF analysis. To evaluate the responses of NOT or sSC neurons to local luminance changes, the luminance of a round patch (diameter, 20°) surrounded by a static sinusoidal grating (SF, 0.08 cpd; contrast, 100%; mean luminance, 40 cd/m^2^, covering the area outside the patch on the screen) decreased (OFF, black flashes) or increased (ON, white flashes) briefly (duration, 0.5 s; interstimulus interval, 8 s). To overlay flashes on RF centers, animals’ eye movement was monitored during recording and the position of the flashing patch was adjusted accordingly in a trial-by-trial basis (see details in “Correction of the location of flash stimuli according to eye movement”). To examine the effects of moving backgrounds on flash-evoked sSC activity and the response properties of NOT neurons, we presented three types of visual stimulation: (1) flashes with static gratings in the surround; (2) flashes with moving gratings in the surround (TF, 2 Hz); (3) moving gratings in the surround only. Each type of visual stimulation occurred in 1/3 of randomized trials for 15-20 times. In the second type of visual stimulation, the grating motion started 0.5 s before the flash appeared and ended 0.5 s after it disappeared. In the third type, the surrounding grating drifted for 1.5 s without flashes.

There were some modifications of visual stimulation in other experiments. (1) To examine the effect of optogenetically activating inhibitory NOT neurons on flash-evoked sSC activity, flashes were presented on a uniform background (luminance, 40 cd/m^2^). (2) To examine the size selectivity of the motion-induced suppression of sSC activity, in types 1 and 2 of visual stimulation, the outer diameter of the surrounding gratings varied randomly among a set of pre-defined values (30°, 40°, 60°, 80° or full screen, which is equivalent to 107° in diameter). Instead of type 3 visual stimulation, we presented flashes on a uniform background (luminance, 40 cd/m^2^). (3) To examine the size selectivity of the motion-evoked NOT activity, in type 3 visual stimulation, the outer diameter of the surrounding gratings varied randomly among the same set of values as ***(2)***. Types 1 and 2 of visual stimulation were not presented. (4) To examine the direction selectivity of the motion-induced suppression of sSC activity, in types 1 and 2 of visual stimulation, the direction of grating movement varied randomly among 4 cardinal directions (upward, downward, temporo-nasal, naso-temporal). Type 3 was not presented. (5) To evaluate direction selectivity of NOT neurons, in type 3 visual stimulation, the surrounding gratings drifted in 12 evenly spaced directions. Types 1 and 2 of visual stimulation were not presented. (6) To examine the effectiveness of eOPN3 in projection-specific silencing, only type 1 visual stimulation was used.

#### Correction of the location of flash stimuli according to eye movement

During in vivo electrophysiological recording, the movement of an animal’s right eye was monitored by an infrared camera (G3-GM12-M0640, Teledyne Dalsa, frame rate 120 Hz) under the control of custom LabVIEW software (National Instruments, 2014). The eye pupil in the video was detected online by a two-step process: (1) use a thresholding algorithm to estimate the center of mass of the pupil; (2) from the estimated pupil center, run a one-step starburst algorithm to profile the boundary of the pupil. The eye position was calculated based on the distance between the pupil center and the corneal reflection of a reference IR LED placed along the optical axis of the camera. To calibrate the measurement of the eye movements, the camera and the reference IR LED were moved together by ±10° along a circle centered on the eye^69^. In each trial, the eye position during the time window of 0.9–1 s before the flash stimulation was used to update the location of the gray circular patch on the monitor at the end of this window so that local flashes were centered on RFs regardless the eye movements between trials. If the eye position measured during the flash stimulation differed from that measured during the 0.9–1 s update window ( >4°), or if the eye moved during the flash stimulation in nystagmus (amplitude >4° and speed >25°/s) or slow drift (displacement >4°), the corresponding trials were excluded from analysis.

### In vivo optogenetics

To perturb the activities of inhibitory NOT neurons or their projections to the sSC, we virally expressed ChR2, ArchT or eOPN3 in inhibitory neurons in the left NOT of Vgat-Cre mice. In controls, eGFP was virally expressed in inhibitory NOT neurons. To perturb sSC activity, we used the Vgat-ChR2-EYPF line. In all these experiments, we stereotaxically implanted fiber-optic cannulas (200 µm diameter, 0.39 NA, 3 or 3.5 mm in length, R-FOC-BL200C-39NA, RWD) to deliver laser or LED light to the left NOT or sSC. These cannulas were tilted 20° laterally in the coronal plane relative to the vertical axis (coordinates to target NOT: AP, −2.7 mm; ML, 2 mm; depth, 1.86 mm; coordinates to target sSC: AP, −3.75 mm; ML, 1.85 mm; depth, 1.4 mm; the actual coordinates were calculated according to a standard bregma-lambda distance of 4.21 mm), and secured in place by acrylic resin (B1356, Lang Dental). To test the effectiveness of eOPN3, instead of implanting a fiber-optic cannula, we thinned the skull above the visual cortex of Vglut2-Cre mice which had eOPN3 expressed in their dLGN axons, and illuminated this area with a fiber placed ~5–10 mm above the skull surface. Various light sources were used in optogenetic perturbations: for ChR2, a blue laser (0.8 mW, 488 nm MDL-SD-488 or 470 nm MDL-III-470, Opto Engine LLC); for ArchT and eOPN3, a green LED (1.2 mW for experiments of silencing inhibitory NOT neurons or projections, 10 mW for silencing dLGN-V1 projections, 530 nm, M530F3, Thorlabs). In the experiments of perturbing inhibitory NOT neurons or inhibitory NOT-sSC projections, the laser or LED was turned on in every other trial. The timing of optogenetics varied in individual experiments: (1) in behavioral experiments, the laser or LED was on during the flash or blank stimuli; (2) to examine the effect of activating inhibitory NOT neurons on flash-evoked sSC activity, the laser was turned on 0.5 s before the onset of flashes, and lasted for 1.5 s; (3) to examine the effects of silencing inhibitory NOT neurons or projections on flash-evoked sSC activity, the LED was turned on 0.5 s before the onset of moving gratings (1 s before flashes) and lasted for 2 s; (4) in the experiments of optogenetically tagging sSC-projecting inhibitory NOT neurons^120^, a train of five blue laser pulses (0.8 mW, 5-ms pulse width, 10 Hz) illuminated the ChR2-experessing NOT axons in sSC 1 s after the completion of visual stimulation in every other trial. The laser pulses triggered antidromic spikes in ChR2-expressing NOT axons in the sSC, which were recorded by electrodes inserted into the NOT and recognized by their little jitter and short onset latency. The timing and power of photo stimulation were controlled by a data acquisition board (PCIE-6251, National Instruments) and custom Labview software (National Instruments, 2014).

### Slice electrophysiological recording

To examine whether inhibitory NOT-sSC projections synapse on sSC neurons, we virally expressed ChR2 in inhibitory NOT neurons of Vgat-Cre mice. Three weeks after viral injections, under urethane anesthesia (1.5 g/kg) animals were transcardially perfused with an ice-cold (0 °C) cutting solution ((in mM) 92 NMDG, 2.5 KCl, 1.2 NaH2PO4, 30 NaHCO3, 20 HEPES, 25 glucose, 5 Na ascorbate, 2 thiourea, 3 Na pyruvate, 10 MgSO4, 0.5 CaCl2, pH 7.4, 305 mOsm, saturated with 95% O2/5% CO2) and decapitated^168^. The brain was sectioned into 300-µm coronal slices in cold cutting solution with a vibrating microtome (VT1200, Leica). The slices containing the SC were first incubated in a submersion chamber filled with warm (35 °C) cutting solution for 30 min. During this incubation period, we gradually added 4 mL of 2 M NaCl into the cutting solution to increase its Na concentration to 87 mM. After the initial incubation period, brain slices were transferred to and incubated in the external solution ACSF ((in mM) 126 NaCl, 26 NaHCO3, 10 glucose, 2.5 KCl, 2 CaCl2, 1 MgCl2, and 1.25 NaH2PO4, 2 Na Pyruvate, 0.5 Na ascorbate, pH 7.4, 295 mOsm, saturated with 95% O2/5% CO2) at room temperature for at least 1 hour, until used for recording.

Whole-cell recordings were done in a chamber (RC-27LD, Warner Instruments) perfused by ACSF of room temperature at 3 mL/min. sSC neurons were visualized with DIC infrared video-microscopy under a water immersion objective (40X, 0.8 NA) on an upright microscope (Olympus BX51WI) with an IR CCD camera (IR-2000, DAGE-MTI). Whole-cell voltage-clamp recordings were performed on sSC neurons with glass pipettes ( ~ 4–6 MΩ resistance, BF150-110-10, Sutter Instruments) filled with a cesium-based internal solution ((in mM) 115 CsMeSO4, 1.5 MgCl2, 10 HEPES, 0.3 Na-GTP, 4 Mg-ATP, 10 Na2-phosphocreatine, 1 EGTA, 2 QX-314-Cl, 10 BAPTA-tetracesium, pH 7.35, 295 mOsm). IPSCs were recorded at a holding voltage of 0 mV with the addition of tetrodotoxin (TTX, 1 μM, Hello Bio, HB1035) and 4-aminopyridine (4-AP, 1 mM, Abcam, AB120122) to the external solution ACSF to isolate monosynaptic responses^169^. To photo-stimulate ChR2-expressing NOT axons in sSC, we delivered blue light using a collimated LED with a blue filter (470/40X filter, SOLIS-1C, thorlabs) through the fluorescence illuminator port and the 40X objective. Light pulses of 10 ms and 2.8 mW/mm2 were repeated with an inter-stimulus interval of 10 s for 10–15 times. In a few recordings, 5 μM gabazine was added to the ACSF to validate that GABA_A_ receptors mediated these IPSC responses. Signals were filtered at 4 kHz with a Multiclamp 700B amplifier and digitized with a Digidata 1440A at 10 kHz under the control of Clampex 10.2 (Molecular Devices). Data were analyzed offline with Clampfit 10.2 (Molecular Device) and MATLAB (R2019b, Mathworks).

To examine the effectiveness of ArchT in silencing inhibitory NOT neurons, ArchT was virally expressed in inhibitory NOT neurons of Vgat-Cre mice. Three weeks after viral injections, brain slices containing NOT were prepared as described above. Whole-cell current-clamp recordings were performed on ArchT-expressing NOT neurons with glass pipettes of similar size filled with a potassium-based internal solution ((in mM) 125 K-gluconate, 1.5 MgCl2, 10 HEPES, 0.3 Na-GTP, 4 Mg-ATP, 10 Na2-phosphocreatine, 1 EGTA, pH 7.3; 295 mOsm). During recordings, green light from the same collimated LED with a green filter (545/25X filter, 2.5 mW/mm2 power, 10–15 trials, SOLIS-1C, Thorlabs) illuminated the area containing the patched neurons. To measure the strength of hyperpolarization mediated by ArchT, we illuminated the slice for 10 ms without current injection. To evaluate the effectiveness of ArchT in suppressing spiking activity, we injected currents of 0.6–1.2 nA into the patched neurons for 0.9 s, and the LED was turned on for 0.3 s in the middle of current injection.

### Histology, anatomical tracing, and imaging

3 weeks after AAV injection or 5 days after rabies virus injection, under anesthesia (1.5 g/kg urethane; intraperitoneal injection) mice were perfused transcranial first with PBS and then with 4% paraformaldehyde (PFA, AC416785000, Fisher Scientific) in PBS (pH 7.4). Brains were dissected from the skull, post-fixed overnight in 4% PFA, and then immersed in 30% sucrose in PBS for 24 h. The fixed brains were coronally sectioned into 100-µm slices with a sliding Microtome (HM450, Thermo Scientific). The harvested brain slices were stained with DAPI (0.05 µg/mL in PBS, D1306, Invitrogen) for 10 min and then washed 3 times with PBS for 5 min each round. The slices were mounted in a mounting medium (0100-35, Southern biotech). Confocal images were acquired on a Zeiss LSM 880 confocal microscope (software: Zeiss Zen 2.3 SP1). ImageJ (National Institutes of Health) was used to process images and count fluorescent neurons. Brain structures were delineated according to Allen mouse brain atlas (atlas.brain-map.org).

### Data analysis

#### Behavioral data

The increment of mean running speed during flashes (∆speed) was calculated by subtracting the average running speed in the 3-s window before flash/blank stimulation from the average running speed during 3-s flash/blank stimulation. Hit rate was calculated as the proportion of GO trials in which instantaneous running speed increased by more than 10 cm/sec during the 3-s flash window in comparison to the average running speed in the 3-s window immediately before the flash onset (Hit trials). False alarm rate was calculated as the proportion of NO-GO trials in which instantaneous running speed increased by more than 10 cm/sec during the 3-s blank window in comparison to the average running speed in the 3-s window immediately before the blank onset (FA trials). d’ was calculated as Z(Hit rate)-Z(FA rate), where Z(Hit rate) and Z(FA rate) are the Z-scores of Hit and FA rates, respectively. If FA rate was 0, FA rate of 1 over the number of NO-GO trials was used to calculate Z(FA Rate). The behavioral onset latency in Hit trials was quantified by the time when the instantaneous running speed exceeded the average speed in the 3-s window before flash stimulation by 10 cm/s. Early sessions meant the first two training sessions; intermediate sessions meant the middle two training sessions; late sessions meant the last two training sessions. The psychometric curves were fitted with a modified cumulative Weibull function:

1 $${{{\rm{Hit\; rate}}}}={{{\rm{low}}}}+({{{\rm{high}}}}-{{{\rm{low}}}})\times (1-{e}^{-{({{{\rm{contrast}}}}/\lambda )}^{k}})$$

where low and high are low and high saturation points, *λ* is the scale parameter, and *k* is the shape parameter.

#### Timing and amplitude of IPSCs

Onset latency of optogenetically evoked IPSCs in sSC neurons was determined as the time lag between the start of LED illumination and the time point at which the IPSC amplitude reached 2.5 times the standard deviation of baseline noise. Trial-by-trial jitter of optogenetically evoked IPSCs was calculated as the standard deviation of the onset latency. Amplitude of IPSCs was defined as the peak of IPSC responses in the 100-ms window after the start of LED illumination, in reference to the baseline.

#### Unit isolation

Spike waveforms from raw data were sorted and clustered using a spike-sorting algorithm (kilosort2: https://github.com/MouseLand/Kilosort)^170^. Then, clusters were manually merged, split, and cleaned with phy 2.0 beta 1 (https://github.com/cortex-lab/phy/)^171^ based on the similarity in the waveform, correlogram, channel position, and visual feature selectivity. Clusters with their refractory period violations less than 0.2% were considered to be single units. The multi-unit activity was defined as all spiking events exceeding the detection threshold after removing electrical noise or movement artifacts by the sorting algorithm. All units were assigned a depth according to the electrode sites where the amplitudes of their spikes were the largest. A strong temporo-nasal preference of multi-unit activity was used to determine the boundary of the NOT. Strong flash-evoked multi-unit activity was used to determine the boundary of the sSC.

#### Firing rate

For both single-unit activity and multi-unit activity, the visual responses to flashes or moving gratings were computed as the mean firing rates in the windows of 50–200 ms after the onset of flashes or in the whole period of grating motion, respectively, after subtracting the mean spontaneous firing rates (350 ms before flashes or 500 ms before the movement of gratings, respectively). Two criteria were used to define responsive neurons: (1) their firing rates evoked by preferred stimuli were 1 spike/s or higher; (2) and their evoked firing rates were three standard deviations higher than the spontaneous firing rates. Only sSC neurons that were responsive to flashes were included in analysis. Only NOT neurons that were responsive to moving gratings were included in analysis.

#### Onset latency of spiking responses to flashes or gratings

To quantify the onset latency of flash-evoked responses in sSC or grating-evoked responses in NOT, we first derived the peristimulus time histograms (PSTHs) of single units with 5-ms bins. The onset latency was defined as the time when the evoked firing rate exceeded three times standard deviation of the spontaneous firing rate. A unit was included in the analysis when its evoked activity was at least 2 spikes per bin within 150 ms from the onset of flashes or gratings.

#### Population averaged normalized SUA

To quantify the effects of background motion and/or circuit perturbation on flash-evoked sSC activity, evoked firing rates of individual neurons under various visual stimulation/optogenetic conditions were first normalized to their firing rates to flashes only or flashes with static gratings, and then averaged. To examine the size selectivity of the motion-induced suppression of sSC activity, evoked firing rates were normalized to the average firing rate evoked by flashes without background motion (flashes only and flashes with static gratings of various sizes). To quantify the response properties of NOT neurons, evoked firing rates of individual neurons were normalized to their firing rates evoked by full-screen moving gratings in the surround (107°; Fig. 3d,f) or by preferred directions (Fig. 3j), before averaging.

#### Onset latency and jitter of optogenetically triggered antidromic spikes

The onset latency of optogenetically triggered antidromic spikes was defined as the mean of their spike timing relative to the start of individual laser pulses. Trial-by-trial jitter of these spikes was calculated as the standard deviation of their spike timing. Units with onset latency less than 7 ms and jitter less than 2 ms were considered tagged neurons (sSC projecting inhibitory NOT neurons).

#### Receptive field size of sSC neurons

For RF mapping using black flashing squares, the evoked firing rate at each grid location was calculated by subtracting the spontaneous firing rate (50 ms before flashes) from the mean firing rate during flash stimulation. For RF mapping using moving black squares, the firing rate evoked by stimulus presented at each grid location was calculated by square root of the product of evoked firing rates moving horizontally and vertically across each grid location.

To quantify the receptive field size of SC units, we performed a 2D Gaussian fit using the following function:

2 $$F\left(x,y\right)=	 A\times \exp \left(-\frac{1}{2}\left[\frac{{\left(\left(x-{x}_{0}\right)\times \cos \left({{\emptyset }}\right)+\left(y-{y}_{0}\right)\times \sin \left({{\emptyset }}\right)\right)}^{2}}{{\sigma }_{x}^{2}}\right.\right. \\ 	 \left.\left.+\frac{{\left(-\left(x-{x}_{0}\right)\times \sin \left({\emptyset}\right)+\left(y-{y}_{0}\right)\times \cos \left({\emptyset}\right)\right)}^{2}}{{\sigma }_{y}^{2}}\right]\right)+B$$

where *F(x, y)* is the evoked firing rates of stimulus presented at the coordinates (*x*, *y*), *A* is the amplitude, *B* is the baseline, *x*_*0*_ and *y*_*0*_ define the center of the Gaussian function, *σ*_*x*_ and *σ*_*y*_ are the half widths of the short axis and long axis, and *ϕ* is the orientation angle of the short or long axis. The RF size (degree in diameter) was calculated as:

3 $${{{\rm{RF\; Size}}}}=2\times \sqrt{{\sigma }_{x}\times {\sigma }_{y}}$$

#### Size tuning of NOT neurons

To characterize the dependence of NOT activity on the size of surrounding moving gratings, we performed the following analysis.

(1) Linear regression: we performed linear regression with MATLAB function ‘polyfit’ to derive the slope of size tuning curves of linear regression analysis.

(2) Weighted preferred size: Weighted preferred size was calculated as:

4 $${{{\rm{Weighted\; pref}}}}.{{{\rm{size}}}}=\frac{{\sum }_{k}(R\left({{{{\rm{size}}}}}_{k}\right)\times {{{{\rm{size}}}}}_{k})}{{\sum }_{k}R\left({{{{\rm{size}}}}}_{k}\right)}$$

where size_*k*_ is the size of surrounding moving gratings, and *R(*size_*k*_*)* is the firing rate evoked by the gratings of corresponding size_*k*_.

(3) Size selectivity index: Size selectivity index SSI_max vs min_ was calculated as:

5 $${{{{\rm{SSI}}}}}_{\max {{{\rm{vs}}}}\min }=\frac{({R}_{max }-{R}_{min })}{(|{R}_{max }|+{|R}_{min }|)}$$

where *R*_max_ and *R*_min_ are the highest and lowest firing rates, respectively of a NOT neuron in response to surrounding moving gratings of various sizes.

Size selectivity index SSI_107 vs 30_ was calculated as:

6 $${{{{\rm{SSI}}}}}_{107\,{{{\rm{vs}}}}\,30}=\frac{({R}_{107}-{R}_{30})}{(|{R}_{107}|+|{R}_{30}|)}$$

where *R*_107_ is the firing rate of a NOT neuron evoked by full-screen (107°) surrounding moving gratings, and *R*_30_ is its firing rate evoked by surrounding moving gratings of 30°.

#### Direction tuning of NOT neurons

To characterize the dependence of NOT activity on the direction of surrounding moving gratings, we performed the following analysis.

(1) Vector analysis: Global direction selectivity index (gDSI) and preferred direction were calculated as:

7 $${{{\rm{gDSI}}}}=\frac{|{\sum }_{k}R({\rho }_{k})\times {e}^{i{\rho }_{k}}|}{{\sum }_{k}|R\left({\rho }_{k}\right)|}$$

8 $${\rho }_{{{{\rm{pref}}}}}={{\arg }}\left({\sum}_{k}R({\rho }_{k})\times {e}^{i{\rho }_{k}}\right)$$

where *R(ρ*_*k*_*)* is the response amplitude to the kth direction *ρ*_*k*_. Only neurons with *gDSI* more than 0.05 were used in the analysis of preferred direction.

(2) Direction selectivity index (*DSI*): the sample direction of the drifting grating (0°, 30°, 60°, 90°, 120°, 150°, 180°, 210°, 240°, 270°, 300°, 330°) that evoked largest response was defined as the preferred direction and the direction opposite to it was defined as the null direction. Direction selectivity index (*DSI*) was calculated as:

9 $${DSI}=\frac{({R}_{{pref}}-{R}_{{null}})}{(|{R}_{{pref}}|+|{R}_{{null}}|)}$$

where *R*_pref_ is the firing rate at the preferred direction, and *R*_null_ is the firing rate at the null direction.

#### Quantification of the effect of silencing inhibitory NOT neurons on sSC activity

∆SUA of sSC neurons upon silencing inhibitory NOT neurons was defined as percentage change in population-averaged normalized SUA. The modulation index of these neurons was calculated as:

10 $${{{\rm{Modulation}}}}\; {{{\rm{Index}}}}=\frac{({R}_{{{{\rm{LED}}}}\; {{{\rm{on}}}}}-{R}_{{{{\rm{LED\; off}}}}})}{(|{R}_{{{{\rm{LED}}}}\; {{{\rm{on}}}}}|+{|{R}}_{{{{\rm{LED}}}}\; {{{\rm{off}}}}}|)}$$

where *R*_LED on_ is the firing rate in LED on trials, and *R*_LED off_ is the firing rate in LED off trials.

### Statistical analysis

MATLAB (R2019b, Mathworks) was used to do statistical analyses and to plot cumulative probability curves in Supplementary Figs. 8f, 10j and receptive fields in Supplementary Fig. 2b. GraphPad Prism 6.1 was used to plot all other figures. All error bars were presented as mean ± s.e.m. unless otherwise noted. For small data size, statistical significance was assessed with Wilcoxon signed rank test or Wilcoxon rank sum test unless otherwise noted. For Fisher’s exact test in Fig. 3f, neurons with preferred sizes of 30° or 40° were classified as neurons preferring small size and the neurons with preferred sizes of 80° or 107° were classified as neurons preferring large size. For Fisher’s exact test in Fig. 3l, neurons with preferred directions from −15° to 45° were classified as neurons preferring temporo-nasal directions and the rest were classified as neurons preferring non-temporo-nasal directions. To compare the cumulative probability curves in Supplementary Fig. 8f, 10j, two-sample Kolmogorov-Smirnov test (ks-test) was performed. The relationship between the normalized SUA and the grating size in Fig. 4c and Supplementary Fig. 7b,c was fitted by a linear function (R= a*x + b), where R is the normalized SUA and x is the grating size. The relationship between the recovery of sSC activity upon optogenetic silencing and the suppression of sSC activity by background motion in Fig. 5e and Supplementary Fig. 10k was fitted by a linear function (R = a*x + b), where R is the level of recovery and x is the level of suppression. Statistic tests and *p* value for linear correlation were performed using the MATLAB function ‘corr’.

### Reporting summary

Further information on research design is available in the Nature Portfolio Reporting Summary linked to this article.

## Supplementary information

Supplementary Information

Description of Additional Supplementary Files

Supplementary Movie 1

Supplementary Movie 2

Supplementary Movie 3

Supplementary Movie 4

Reporting Summary

Transparent Peer Review file

## Source data

Source Data

## Data Availability

All original datasets generated and/or analyzed in this study are not deposited in online repositories due to their enormous size (over 5 TB), but available from the corresponding author upon request. All data used to generate the figures and statistics are provided in the source data file. Source data are provided with this paper.

## Code availability

The code for the analyses can be accessed from the following repository: https://github.com/xiaolinchou/active-avoidance-task-and-create-spikes-after-Kilosort^172^.
