# Supplementary material for: An inhibitory brainstem pathway reduces visual detection during background motion
Source: Nat Commun. 2026 May 7;17:6168. doi: 10.1038/s41467-026-72619-x (PMC13365429; doi:10.1038/s41467-026-72619-x)
Supplement: Supplementary file 1 — Supplementary Information [file 41467_2026_72619_MOESM1_ESM.pdf]

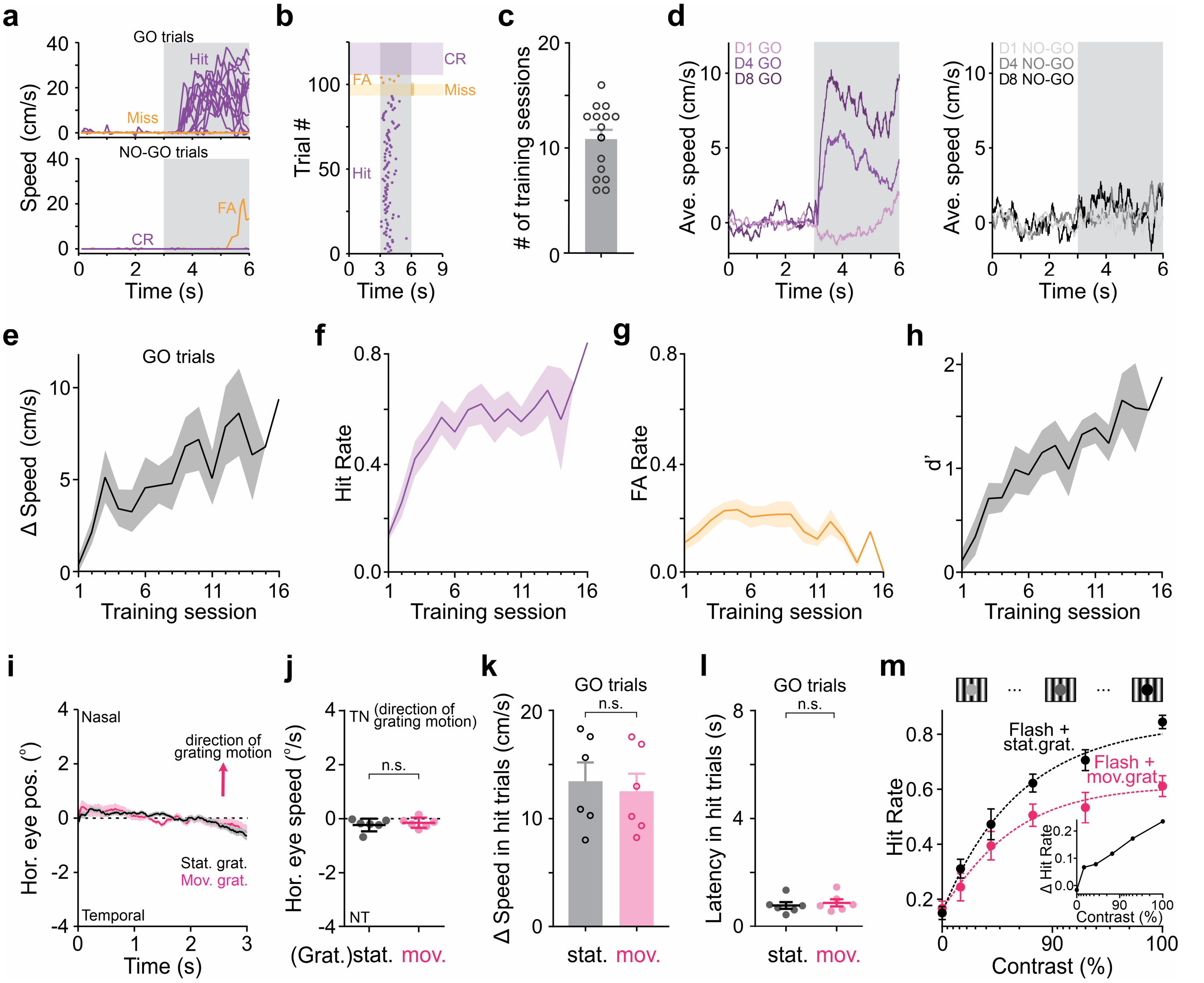

Supplementary Figure 1 | Visual task of detecting local luminance changes. a, Speed traces of example trials of 4 behavioural contingencies. Shade, timing of black flashes or blank stimuli. *n*=13 (Hit), 3 (Miss), 1 (False alarm, FA), 3 (Correct rejection, CR) trials. **b**, Raster plot of the timing of behavioural actions (•) and electrical shocks (○) in an example late training session. Note that trials are sorted according to behavioural contingencies. Grey shade, timing of black flashes or blank stimuli. *n*=125 trials. **c**, Summary of the number of training sessions before reaching a hit rate of >70% (*n*=15 mice). **d**, Average speed traces of GO (left) and NO-GO trials (right) of an example animal in early (Day 1), intermediate (Day 4), and late (Day 8) training sessions. Grey shade, timing of black flashes or blank stimuli. **e-h**, Population-averaged increment of mean running speed during black flashes (**e**), hit rate (**f**), false alarm rate (**g**) and d prime (d', **h**) across training sessions (*n*=15 mice). **i**, Trajectories of horizontal eye movements of an example animal's right eye in trials with static or moving gratings. Note that surrounding gratings move temporo-nasally (pink arrow) in reference to the right eye, which sees the visual stimulation, during the 3 second window. **j**, Summary of the speed of horizontal eye movements in the 3-second window before the flash onset (*n*=6 mice, two-sided Wilcoxon signed-

rank test, *p*=0.31). TN, temporo-nasal; NT, naso-temporal. **k**,**l**, Comparison of running speed increment (**k**) and onset latency (**l**) in hit trials between static-grating and moving-grating conditions (*n*=6 mice, two-sided Wilcoxon signed-rank test, *p*=0.69 for **k**, *p*=0.16 for **l**). **m**, Contrast psychometric curves of hit rate in the flash detection task with static (black) or moving (pink) background gratings. Dashed lines, the best fits using a modified Weibull function. Inset, Contrast psychometric curves of hit rate change caused by background motion. n.s., not significant. Traces and summary data shown as mean±s.e.m.

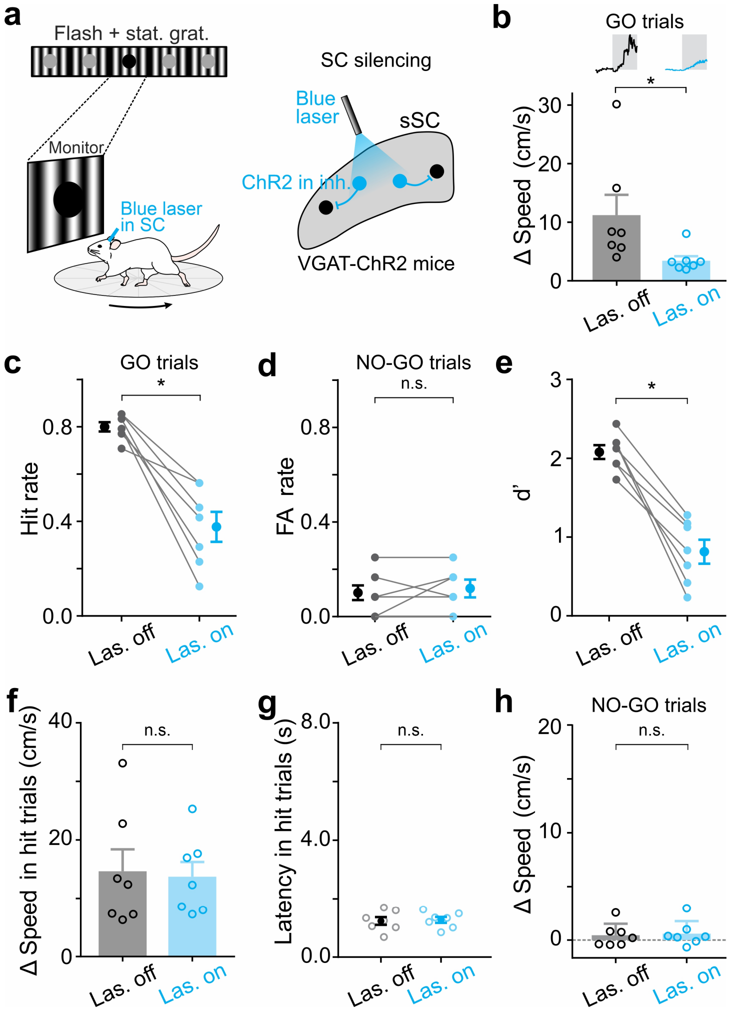

Supplementary Figure 2 | Visual detection of local luminance changes depends on the sSC. a, Schematic of experimental setup. Note that sSC activity is silenced by photo-stimulation of ChR2-expressing inhibitory sSC neurons and that a static grating surrounds black flashes or blank stimuli in all trials. **b-h**, Comparison of behavioural parameters between control (Laser off) and silencing trials (Laser on). *n*=7 mice. **b**, Increment of mean running speed during black flashes. Top inset, average speed traces in GO trials of an example animal. Shade width=3 sec (timing of black flashes); shade height=15.0 cm/s. One-sided Wilcoxon signed-rank test, *p*=0.016. **c**, Hit rate. One-sided Wilcoxon signed-rank test, *p*=0.016. **d**, False alarm rate. Two-sided Wilcoxon signed-rank test, *p*=0.75. **e**, d prime. One-sided Wilcoxon signed-rank test, *p*=0.016. **f**, Increment of mean running speed during black flashes in hit trials. Two-sided Wilcoxon signed-rank test, *p*=0.81. **g**, Behavioural onset latency in hit trials. Two-sided Wilcoxon signed-rank test, *p*=0.69. **h**, Increment of mean running speed during blank stimuli in NO-GO trials. Two-sided Wilcoxon signed-rank test, *p*=0.69. *, *p*<0.05. n.s., not significant. Data shown as mean±s.e.m.

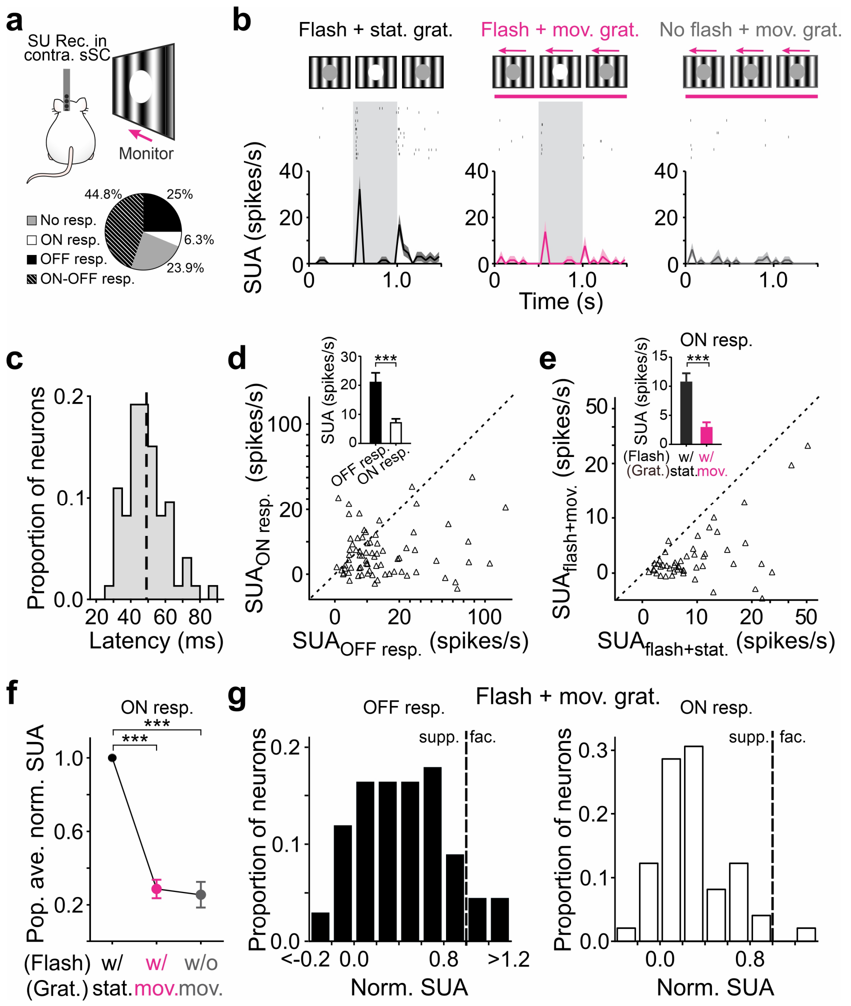

Supplementary Figure 3 | Background motion suppresses sSC activity evoked by flashes. **a**, Top, schematic of single-unit (SU) recording from contralateral (contra.) sSC. Pink arrow, grating motion. Bottom, proportion of sSC neurons responsive to white flashes (ON resp.), black flashes (OFF resp.), both (ON-OFF resp.), or neither (No resp.). *n*=96 neurons. **b**, Single-unit activity (SUA) of an example sSC neuron evoked by white flashes with static gratings (left), white flashes with moving gratings (middle) and moving gratings only (right). Top, schematic of visual stimulation. Pink arrow, grating motion. Bottom, raster plots and peristimulus time histograms (PSTHs) of spikes. Pink bars represent the timing of moving gratings. Shades, timing of flashes. **c,** Histogram of onset latency of sSC responses to black or white flashes. Vertical dashed line, population-averaged onset latency. *n*=73 neurons. **d**, Comparison of single-unit activity (SUA) evoked by white flashes (ON) to that evoked by black flashes (OFF). Dashed line, unity line. Inset, population-averaged SUA (*n*=73 neurons, one-sided Wilcoxon signed-rank test, *p*=5.0E-07). **e**, Comparison of SUA evoked by white flashes between moving-grating trials and static-grating trials. Dashed line, unity line. Inset, population-averaged SUA (*n*=49 neurons, one-sided Wilcoxon signed-rank test, *p*=8.3E-10). **f,** Population-averaged normalized SUA evoked by white flashes with static gratings, white flashes with moving gratings, or moving

gratings only (*n*=49 neurons, one-sided Wilcoxon signed-rank test, *p*=7.3E-10 for flash with static gratings versus flash with moving gratings, *p*=1.2E-09 for flashes with static gratings versus moving gratings only). **g**, Histograms of SUA evoked by black flashes (OFF, left) or white flashes (ON, right) with moving gratings normalized to that with static gratings (*n*=67 vs 49 neurons, black vs white flashes). Black dashed line, normalized SUA of 1.0 representing no change in sSC responses. Supp., suppression. Fac., facilitation. ***, $p<0.001$. Traces and summary data shown as mean±s.e.m.

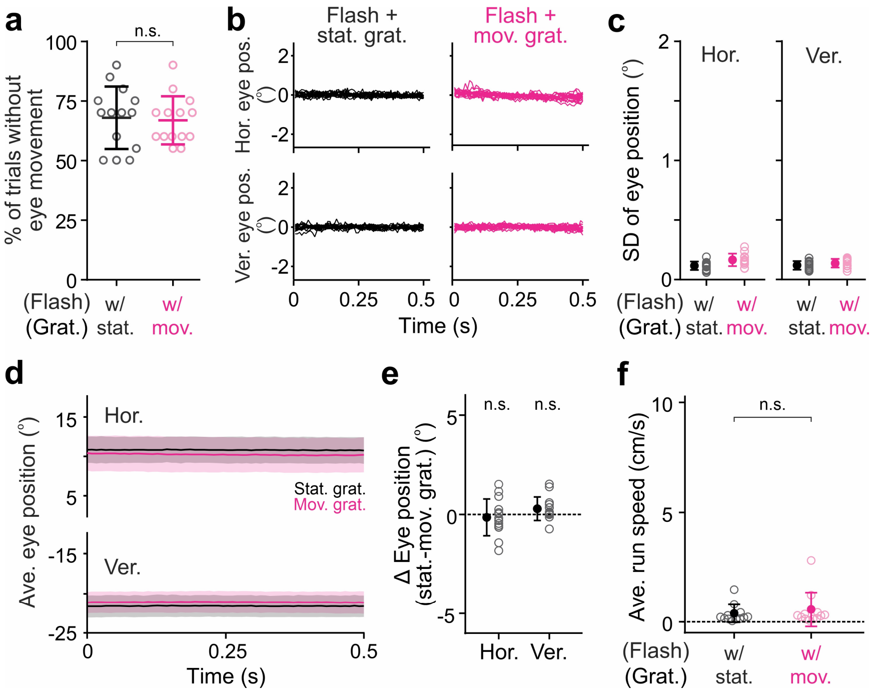

Supplementary Figure 4 | Suppressive effects of background motion on sSC activity cannot be explained by eye movements or locomotion. a, Percentage of static (stat.) grating (grat.) and moving (mov.) grating trials in which there is no eye movement. Note that trials with eye movements are excluded in data analysis. Two-sided Wilcoxon signed-rank test, p=0.87. **b**, Traces of horizontal (Hor., top) and vertical (Ver., bottom) eye positions (pos.) during flash stimulation in trials without eye movements from an example recording. Note that the baselines are subtracted to overlay traces. **c**, Comparison of standard deviation (SD) of horizontal (left) and vertical (right) eye positions during flash stimulation in trials without eye movements between moving-grating and static-grating conditions. **d**, Trial-averaged traces of horizontal (top) and vertical (bottom) eye positions of an example recording. **e**, Summary of the difference in horizontal and vertical eye positions between static-grating trials and moving-grating trials. Two-sided one sample Wilcoxon signed-rank test, p=0.50 for horizontal movements, p=0.10 for vertical movements. **a-e**, n=14 recordings. **f**, Comparison of trial-averaged running speed during flash stimulation between static-grating and moving-grating trials (n=12 recordings, two-sided Wilcoxon signed-rank test, p=0.11). w/, with black flashes. n.s., not significant. Traces and summary data shown as mean±s.d.

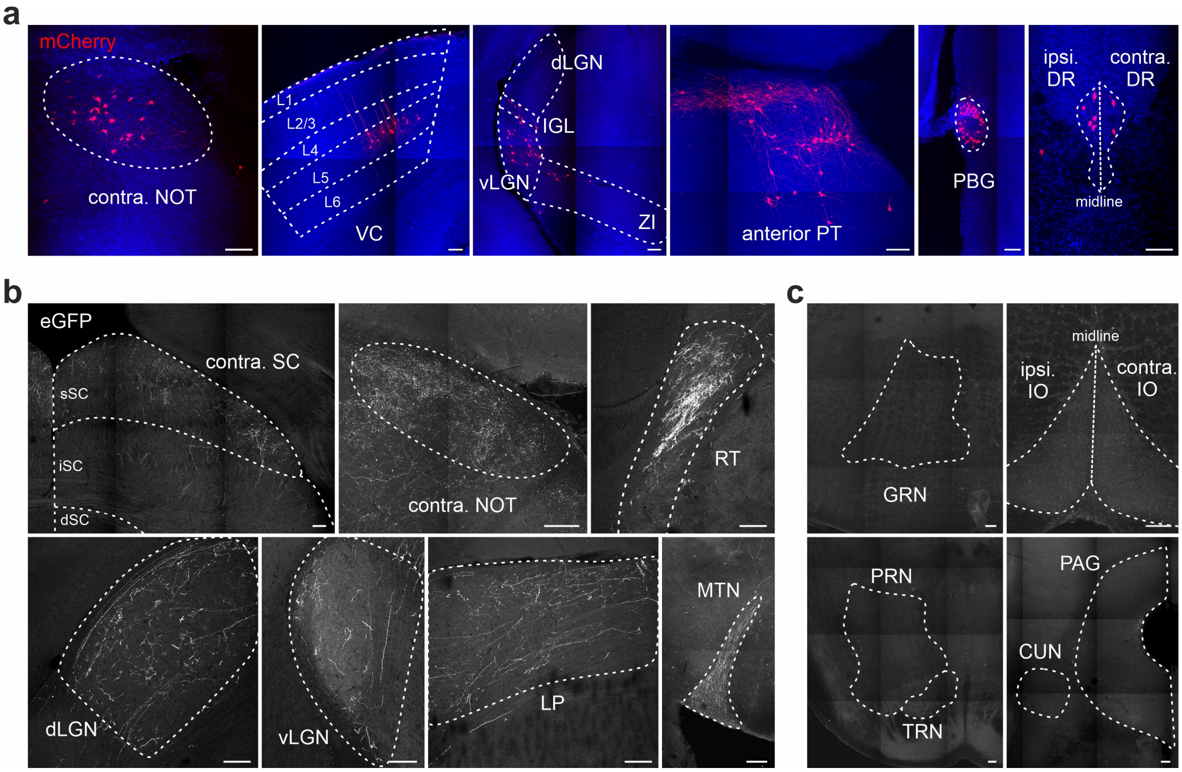

Supplementary Figure 5 | Anatomical tracing of the afferent inputs to the sSC and the efferent outputs of inhibitory NOT neurons. a, Coronal slices of structures, in addition to ipsilateral NOT, in which neurons are retrogradely labeled by the injection of RabV-mCherry into the sSC (*n*=3 mice). Slices come from the same experiment in **Figure 2a-b**. Red, mCherry. Blue, DAPI. contra. NOT, contralateral NOT; VC, visual cortex (L1-6, layers 1-6); dLGN, dorsal lateral geniculate nucleus; IGL, intergeniculate leaflet; vLGN, ventral lateral geniculate nucleus; ZI, zona incerta; anterior PT, anterior pretectal area; PBG, parabigeminal nucleus; DR, dorsal raphe; ipsi., ipsilateral; contra., contralateral. **b**, Coronal slices of structures, in addition to ipsilateral SC, to which inhibitory NOT neurons project (*n*=3 mice). Slices come from the same experiment in **Figure 2c-d**. contra. SC, contralateral SC; iSC, intermediate SC; dSC, deep SC; RT, reticular nucleus of the thalamus; LP, lateral posterior nucleus of the thalamus. MTN, medial terminal nucleus. **c**, Coronal slices of motor-related structures from the same experiment in **Figure 2c-d**. Note that these structures receive no projection from inhibitory NOT neurons. GRN, gigantocellular reticular nucleus; IO, inferior olive; PRN, pontine reticular nucleus; TRN, tegmental reticular nucleus; PAG, periaqueductal gray; CUN, cuneiform nucleus. Scale bar, 100 µm.

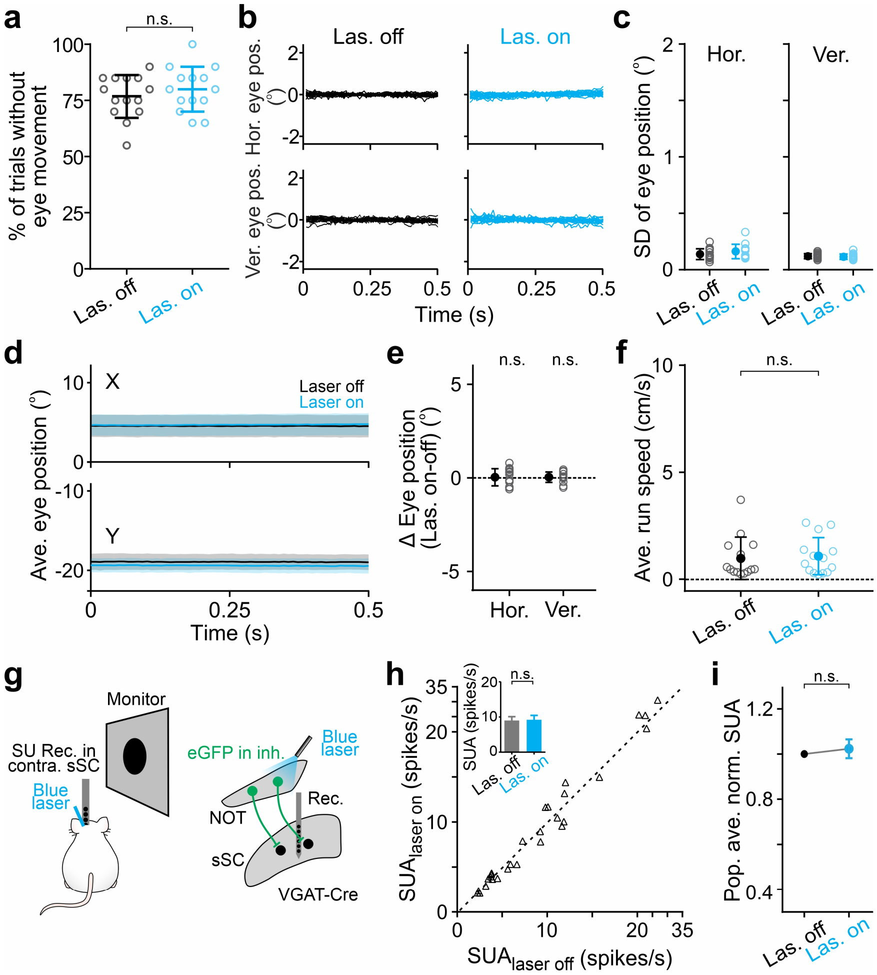

Supplementary Figure 6 | Suppression of sSC activity by the activation of inhibitory NOT neurons cannot be explained by eye movements, locomotion or blue light illumination. a, Percentage of control (Laser off) and activation trials (Laser on) in which there is no eye movement. Note that trials with eye movements are excluded in data analysis. Two-sided Wilcoxon signed-rank test, p=0.17. **b**, Traces of horizontal (Hor., top) and vertical (Ver., bottom) eye positions (pos.) during flash stimulation in trials without eye movements from an example recording. Note that the baselines are subtracted to overlay traces. **c**, Comparison of standard deviation (SD) of horizontal (left) and vertical (right) eye positions during flash stimulation in trials without eye movements between optogenetic (Laser on) and control (Laser off) conditions. **d**, Trial-averaged traces of horizontal (top) and vertical (bottom) eye positions of an example recording. **e**, Summary of the difference in horizontal and vertical eye positions between control (Laser off) and activation trials (Laser on). Two-sided one sample Wilcoxon signed-rank test, p=0.90 for horizontal movements, p=0.39 for vertical movements. **f**, Comparison of trial-averaged running speed during flash stimulation between control (Laser off) and activation (Laser on) trials. Two-sided Wilcoxon signed-rank test, p=0.86. **a-f**, n=14 recordings. **g-i**, Blue light illumination in controls does not affect sSC activity. **g**, Schematic of experimental setup in controls. Note that eGFP is expressed in inhibitory NOT neurons and black flashes are presented on a uniform

background while single-unit recording is carried out in contralateral sSC. **h**, Comparison of single-unit activity (SUA) in response to black flashes between laser on and laser off trials. Dashed line, unity line. Inset, population-averaged SUA. Two-sided Wilcoxon signed-rank test, *p*=0.90. **i**, Population-averaged normalized flash-evoked SUA with or without laser illumination. Two-sided Wilcoxon signed-rank test, *p*=0.90. **h**,**i**, *n*=29 neurons. n.s., not significant. Traces and summary data in **a-**f shown as mean±s.d. Data in **h-i** shown as mean±s.e.m.

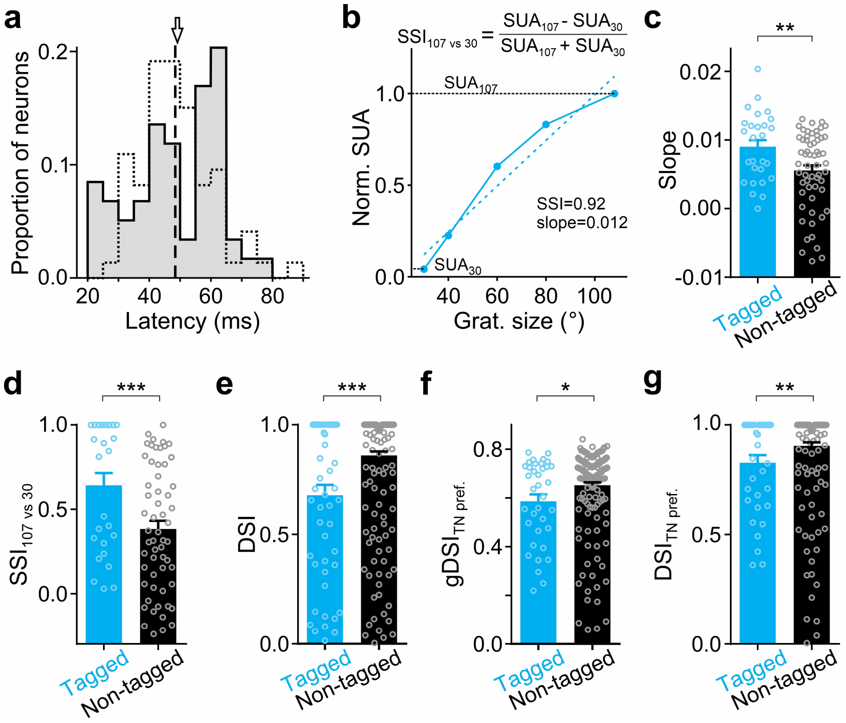

Supplementary Figure 7 | sSC-projecting inhibitory NOT neurons have unique response properties. **a**, Histogram of onset latency of the activity of sSC-projecting inhibitory NOT neurons evoked by full-screen (107°) background gratings moving temporo-nasally (solid line). Vertical dashed line, population-averaged onset latency. *n*=59 neurons. For comparison purpose, data of sSC responses to flashes from **Supplementary Figure 3c** is superimposed (dotted line) and their population-averaged onset latency is marked by an arrow. **b**, Quantification of the size tuning curve of an example sSC-projecting inhibitory NOT neuron (solid line). Blue dashed line, best-fit line of linear regression. Top, formula to calculate size selectivity index$_{107\ vs\ 30}$ (SSI$_{107\ vs\ 30}$). **c**,**d**, Summary of the slope of linear regression (**c**) and SSI$_{107\ vs\ 30}$ (**d**) of tagged (sSC projecting inhibitory NOT neurons, *n*=27 neurons) and non-tagged neurons (*n*=58 neurons). One-sided Wilcoxon signed-rank test, *p*=0.0074 for **c**, *p*=0.0007 for **d**. **e**, Summary of direction selectivity index (DSI; *n*=54 vs 197 neurons, tagged vs non-tagged). **f**,**g**, Summary of global DSI (gDSI) (**f**) and DSI (**g**) of temporo-nasally preferring neurons (TN pref., -22.5° < preferred direction < 67.5°). *n*=36 vs 177 neurons, tagged vs non-tagged. One-sided Wilcoxon signed-rank test, *p*=0.0001 for **e**, *p*=0.015 for **f**, *p*=0.005 for **g**. *, *p*<0.05; **, *p*<0.01; ***, *p*<0.001. Data shown as mean±s.e.m.

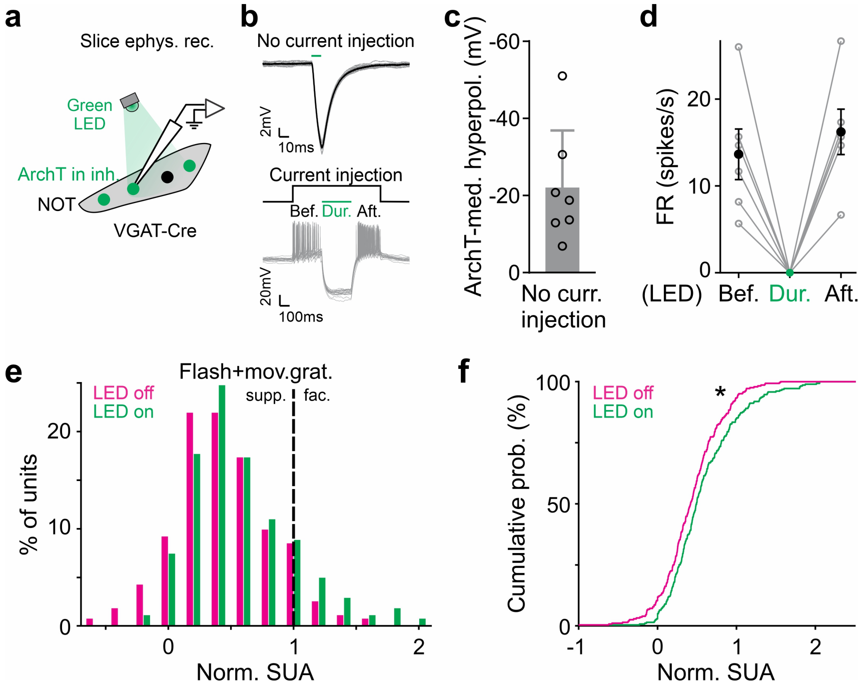

Supplementary Figure 8 | Effective silencing of inhibitory NOT neurons alleviates the suppression of sSC activity by background motion. a-d, Examination of ArchT-based silencing of inhibitory NOT neurons with slice electrophysiology. **a**, Schematic of experimental setup. Note that recording is done on ArchT-expressing inhibitory NOT neurons. **b**, Example recordings. Top, hyperpolarization mediated by the activation of ArchT. Bottom, spiking activity evoked by current injection (0.8 nA) before (Bef.), during (Dur.) and after (Aft.) green light illumination. Grey, individual traces; black, average trace. Green bar, timing of green LED. Black square waveform, time course of current injection. **c**, Summary of ArchT-mediated hyperpolarization (n=7 neurons) with no current (curr.) injection. Data shown as mean±s.d. **d**, Summary of firing rate (FR) evoked by current injection before, during and after green light illumination (n=6 neurons). **e**,**f**, Effects of silencing inhibitory NOT neurons on sSC activity evoked by flashes during background motion (n=283 neurons). **e**, Histograms of flash-evoked single-unit activity (SUA) with moving gratings (mov. grat.) normalized to that with static gratings, under silencing (LED on) or control conditions (LED off). Note that optogenetic silencing right-shifts the histogram, indicating a relief of suppression. Black dashed line, normalized SUA of 1.0 representing no change in sSC responses. Supp., suppression. Fac., facilitation. **f**, Cumulative probability (prob.) of normalized SUA (one-sided ks-test, p=0.012) under silencing (LED on) or control conditions (LED off). Traces and summary data in **d**,**f-h** shown as mean±s.e.m.

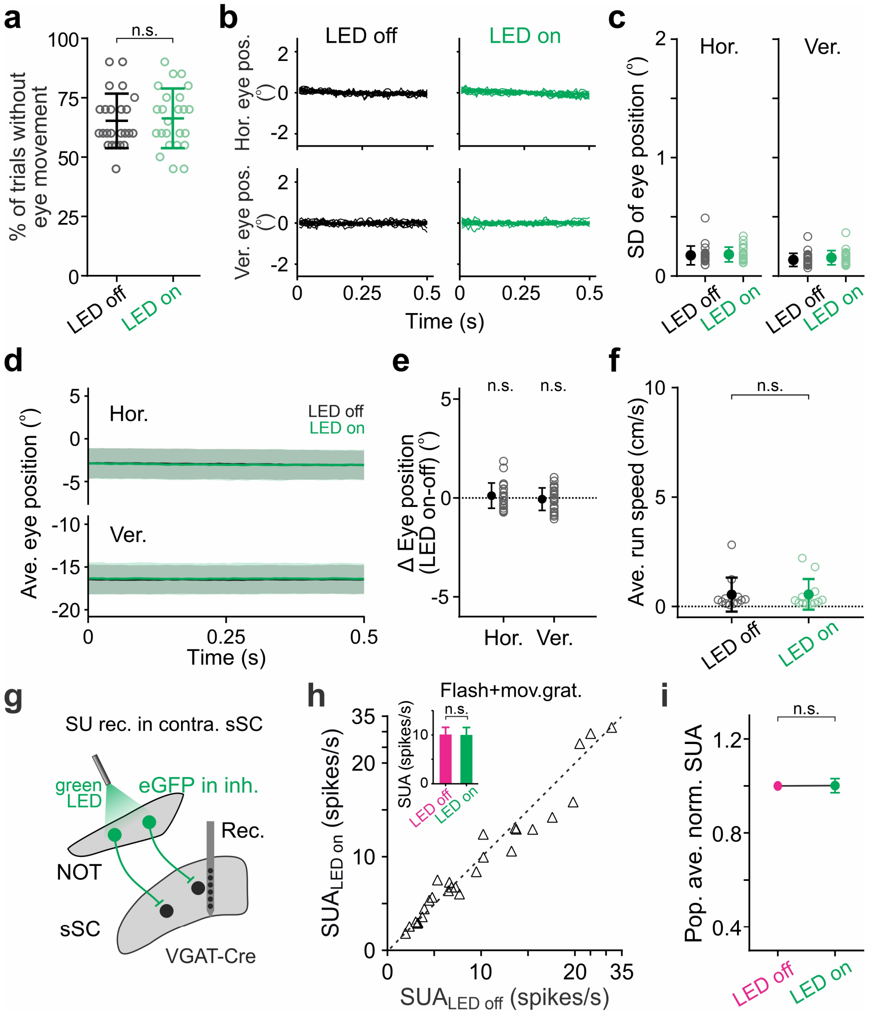

Supplementary Figure 9 | Facilitatory effects of silencing inhibitory NOT neurons on sSC activity cannot be explained by eye movements, locomotion or green light illumination. a, Percentage of control (LED off) and silencing trials (LED on) in which there is no eye movement. Note that trials with eye movements are excluded in data analysis. Two-sided Wilcoxon signed-rank test, p=0.63. **b**, Traces of horizontal (Hor., top) and vertical (Ver., bottom) eye positions (pos.) during flash stimulation in trials without eye movements from an example recording. Note that the baselines are subtracted to overlay traces. **c**, Comparison of standard deviation (SD) of horizontal (left) and vertical (right) eye positions during flash stimulation in trials without eye movement between optogenetic (LED on) and control (LED off) conditions. **d**, Trial-averaged traces of horizontal (top) and vertical (bottom) eye positions of an example recording. **e**, Summary of the difference in horizontal and vertical eye positions between control (LED off) and silencing trials (LED on). Two-sided one sample Wilcoxon signed-rank test, p=0.47 for horizontal movements, p=0.77 for vertical movements. **a-e**, n=24 recordings. **f**, Comparison of trial-averaged running speed during flash stimulation between control (LED off) and silencing trials (LED on, n=12 recordings, two-sided Wilcoxon signed-rank test, p=0.85). **g-i**, Green light illumination in control does not affect sSC activity (n=27 neurons). **g**, Schematic of experimental setup

in controls. Note that eGFP is expressed in inhibitory NOT neurons and single-unit recording is carried out in contralateral sSC. **h**, Comparison of single-unit activity (SUA) in response to black flashes with moving gratings between LED on and LED off trials. Two-sided Wilcoxon signed-rank test, p=0.48. Dashed line, unity line. Inset, population-averaged SUA. **i**, Population-averaged normalized SUA with or without LED illumination. Two-sided Wilcoxon signed-rank test, p=0.73. n.s., not significant. Traces and summary data in **a-f** shown as mean±s.d. Data in **h-i** shown as mean±s.e.m.

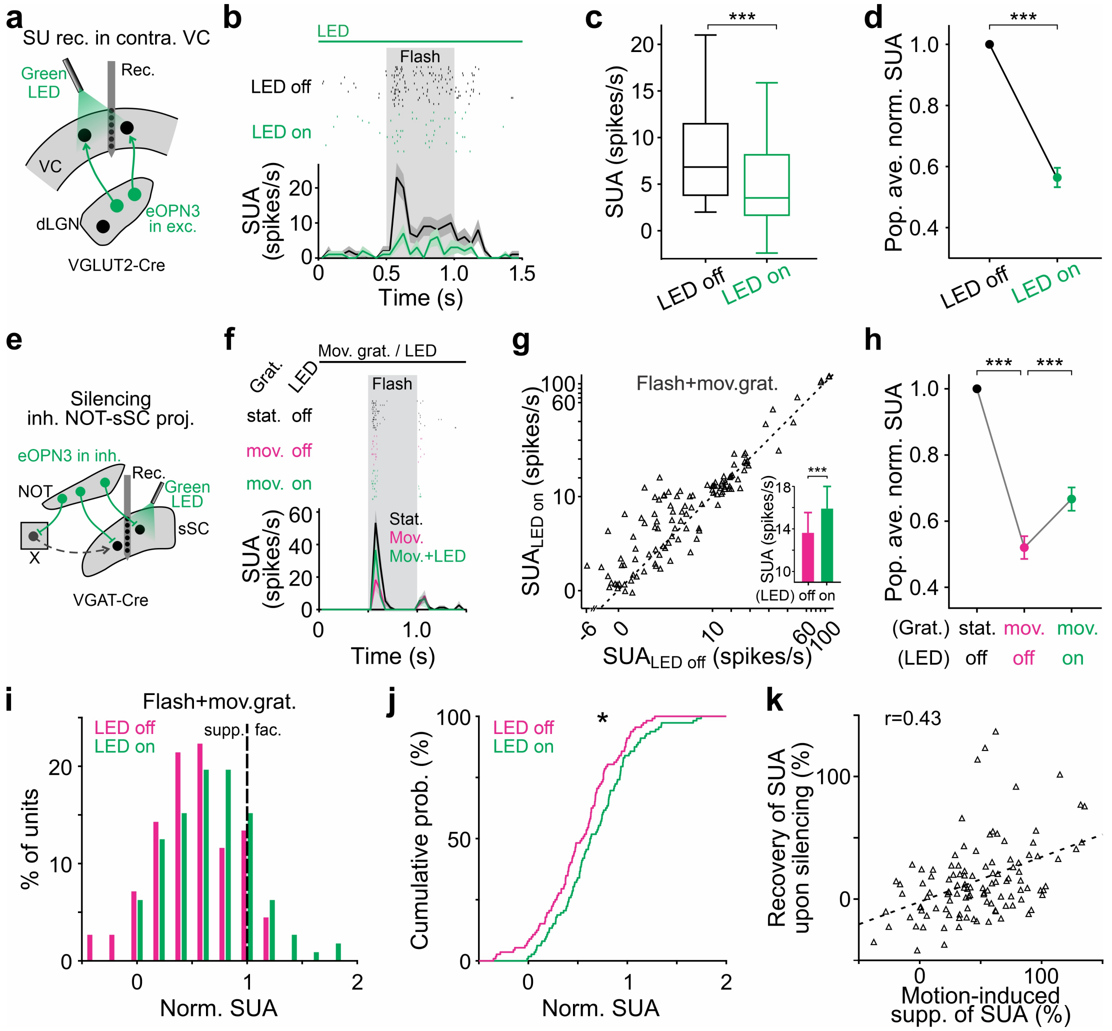

Supplementary Figure 10 | Silencing inhibitory NOT-sSC projections alleviates the suppression of sSC activity by background motion. a-d, Examination of eOPN3-based silencing with *in vivo* single-unit recording from contralateral visual cortex (VC). **a**, Schematic of experimental setup. Note that green LED illuminates excitatory (exc.) eOPN3-expressing dorsal lateral geniculate nucleus (dLGN) axons in the visual cortex and that visual cortical neurons are stimulated by black flashes. **b**, Single-unit activity (SUA) of an example visual cortical neuron. Top, raster plot; bottom, peristimulus time histogram (PSTH). Shade, timing of flashes. **c**,**d**, Tukey box-whisker plot of SUA (**c**) and population-averaged normalized SUA (**d**) with and without LED illumination (n=79 neurons, one-sided Wilcoxon signed-rank test, p=2.6E-12 for **c**, p=1.7E-14 for **d**). Center line, box edges and whiskers in Tukey box-whisker plot represent median, 25% and 75% quantile and minimum and maximum values within 1.5 times interquartile range from the quartile. **e-k**, Contribution of inhibitory NOT-sSC projections to the suppression of sSC activity by background motion (n=112 neurons). **e**, Schematic of experimental setup. Note that inhibitory NOT-sSC projections are silenced by inhibitory opsin eOPN3. X, nuclei downstream of inhibitory NOT neurons. Dashed arrowhead line, the excitatory polysynaptic pathway

connecting X with sSC. **f**, Raster plots (top) and peristimulus time histograms (PSTHs, bottom) of flash-evoked spiking responses of an example sSC neuron under three visual/optogenetic conditions. Shade, timing of black flashes. Bar, timing of moving gratings and LED. Grat., grating; stat., static; mov., moving. **g**, Comparison of SUA in response to flashes with moving gratings between silencing (LED on) and control conditions (LED off). One-sided Wilcoxon signed-rank test, *p*=1.2E-06. Dashed line, unity line. Inset, population averaged SUA. **h**, Population averaged normalized flash-evoked SUA under three visual/optogenetic conditions. One-sided Wilcoxon signed-rank test, *p*=3.6E-18 for static versus moving gratings with LED off, *p*=1.9E-06 for LED off versus LED on with moving gratings. **i**, Histograms of flash-evoked SUA with moving gratings (mov. grat.) normalized to that with static gratings, under silencing (LED on) or control conditions (LED off). Note that the silencing right-shifts the histogram, indicating a relief of suppression. Black dashed line, normalized SUA of 1.0 representing no change in sSC responses. Supp., suppression. Fac., facilitation. **j**, Cumulative probability (prob.) of normalized SUA (one-sided ks-test, *p*=0.025) under silencing (LED on) or control conditions (LED off). **k**, Correlation between the recovery in sSC activity upon silencing inhibitory NOT-sSC projections and the suppression of sSC activity exerted by background motion. Dashed line, best fit line of linear regression. r, correlation coefficient of the linear regression (one-sided linear regression analysis, *p*=1.1E-06). *, *p*<0.05; ***, *p*<0.001. Traces and summary data shown as mean±s.e.m.

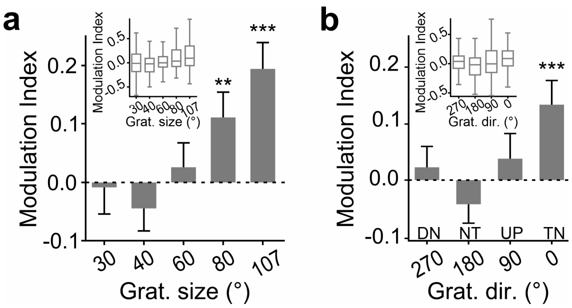

Supplementary Figure 11 | Modulatory effects of inhibitory NOT-sSC projections on sSC activity depend on the size and direction of moving gratings in the surrounds. a, Modulation index of sSC neurons upon silencing inhibitory NOT neurons depends on grating size (Grat. size, *n*=76 neurons). Inset, Tukey box-whisker plot of the data. One-sided one sample Wilcoxon signed-rank test, *p*=0.0042 for size 80°, *p*=5.1E-05 for size 107°. **b**, Modulation index of sSC neurons upon silencing inhibitory NOT neurons depends on grating direction (Grat. dir., *n*=82 neurons). DN, downwards. NT, nasotemporal. UP, upwards. TN, temporonasal. One-sided one sample Wilcoxon signed-rank test, *p*=0.0009 for TN. Inset, Tukey box-whisker plot of the data. Center line, box edges and whiskers in Tukey box-whisker plot represent median, 25% and 75% quantile and minimum and maximum values within 1.5 times interquartile range from the quartile. **, *p*<0.01, ***, *p*<0.001. Data shown as mean±s.e.m.

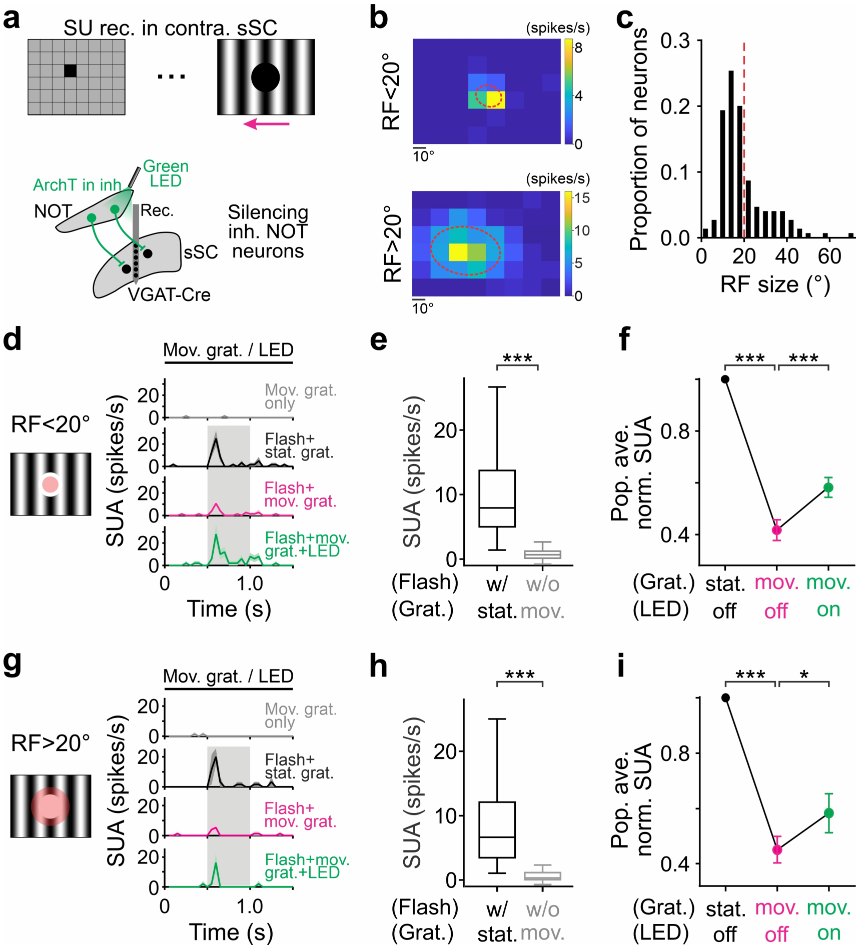

Supplementary Figure 12 | Inhibitory NOT-sSC projections suppress flash-evoked sSC activity regardless of sSC receptive field size. **a**, Schematic of experimental design. Note that single-unit recording is carried out in contralateral sSC. The receptive fields of individual sSC neurons are mapped using square black flashes presented over a 6x8 grid (top left) before examining the circular flash-evoked sSC activity under four optogenetic/visual conditions (top right). Inhibitory NOT neurons are silenced by ArchT. **b**, Example sSC receptive field smaller than 20° (top) and larger than 20° (bottom). **c**, Distribution of receptive field size of sSC neurons. Red dashed line, the criterion of defining small (<20°) or large receptive fields (>20°). *n*=170 neurons. **d**-**f**, Silencing inhibitory NOT neurons relieves the suppression of flashed-evoked sSC activity when background motion appears outside small (<20°) sSC receptive fields. **d**, Peristimulus time histograms (PSTHs) of single-unit activity (SUA) of an example sSC neuron with a small receptive field (<20°) under four optogenetic/visual conditions. Shade, timing of black flashes. Bar, timing of moving gratings and LED. Grat., grating; stat., static; mov., moving. Left, schematic of a receptive field smaller than 20° (red) overlayed on the background grating stimulation. **e**, Tukey box-whisker plot of SUA of sSC neurons with receptive fields smaller than 20° evoked by black flashes or moving background gratings. Center line, box edges and whiskers represent median, 25% and 75% quantile and minimum and maximum values within 1.5 times

interquartile range from the quartile. One-sided Wilcoxon signed-rank test, p=1.2E-20. **f**, Population-averaged normalized SUA of sSC neurons with receptive fields smaller than 20° under three optogenetic/visual conditions. n=115 neurons, one-sided Wilcoxon signed-rank test, p=2.7E-20 for static versus moving gratings with LED off, p=2.1E-06 for LED off versus LED on with moving gratings. **g**-**i**, Silencing inhibitory NOT neurons relieves the suppression of flashed-evoked sSC activity when background motion extends into large (>20°) sSC receptive fields. Data presented as in **d**-**f**. n=55 neurons. One-sided Wilcoxon signed-rank test, p=5.7E-11 for **h**, p=3.2E-10 for static versus moving gratings with LED off in **i**, p=0.015 for LED off versus LED on with moving gratings in **i**. *, p<0.02; ***, p<0.001. Traces and summary data shown as mean±s.e.m.

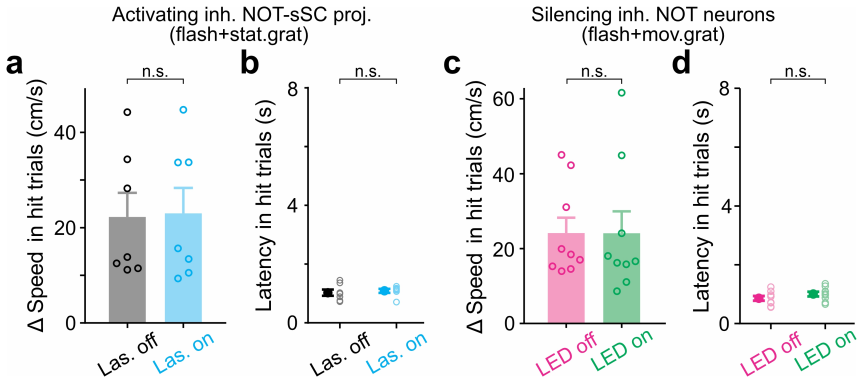

Supplementary Figure 13 | Optogenetic perturbation of NOT-sSC projections does not affect locomotor ability during behavioural performance. a,**b**, Summary of increment of mean running speed during black flashes (**a**) and behavioural onset latency (**b**) in hit trials during experiments of activating inhibitory (inh.) NOT-sSC projections when flashes are presented with static gratings (proj., n=7 mice, two-sided Wilcoxon signed-rank test, p=0.69 for **a** and **b**). **c**,**d**, Summary of increment of mean running speed during black flashes (**c**) and behavioural onset latency (**d**) in hit trials during experiments of silencing inhibitory NOT neurons when flashes are presented with moving gratings (n=9 mice, two-sided Wilcoxon signed-rank test, p=0.25 for **c** and **d**). Laser off and LED off, control trials; Laser on and LED on, optogenetics trials. n.s., not significant. Data shown as mean±s.e.m.
