# Supplementary material for: An inhibitory brainstem pathway reduces visual detection during background motion
Source: Nat Commun. 2026 May 7;17:6168. doi: 10.1038/s41467-026-72619-x (PMC13365429; doi:10.1038/s41467-026-72619-x)
Supplement: Supplementary file 2 — Description of Additional Supplementary Files [file 41467_2026_72619_MOESM2_ESM.pdf]

Description of Additional Supplementary Files:

Supplementary Movie 1. Example GO trials with static background gratings.

Supplementary Movie 2. Example NO-GO trials with static background gratings.

Supplementary Movie 3. Example GO trials with moving background gratings.

Supplementary Movie 4. Example NO-GO trials with moving background gratings.
