# Supplementary material for: An inhibitory brainstem pathway reduces visual detection during background motion
Source: Nat Commun. 2026 May 7;17:6168. doi: 10.1038/s41467-026-72619-x (PMC13365429; doi:10.1038/s41467-026-72619-x)
Supplement: Supplementary file 8 — Transparent Peer Review file [file 41467_2026_72619_MOESM8_ESM.pdf]

nature portfolio

Peer Review File

An Inhibitory Brainstem Pathway Reduces Visual Detection During Background Motion

Corresponding Author: Dr Bao-hua Liu

This file contains all reviewer reports in order by version, followed by all author rebuttals in order by version.

Version 0:

Reviewer comments:

Reviewer #1

(Remarks to the Author)
This is a very elegant study that reveals a new and exciting function of the accessory optic system. According to our current understanding, the accessory optic system is an evolutionary ancient ensemble of midbrain nuclei responsible for the stabilization of the image on the retina. In this experimental tour the force, Chou et al. clearly show that the nucleus of the optic tract (NOT; one of the main nuclei of the accessory optic system) is also involved in the contextual modulation of visual perception and of visual responses in the superior colliculus (SC). The underlying mechanisms relies on the inhibitory projections originating in the NOT and targeting the superficial (visual) layers of the SC (sSC). sSC neurons respond to small visual stimuli, like the appearance of a dark spot in the visual field. In contrast, NOT neurons respond to large (full field) moving stimuli. When a small stimulus, like a dark dot, appears on a moving background, the response of the SC to the dark dot is suppressed by the activation of inhibitory projections from the NOT. This suppression also impairs the perception of the dot. The experiments are well performed, and the data are clear. The fact that the accessory optic system is involved in visual perception and contextual modulation is extremely interesting and novel. I have several questions and comments that I would like the authors to address.

1. Why did the authors choose such a strong change in luminance as a stimulus (black dot)? The response in the SC to a milder change in luminance (dark gray) stimulus may have shown stronger inhibition by the moving background as well as stronger perceptual suppression. A psychometric function relating the change in luminance to the behavior and the impact of a moving background on that psychometric function could have helped finding the optimal change in luminance (i.e. the change in luminance level that is perceptually most impacted by the moving background).

2. It is not clear how the receptive field of individual SC neurons was mapped. In the methods (page 22 line 44) the RF mapping relates to multiunit. Accordingly, the terms classical and non-classical RFs for individual neurons are vague. It is well established that some visually responsive SC neurons have very large RFs (e.g. wide field cells) and yet preferentially respond to very small stimuli presented within that large RF. Thus, for such neurons both the background and the stimulus used in the present study may well be within the classical RF. In other words, while in the visual cortex literature the “background” is outside of the classical RF, in the SC this is not necessarily the case. This possibility does not reduce the value of the study but needs to be considered.

3. The SC and the NOT are responsible for eye movements in opposite directions. Activation of the left SC, for example, triggers a clockwise saccade. In contrast, the activation of the left NOT by a temporo-nasal stimulus seen by the right eye triggers a counterclockwise eye movement. In other words, image stabilization versus stimulus evoked gaze shifts represent opposing forces. The inhibitory projection of the NOT to the ipsilateral SC may in part serve to reduce stimulus triggered saccades by the SC when image stabilization requires eye movements in the opposite direction. The author should discuss this as it may represent the ancestral function of the inhibitory NOT-SC projection.

4. General narrative: Given the response of NOT neurons projecting to the SC (data in figure 3) the reader expects that the suppression of flash responses in sSC may be stronger in the presence of (a) larger moving backgrounds and (b) backgrounds moving in the temporo-nasal direction. This is indeed the case as shown, much later, in figure 5F and J. However, as presented, these important properties of the background in suppressing the SC response are not sufficiently highlighted. The authors should consider presenting the specificity of the background in suppressing SC responses as a separate figure immediately following figure 3 (e.g. a new fig. 4). This figure should be independent of the optogenetic

inactivation of NOT. In a subsequent figure (e.g. new figure 5) the authors can then show that the effect of NOT inactivation is specific to large and temporo-nasal backgrounds.

5. Page 7 lines 28- 30: Extended data 5 show inhibitory projection from NOT to vLGN. It is known that the vLGN sends inhibitory projections to the SC. If so, how can activity in NOT inhibit SC neurons via the vLGN?

Reviewer #2

(Remarks to the Author)
This study investigates the role of superior colliculus (SC) and its inputs from the nucleus of the optic tract (NOT) for the detection of local luminance changes in the presence of background motion in mice. This problem is addressed with a tour-de-force of experiments, including luminance change detection GO-NOGO task, single unit extracellular recordings, circuit tracing and optogenetics.
The findings show that:
• Mice can be trained to detect and report a change in luminance of a stationary target on a moving background. Perceptual sensitivity to these changes decreases in the presence of temporo-nasal background motion.
• In the mouse, the SC seems necessary to detect these local luminance changes; behaviour is supported by neural responses in the SC, which are increasingly suppressed by motion of backgrounds of increasing sizes.
• The suppression of activity in the SC depends on size tuned, functionally specific inhibitory projections from the NOT, which are increasingly activated by moving backgrounds of increase size. Inactivation of these projections rescues perceptual performance.

Overall, the study is timely and addresses the important question of the roles of the midbrain and brainstem circuits for visual perception, a role which is now on the central stage in mouse neuroscience and have been long neglected by cortico-centric studies. It demonstrate a perceptual relevance for a pathway, via the NOT, that has previously mostly been associated with the optokinetic reflex.

The experiments are well executed (bar my comments on the behavioural paradigm below), data clearly presented and analysed, and the findings intriguing for the field of visual neuroscience. However, there appear to be a fundamental problem left unnoticed, related to the rationale of the study, and the interpretation of the results: why would the brain implement a specific pathway to WORSEN perceptual capabilities? What is the MAIN purpose of this pathway? The explanation offered falls somehow short of satisfactory. This and other comments, which should be addressed before publication, are detailed below.

MAJOR COMMENTS
The study reports a surprising finding: in mice, a specific visual pathway exists, connecting NOT and SC, that worsens the ability to perceive changes of luminance of a target in the presence of temporo-nasal background motion. Why would the brain spend resources to build something so specific yet detrimental? It is concluded that the main function of this pathway must relate to gaze shifts, when self-generated visual flow needs to be cancelled out. However, gaze shifts are not analyzed, if anything explicitly avoided in the experimental design. Also, it is not clear why this suppression should be specific to the temporo-nasal direction, when gaze shifts occur in both directions. Does any evidence for these claims exist in the data that have been already collected? Even though the experimental design excludes consistent eye movements, certainly mice moved their eye during the task or passive recordings. Can these events be analysed to show how SC neurons responded, and how their responses changed during NOT inhibition?

Alternatively, perhaps this tension could be resolved explaining better the rationale motivating the study. The introduction broadly refers to ‘perceptual modulation’, but it does not explaine if this modulation consists in an increase or decrease in responses/perception, and what differences are thought to depend on which pathways. For example: are the findings relevant to the topic of motion induced blindness? The manuscript would greatly benefit for more priming and focus on the relevant background and hypotheses.

A related concern is whether the choice of the behavioral paradigm was appropriate for the problem under investigation. Is a change in local luminance in a stationary window analogous to the recognition of an object, or to foreground/background segmentation? Why would a head fixed task, with stationary eyes, be appropriate to study the function of a circuit chiefly involved in head and eye movements? In addition, the structure of the task needs to be clarified. What paces the task structure? Is the luminance change presented only when the mouse is stationary? Mice typically like to run for several minutes. What speed is considered as the threshold for the response? How does the animal know that a no-go trial has started?

MINOR COMMENTS

Starting from the abstract, and throughout the paper, the word ‘modulation’ is used to interchangeably mean ‘enhancement’ or ‘impairment’. Please clearly state the nature of the effects under discussion.

Please justify why a punishment training paradigm was required to train this task. The field has largely pivoted to using positive reinforcement not only for animal welfare reasons but also for reliability of results.

Please define in the main text what statistic is used to calculate the d prime for the task.
The frequent use of the word “innate”, clearly a ‘loaded word’ these days, is odd and vague: ‘’innate response” “innate circuit” “innate eye movements” “innate motor control” sounds undefined, or vague enough, that it is difficult to discern the intended meaning. Either be specific, or remove.

The temporo-nasal selectivity of the background movement is tested on neural but not behavioral recordings. It is also unclear why said temporo-nasal direction selectivity of the NOT response would matter for saccadic suppression OKR or head motion, since all of these can occur in any direction, not only temporo-nasally. Please explain or remove these claims.

Lines 16-17 on page 10: please clarify that only the inhibitory NOT-SC neurons are silenced, rather than writing “we optogenetically silenced inhibitory NOT neurons”.

Line 6 page 11: please remove the generalization to human perception, where the retinal composition and projections are very different. Use the word ‘may’ if you refer to mechanisms in other mammals.

Line 7 page 11: Which literature does the ‘dichotomous view’ under discussion comes from? In the mouse, the vast majority of the retinal projections go to the SC, which is the dominant structure for visuo-motor integration.

Some figures have minor errors such as words cut off, or resizing issues.

Version 1:

Reviewer comments:

Reviewer #1

(Remarks to the Author)
The authors have done a great job in addressing my comments and concerns. I am looking forward to seeing the manuscript in print.

Reviewer #2

(Remarks to the Author)
In the revised submission, the authors have substantially improved the manuscript by providing new analyses and edits, and —admirably—new experiments. These additions meaningfully clarify: the rationale for the study, the structure and design of the task, the contrast dependence of the results, the relevance of the described pathway for gaze shifts and MOB.
Most of our previous concerns have been carefully addressed. A few minor issues remain:
• Conflation of surrounding (context) shifts and gaze shifts in the introduction. Lines 4–5: 'A prime example of this phenomenon is the perceptual effects of moving backgrounds, which naturally occur when the head, eyes or surroundings shift'. While surrounding shifts (e.g. looking our from a train) cause movement of the background and not the foreground, gaze shifts (e.g. saccades) cause global motion of the visual scene. Please rephrase this sentence to make clear that the type of motion involved is not the same in the two cases, and thus the perceptual mechanisms may differ.
• Consider rephrasing the title. The phrase “in motional context” is ambiguous— it could refer either to the observer motion or the visual scene motion. In addition, “impairs” may overstate the effect. A rephrasing such as “An inhibitory brainstem pathway reduces visual detection during background motion” may improve clarity, searchability, and overall impact.
• Units for firing rate: firing rate should be reported in spikes/s rather than Hz. Please adjust throughout the manuscript.
• Direction-specific suppression of SC neurons: the data clearly show that NOT suppression of SC responses is specific to temporo–nasal motion. If available (without requiring new experiments), it would be informative to examine whether this inhibition preferentially targets SC neurons tuned to the temporo–nasal direction as well.

Reviewer #3

(Remarks to the Author)
I co-reviewed this manuscript with one of the reviewers who provided the listed reports. This is part of the Nature Communications initiative to facilitate training in peer review and to provide appropriate recognition for Early Career Researchers who co-review manuscripts.

Open Access This Peer Review File is licensed under a Creative Commons Attribution 4.0 International License, which permits use, sharing, adaptation, distribution and reproduction in any medium or format, as long as you give appropriate credit to the original author(s) and the source, provide a link to the Creative Commons license, and indicate if changes were made.
In cases where reviewers are anonymous, credit should be given to 'Anonymous Referee' and the source.
The images or other third party material in this Peer Review File are included in the article's Creative Commons license, unless indicated otherwise in a credit line to the material. If material is not included in the article's Creative Commons license and your intended use is not permitted by statutory regulation or exceeds the permitted use, you will need to obtain permission directly from the copyright holder.
To view a copy of this license, visit https://creativecommons.org/licenses/by/4.0/

To all reviewers:

We would like to thank the reviewers for their helpful comments on the manuscript. We made major revisions of the manuscript in the following aspects:

1. We examined the contrast psychometric function of flash detection to find the contrast where background motion has the strongest impact.
2. We performed new electrophysiological recordings with mapping RF of individual sSC neurons and examined whether inhibitory NOT neurons suppress the activity of sSC neurons with large or small RFs. We integrated this data into relevant figures.
3. We added the idea that inhibitory NOT-sSC projections could suppress orienting behaviour mediated by the SC for better image stabilization in the DISCUSSION section.
4. We removed the word "classical/non-classical RFs" and re-defined the "background gratings" as they are presented outside the flash stimuli, and but not outside classical RFs.
5. We presented the size and direction specificity of motion-induced suppression of SC activity in new Figure 4. The contribution of inhibitory NOT-sSC projections is presented in new Figure 5. Related Extended Data Figures are restructured to fit this change.
6. We modified the schematic in new Extended Data Figure 10e showing the possible polysynaptic relay from NOT to sSC and changed the manuscript accordingly.
7. We removed the DISCUSSION paragraph about the possible etiological function of the inhibitory NOT-sSC pathway. To replace it, we wrote a paragraph in which we propose that motion-induced perceptual impairment may facilitate global motion processing.
8. We clarified the criterion of a behavioural response in the Methods section and added the formula for d prime in the RESULTS.
9. We clarified the meaning of modulation throughout the manuscript by defining the direction of the change where appropriate.
10. We replaced the word "innate" throughout the manuscript to reflect the intended meaning.
11. We examined the dependence of the contribution of inhibitory NOT-sSC projections to visual detection on motion direction.
12. We revised the first sentence in the DISCUSSION paragraph about the dichotomous view of global motion processing systems.
13. We revised the manuscript (highlighted in green) and modified figures to address reviewers' comments and to integrate new data into the manuscript.

Here are our replies to reviewers' comments.

Reviewer #1 (Remarks to the Author):

This is a very elegant study that reveals a new and exciting function of the accessory optic system. According to our current understanding, the accessory optic system is an evolutionary ancient ensemble of midbrain nuclei responsible for the stabilization of the image on the retina. In this experimental tour the force, Chou et al. clearly show that the nucleus of the optic tract (NOT; one of the main nuclei of the accessory optic system) is also involved in the contextual modulation of visual perception and of visual responses in the superior colliculus (SC). The underlying mechanisms relies on the inhibitory projections originating in the NOT and targeting the superficial (visual) layers of the SC (sSC). sSC neurons respond to small visual stimuli, like the appearance of a dark spot in the visual

field. In contrast, NOT neurons respond to large (full field) moving stimuli. When a small stimulus, like a dark dot, appears on a moving background, the response of the SC to the dark dot is suppressed by the activation of inhibitory projections from the NOT. This suppression also impairs the perception of the dot. The experiments are well performed, and the data are clear. The fact that the accessory optic system is involved in visual perception and contextual modulation is extremely interesting and novel. I have several questions and comments that I would like the authors to address.

We sincerely thank the reviewer for the constructive comments, as well as for the overall positive evaluation of our work.

Specific comments:

1. Why did the authors choose such a strong change in luminance as a stimulus (black dot)? The response in the SC to a milder change in luminance (dark gray) stimulus may have shown stronger inhibition by the moving background as well as stronger perceptual suppression. A psychometric function relating the change in luminance to the behavior and the impact of a moving background on that psychometric function could have helped finding the optimal change in luminance (i.e. the change in luminance level that is perceptually most impacted by the moving background).

We thank the reviewer for raising an important question of whether the suppressive effects of background motion on flash detection depend on the strength, namely the contrast, of flash stimuli. To answer this question, we examined the contrast psychometric function by systematically varying the luminance change of the central circular patch surrounded by static or moving gratings (100% contrast = Δ luminance from 40cd/m^2 to 0cd/m^2; 50% contrast = Δ luminance from 40cd/m^2 to 20cd/m^2). We found that the animals' task performance is very sensitive to the contrast. While at 100% contrast animals achieved a hit rate of 0.84, at a moderate-to-high contrast of 64% the hit rate decreased dramatically to only 0.47. Importantly, the background motion impaired the task performance in a contrast-dependent manner, producing the largest reduction in hit rate (Δ hit rate = -0.233) at 100% contrast and a much weaker effect (Δ hit rate = -0.078) at 64% contrast. Therefore, the detection of high-contrast flashes is more strongly impacted by moving backgrounds than that of moderate- or low-contrast flashes, which supports our choice of 100%-contrast flashes as target stimuli. We have included the details of this experiment in the revised manuscript (**Extended Data Fig. 1m; page 4, lines 6-12; page 23, lines 43-45; page 28, lines 21-25**).

2. It is not clear how the receptive field of individual SC neurons was mapped. In the methods (page 22 line 44) the RF mapping relates to multiunit. Accordingly, the terms classical and non-classical RFs for individual neurons are vague. It is well established that some visually responsive SC neurons have very large RFs (e.g. wide field cells) and yet preferentially respond to very small stimuli presented within that large RF. Thus, for such neurons both the background and the stimulus used in the present study may well be within the classical RF. In other words, while in the visual cortex literature the "background" is outside of the classical RF, in the SC this is not necessarily the case. This possibility does not reduce the value of the study but needs to be considered.

The reviewer correctly noted that the receptive fields (RFs) of sSC neurons exhibit substantial variability in size and that some are larger than the local flashes used in this study (20°). In this study, we estimated the RF centers of sSC neurons with multi-unit activity, but not with single-unit activity (**page 4, lines 29-30**), because the repetition number of visual stimulation (10) was too low to

reconstruct the receptive fields of most sSC neurons based on single-unit activity. Therefore, it is possible that in the dataset of our first submission some sSC neurons had large RFs that were partially overlaid by the background gratings. This possibility raises a question of whether the suppressive effect of background motion depends on the RF size of SC neurons. To address this question, we performed new recordings in which we mapped the RFs of individual sSC neurons based on single-unit activity evoked by doubled repetition number (20), before examining the effect of silencing inhibitory NOT neurons on the motion modulation of sSC activity. We found that among sSC neurons that are responsive to central flashes, ~30% had RFs larger than 20° (**Extended Data Fig. 12c**). Based on the relationship between RFs and background gratings, we divided sSC neurons into two groups: sSC neurons with receptive fields smaller than 20°, which were surrounded by the background gratings; sSC neurons with receptive fields larger than 20°, which were partially overlapped by the background gratings (**Extended Data Fig. 12d,g, schematics**). In both groups, background gratings, although not activating sSC neurons, strongly suppressed flash-evoked sSC activity (**Extended Data Fig. 12d-i**). Importantly, in both groups silencing inhibitory NOT neurons attenuated the suppressive effect of background motion (**Extended Data Fig. 12f,i**). Therefore, these results demonstrate that inhibitory NOT-sSC projections suppress the activities of sSC neurons, even when background motion is present within their RFs. We have added this experiment in the revised manuscript (**Extended Data Fig. 12**; **page 10, lines 20-31; page 11, lines1-2; page 25, lines10-15; page 29, lines25-38**).

Furthermore, since some large sSC RFs were partially covered by the background gratings, the terms "classical RF" and "non-classical RF" are not accurate in interpreting the circuit mechanism behind this study. Thus, we removed them in the revised manuscript. Accordingly, we re-defined the "background gratings" as they are presented to the region surrounding the flash stimuli that are centered on the RF of sSC neurons, and they don't need to be exclusively outside the classical RF. These changes can be found at several places in the new manuscript (**page 9, lines 27-29; page 16, line 20; page 20, line 22**).

3. The SC and the NOT are responsible for eye movements in opposite directions. Activation of the left SC, for example, triggers a clockwise saccade. In contrast, the activation of the left NOT by a temporo-nasal stimulus seen by the right eye triggers a counterclockwise eye movement. In other words, image stabilization versus stimulus evoked gaze shifts represent opposing forces. The inhibitory projection of the NOT to the ipsilateral SC may in part serve to reduce stimulus triggered saccades by the SC when image stabilization requires eye movements in the opposite direction. The author should discuss this as it may represent the ancestral function of the inhibitory NOT-SC projection.

The reviewer raised a good point. As the reviewer pointed out, the NOT is primarily driven by temporo-nasal motion presented to the contralateral eye, making the eye move in the temporo-nasal direction (ipsiversive) in OKR, while the activation of the SC on the same side by visual stimulation presented to the contralateral eye leads to naso-temporal eye movement (contraversive) in orienting behaviour. Thus, in theory the inhibitory projections from the NOT to SC could suppress orienting behaviour for better image stabilization. We have added this point in the DISCUSSION of the revised manuscript (**page 15, lines 6-10**).

4. General narrative: Given the response of NOT neurons projecting to the SC (data in figure 3) the reader expects that the suppression of flash responses in sSC may be stronger in the presence of (a) larger moving backgrounds and (b) backgrounds moving in the temporo-nasal direction. This is indeed

the case as shown, much later, in figure 5F and J. However, as presented, these important properties of the background in suppressing the SC response are not sufficiently highlighted. The authors should consider presenting the specificity of the background in suppressing SC responses as a separate figure immediately following figure 3 (e.g. a new fig. 4). This figure should be independent of the optogenetic inactivation of NOT. In a subsequent figure (e.g. new figure 5) the authors can then show that the effect of NOT inactivation is specific to large and temporo-nasal backgrounds.

We thank the reviewer for the great suggestion and have reorganized figure panels accordingly. We put panels showing the size- and direction-dependence of motion-induced suppression of sSC activity in **new Figure 4**. The description of this figure was also singled out in the RESULTS (**page 7, lines 23-32; page 8, lines 1-8**). Moreover, the panels related to eOPN3 experiments in **old Figure 4** merged with other eOPN3-related panels in **old Extended Data Figures 9&10** and became **new Extended Data Figures 10**. And the remaining panels in **old Figures 4&5** were combined into **new Figure 5**, which focuses on the size- and direction-dependent contribution of inhibitory NOT-sSC projections to motion-induced suppression of sSC activity. We also reorganized the panels in **new Extended Data Figures 8-11**, which are relevant to **new Figure 5**. Correspondingly, **new Figure 5** was described in one section (**from page 8, line 9 to page 11, line 2**). This new plan of figure presentation can effectively highlight the properties of the background motion in suppressing sSC activity and work better to logically transit from the response properties of inhibitory NOT-sSC projections in **Figure 3** to their contribution to background motion-exerted suppression of sSC activity in **Figure 5**.

5. Page 7 lines 28- 30: Extended data 5 show inhibitory projection from NOT to vLGN. It is known that the vLGN sends inhibitory projections to the SC. If so, how can activity in NOT inhibit SC neurons via the vLGN?

We agree with the reviewer that vLGN sends primarily inhibitory projections to the SC. In principle, the NOT-vLGN-SC pathway should exert a disinhibitory effect, leading to activation rather than inhibition of sSC activity. However, it is still possible that inhibitory NOT projections to non-SC nuclei may contribute to the motion-induced suppression of SC activity. For example, inhibitory NOT neurons project to the dLGN, which can provide disynaptic excitation to the SC via the V1, (see schematic below). The polysynaptic relay of the NOT-dLGN-V1-SC pathway would in theory allow inhibitory NOT neurons to exert a suppressive effect on SC activity. We modified the schematic in **new Extended Data Figure 10e** and revised the manuscript accordingly (**page 8, lines 26-28**) to accurately reflect this possibility.

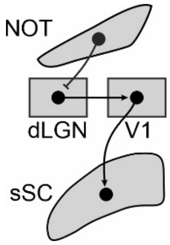

The polysynaptic relay of the NOT-dLGN-V1-SC pathway

Reviewer #2 (Remarks to the Author):

This study investigates the role of superior colliculus (SC) and its inputs from the nucleus of the optic tract (NOT) for the detection of local luminance changes in the presence of background motion in mice. This problem is addressed with a tour-de-force of experiments, including luminance change detection

GO-NOGO task, single unit extracellular recordings, circuit tracing and optogenetics.

The findings show that:

• Mice can be trained to detect and report a change in luminance of a stationary target on a moving background. Perceptual sensitivity to these changes decreases in the presence of temporo-nasal background motion.

• In the mouse, the SC seems necessary to detect these local luminance changes; behaviour is supported by neural responses in the SC, which are increasingly suppressed by motion of backgrounds of increasing sizes.

• The suppression of activity in the SC depends on size tuned, functionally specific inhibitory projections from the NOT, which are increasingly activated by moving backgrounds of increase size. Inactivation of these projections rescues perceptual performance.

Overall, the study is timely and addresses the important question of the roles of the midbrain and brainstem circuits for visual perception, a role which is now on the central stage in mouse neuroscience and have been long neglected by cortico-centric studies. It demonstrate a perceptual relevance for a pathway, via the NOT, that has previously mostly been associated with the optokinetic reflex.

The experiments are well executed (bar my comments on the behavioural paradigm below), data clearly presented and analysed, and the findings intriguing for the field of visual neuroscience. However, there appear to be a fundamental problem left unnoticed, related to the rationale of the study, and the interpretation of the results: why would the brain implement a specific pathway to WORSEN perceptual capabilities? What is the MAIN purpose of this pathway? The explanation offered falls somehow short of satisfactory. This and other comments, which should be addressed before publication, are detailed below.

We sincerely thank the reviewer for the positive assessment and constructive suggestions. We agree that it is important to discuss potential purposes or ethological functions of this inhibitory pathway, since it has a negative impact on perception—impairing the perception of local flashes. Therefore, we attempted to interpret the meaning of our findings using gaze shifts as an example in the DISCUSSION of previous submission (**page 12, lines 20-31**). Given the robustness of behavioural and physiological phenotypes in this study, we believe that the circuit mechanism we revealed may function in all scenarios when temporo-nasal background motion is seen, including but not limited to gaze shifts cause by head, body or eye movements. Nevertheless, considering the concerns and suggestions raised in points 1-3, and inspired by studies on motion-induced blindness, we rewrote this paragraph from the perspective of the competition between global motion processing and local static processing (**page 14, lines 1-20**). Please see details below in responses to points 1-3.

MAJOR COMMENTS

1. The study reports a surprising finding: in mice, a specific visual pathway exists, connecting NOT and SC, that worsens the ability to perceive changes of luminance of a target in the presence of temporo-nasal background motion. Why would the brain spend resources to build something so specific yet detrimental? It is concluded that the main function of this pathway must relate to gaze shifts, when self-generated visual flow needs to be cancelled out. However, gaze shifts are not analyzed, if anything explicitly avoided in the experimental design. Also, it is not clear why this suppression

should be specific to the temporo-nasal direction, when gaze shifts occur in both directions. Does any evidence for these claims exist in the data that have been already collected? Even though the experimental design excludes consistent eye movements, certainly mice moved their eye during the task or passive recordings. Can these events be analysedw to show how SC neurons responded, and how their responses changed during NOT inhibition?

We thank the reviewer for the comments and apologize for the confusion caused by the paragraph in the previous manuscript discussing the potential purpose or ethological function of this inhibitory pathway. In this DISCUSSION paragraph, we aimed to propose, but not to conclude, a possible naturalistic scenario when the mechanism revealed by this study may contribute to the vision in a positive way (**page 12, lines 20-31** in previous submission). We did so by saying "this inhibitory pathway **may** suppress visual processing during gaze shifts" and "subcortical projections broadcasting signals of temporo-nasal global motion **likely** contribute to vision only during rotational movements". This proposal was inspired by three findings and existing ideas. 1) Inhibitory NOT-sSC projections respond to temporo-nasal background motion and suppress sSC activity in such context (**Fig. 3&5**); 2) one well-known scenario linked to temporo-nasal background motion is the gaze shift cause by head, body or eye movements (in the form of saccades)(*Warren 1995; Harris 1994*); 3) during saccadic gaze shifts, visual sensitivity is transiently suppressed, preventing the brain from perceiving the image blur or motion generated by eye movement and thereby contributing to the perception of a stable visual world (*Masson and Ilg 2010; Krekelberg 2010; Thiele et al. 2002*). The basic idea behind our proposal is that self-generated visual flow caused by gaze shifts may engage inhibitory NOT-sSC projections to suppress visual processing, which prevents the brain from perceiving blurred images caused by gaze shifts and thus facilitates the transition from one scene to another.

Moreover, we did not imply that the main function of this pathway **MUST** relate to gaze shifts. In the DISCUSSION of previous submission, we compared two basic types of self movements, rotational (gaze shifts) or translational movements (running), as examples of when global motion occurs. In that comparison, we proposed that inhibitory NOT projections may be recruited to suppress visual processing during rotational but not translational movements. Nevertheless, we think that inhibitory NOT projections can be recruited in other scenarios of temporo-nasal background motion, such as environmental motion in nature (ripples in the water, waving tree branches in the forest, etc.), since they are very sensitive to moving background gratings (**Fig. 3**).

As gaze shifts are unnecessary for the mechanism under investigation, and may introduce complications such as image blur, misalignment between visual stimuli with receptive fields, and changes in brain states, we intentionally used head-fixed preparation to avoid head and eye movements. We also excluded from analysis all trials in which eye movements were detected (also see our responses to point 3).

Regarding the temporo-nasal direction bias (also see our responses to point 9), it is worth noting that background motion in all tested directions strongly suppresses both sSC activity (**Fig. 4f**) and visual detection (**Fig. 6i-l**). However, only the contribution from inhibitory NOT-sSC projections exhibit a pronounced temporo-nasal bias (**Fig. 5k-m**,**6i-l**). Thus, we proposed that the contribution of this pathway to visual processing during gaze shifts may be specific to temporo-nasal background motion.

Following reviewer's suggestion, we analyzed our data for evidence supporting our original proposal. We searched for trials in which the right eye moved naso-temporally during flash stimulation, which

could cause temporo-nasal background motion on the retina (note that recording was done from left SC, which receives inputs primarily from the right eye). Unfortunately, we found on average only ~0.5 such trials out of 20 repetition trials of each stimulation condition in one recording session (see figure below), which is consistent with the reports that head-fixed mice barely move eyes (*Meyer et al. 2020*). Such low samples size prevents us from assessing how SC neurons responded to gaze shifts, and how their responses changed during NOT inhibition. To address these two questions, recording from freely moving mice are needed, which is an interesting project for the future.

References:

Harris. in Visual detection of motion: Visual motion caused by movements of the eye, head and body Vol.1 Ch.14, 397-435 (Academic Press, 1994).

Krekelberg. Saccadic suppression. Current Biology 20 (2010):R228-R229.

Masson and Ilg. Dynamics of visual motion processing. New-York: Springer-Verlag, 2010.

Meyer et al. Two distinct types of eye-head coupling in freely moving mice. Curr Biology 30, (2020):2116-30.

Thiele et al. Neural mechanisms of saccadic suppression. Science 295 (2002):2460-2462.

Warren. in Perception of Space and Motion: Self-Motion: Visual Perception and Visual Control (eds W. Epstein Rogers & J. S) Ch.8, 263-325 (Academic Press, 1995).

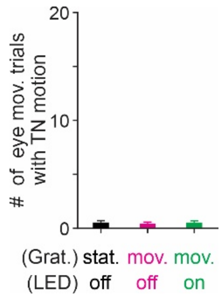

Number of recording trials with naso-temporal eye movements generating temporo-nasal (TN) background motion

2. Alternatively, perhaps this tension could be resolved explaining better the rationale motivating the study. The introduction broadly refers to 'perceptual modulation', but it does not explain if this modulation consists in an increase or decrease in responses/perception, and what differences are thought to depend on which pathways. For example: are the findings relevant to the topic of motion induced blindness? The manuscript would greatly benefit for more priming and focus on the relevant background and hypotheses.

The reviewer's comments in point 1 made us realize that framing the discussion from the perspective of gaze shifts (eye/head/body movements) could potentially mislead readers. Accordingly, we revised this paragraph from the angle of motion-induced blindness (MIB), as the reviewer suggested. Phenotypically, MIB in humans closely resembles the motion-induced impairment in flash detection observed in our paradigm, as both involve the loss of saliency of static objects in the presence of background motion. Inspired by studies on MIB, we propose that the impairment in flash detection in our paradigm may reflect a competition in which global motion processing dominate over local static processing. The circuits identified in this study may provide a neural substrate for such competition. First, in the brainstem, global motion and local flashes are processed by distinct structures: NOT neurons preferentially respond to global motion (**Fig. 3**), whereas sSC neurons favor local flashes (**Fig. 1k,m, Extended Data Fig. 3b,3f,12e,12h**). Second, the NOT circuit that processes global motion is directly connected to the sSC circuit processing local flashes via inhibitory projections (**Fig. 2**). Third, these projections convey global motion signals to the sSC (**Fig. 3c-h**), where they suppress local flash

processing (**Fig. 5&6**). We have incorporated these ideas into the revised DISCUSSION section of the manuscript (**page 14, lines 1-20**).

We thank the reviewer for their suggestion to refine the rationale of our study in the INTRODUCTION. However, we did not restructure the INTRODUCTION to focus exclusively on decreases in responses/perception, nor frame the study in the context of MIB, for three reasons. First, we believe that the tension raised in point 1 is largely resolved by discussing the potential function of the inhibitory NOT-sSC pathway from the perspective of MIB (see above paragraph). Second, since MIB is a human perceptual phenomenon, priming the INTRODUCTION with this could unintentionally imply that mice were used to model MIB and/or identify its neural substrates. This would be misleading, as we currently have no evidence that mouse phenotype observed in this study is equivalent to MIB. Third, background motion can either increase or decrease responses/perception, depending on the visual features of both local stimulus and surrounding motion (e.g., contrast, motion direction, and speed). Since, to our knowledge, no prior study has used our visual stimulation paradigm combining local static flashes with surrounding moving gratings, we couldn't predict whether such background motion would increase or decrease responses/perception evoked by local flashes. In addition, because the NOT sends both inhibitory and excitatory projections, it could theoretically either decrease or increase the activity of its downstream targets. For these reasons, reshaping the INTRODUCTION to focus solely on decrease in responses/perception would make the rationale biased and contrived. Instead, we aimed to provide a balanced and comprehensive overview in the INTRODUCTION, addressing both potential directions of modulation. Accordingly, we clearly stated one goal of this study as "assess whether background motion impairs or enhances subcortically driven visual perception" (**page 3, lines 4-6**). Nevertheless, we have defined the direction of modulation where appropriate by using terms such as "increasing" or "decreasing" (also see our responses to point 5).

Regarding the comment "what differences are thought to depend on which pathways", previous studies have not identified a clear distinction between the neural circuits meditating facilitatory versus suppressive effects of motion modulation. For example, both feedback inputs from higher visual areas and local cortical inhibition can increase or decrease cortical activity, depending on the visual features of the center and surround stimuli (*Keller et al. 2020; Fişek et al. 2023; Di Santo et al. 2025*).

References:

Di Santo et al. Contextual modulation emerges by integrating feedforward and feedback processing in mouse visual cortex. Cell Reports 44 (2025).

Fişek et al. Cortico-cortical feedback engages active dendrites in visual cortex. Nature 617 (2023):769-776.

Keller et al. A disinhibitory circuit for contextual modulation in primary visual cortex. Neuron 108 (2020):1181-1193.

3. A related concern is whether the choice of the behavioral paradigm was appropriate for the problem under investigation. Is a change in local luminance in a stationary window analogous to the recognition of an object, or to foreground/background segmentation? Why would a head fixed task, with stationary eyes, be appropriate to study the function of a circuit chiefly involved in head and eye movements?

We thank the reviewer for raising these important questions. Regarding the design of visual stimulation, the task used in this study is a detection task, in which mice simply report whether a local luminance change occurs. As in other detection tasks, we presented only a single stimulus with fixed visual

features. Similar visual stimulation has been used in previous studies investigating luminance-change detection (*Cone et al. 2020&2024; Glickfeld et al. 2013; Jin and Glickfeld 2020*). Our visual stimulation is particularly suitable for assessing the function of sSC circuits, as prior work has shown that the sSC mediates detection of luminance changes (*Cone et al. 2024, Cazemier et al. 2024*). Our task differs from discrimination or object recognition. Discrimination tasks typically involve two stimuli (target versus distractor) that differ in a specific visual feature such as contrast, direction, orientation (*Jin and Glickfeld 2020; Wong and Brown 2006*), whereas object recognition requires the integration of multiple visual features such as shapes, colors, and textures (*Kourtzi&Connor 2011; Di Carlo et al. 2012*). Similarly, our task differs from foreground/background (figure/ground) segmentation, which typically involves two texturally similar regions (figure and ground) differing in orientation, motion direction, colour, etc. of texture elements (*Caputo 1996; Lamme 1995; Roelfsema 2002*). To avoid misinterpretation, we deliberately refrained from using the term “foreground” in the manuscript. To further clarify terminology, we explicitly defined “background” in this study as a sinusoidal grating surrounding local flashes (**page 3, lines 25-26**).

We suspect that the impression that our flash detection task was intended as an analog of object recognition or foreground/background segmentation may have arisen from the first paragraph of the INTRODUCTION. Accordingly, we have revised it to prevent this misunderstanding (**page 1, line 10**).

Regarding the choice of head-fixed preparation, we intentionally did so to eliminate head and eye movements for the following reasons. First, we anticipated that the inhibitory NOT-sSC pathway would contribute to vision in any situation involving temporo-nasal global motion, such as environmental motion, regardless of head and eye movements. This expectation was based on previous findings showing that the NOT responds robustly to moving drum gratings even in absence of head or eye movements (*Hoffmann and Schoppmann 1981; Volchan et al. 1989; He et al. 2024*). Consistently, our results show that inhibitory NOT projections impact sSC activity and visual perception during background motion in head-fixed mice without eye movements (**Fig. 5&6, Extended Data Fig. 4a-e,6a-e,9a-e**). Second, while head and eye movements can produce global motion, they are also confounding factors that complicate data interpretation. They blur retinal images (*Gegenfurtner 2016; Bedell et al. 2010*), misalign visual stimuli relative to SC receptive fields, and increase variability in neuronal activity associated with brain states (*Johnston et al. 2022; Abdolrahmani et al. 2021*). To deal with these confounds, we took a reductionist approach by head-fixing mice, correcting stimulus position according to eye movements, and excluding from analysis trials in which the eye moved (**page 25, lines 45-47; page 26, lines 1-13**). This approach allowed us to isolate the contribution of neural circuits to sensory processing with minimal ambiguity. Nevertheless, we recognized that previous discussion in the context of gaze shifts (previous manuscript, **page 12, lines 20-31**; see our responses to point 1) may be confusing and therefore rewrote this paragraph from a different perspective (see our responses to point 2).

References:

Abdolrahmani et al. Attention separates sensory and motor signals in the mouse visual cortex. Cell reports 36 (2021):109377.

Bedell et al. The perception of motion smear during eye and head movements. Vision research 50 (2010):2692–2701.

Caputo. The role of the background: texture segregation and figure-ground segmentation. Vision research 36 (1996):2815–2826.

Cazemier et al. Involvement of superior colliculus in complex figure detection of mice. Elife 13 (2024): e83708.

Cone et al. Mice preferentially use increases in cerebral cortex spiking to detect changes in visual stimuli. J. of Neurosci. 40 (2020):7902-7920.

Cone et al. Stimulus-dependent differences in cortical versus subcortical contributions to visual detection in mice. Current Biology 34 (2024):1940-1952.

DiCarlo et al. How does the brain solve visual object recognition? Neuron, 73 (2012):415–434.

Gegenfurtner. The Interaction Between Vision and Eye Movements. Perception 45(2016):1333–1357.

Glickfeld et al. Mouse primary visual cortex is used to detect both orientation and contrast changes. J. of Neurosci. 33 (2013):19416-19422.

He et al. Brainstem inhibitory neurons enhance behavioral feature selectivity by sharpening the tuning of excitatory neurons. Current biology 34 (2024):4623–4638.e8.

Hoffmann and Schoppmann. A quantitative analysis of the direction-specific response of Neurons in the cat's nucleus of the optic tract. Experimental brain research 42 (1981):146–157.

Jin and Glickfeld. Mouse higher visual areas provide both distributed and specialized contributions to visually guided behaviors. Current Biology 30 (2020):4682-4692.

Johnston et al. The eyes reflect an internal cognitive state hidden in the population activity of cortical neurons. Cerebral cortex (2022):3331–3346.

Kourtzi and Connor. Neural representations for object perception: structure, category, and adaptive coding. Annual review of neuroscience (2011): 45–67.

Lamme. The neurophysiology of figure-ground segregation in primary visual cortex. The Journal of neuroscience 15 (1995):1605–1615.

Roelfsema. Figure-ground segregation in a recurrent network architecture. Journal of cognitive neuroscience 14 (2002):525–537.

Volchan et al. Visual response properties of pretectal units in the nucleus of the optic tract of the opossum. Experimental brain research: 78 (1989): 380–386.

Wong and Brown. Visual detection, pattern discrimination and visual acuity in 14 strains of mice. Genes, brain, and behavior 5 (2006): 389–403.

4. In addition, the structure of the task needs to be clarified. What paces the task structure? Is the luminance change presented only when the mouse is stationary? Mice typically like to run for several minutes. What speed is considered as the threshold for the response? How does the animal know that a no-go trial has started?

Task timing was controlled by custom software. Each trial included three phases: 1) a pre-flash/blank period ranging from 11-21 sec, determined by a uniform distribution, 2) a flash/blank period of 3 sec, and 3) a post-flash/blank period of 1 sec (**page 23, lines 32-34**). When the pre-flash/blank period elapsed, a flash appeared immediately, no matter whether the animal was running or not.

As the reviewer pointed out, occasionally mice run spontaneously for a couple of minutes. Our response criterion was defined as an increase in running speed of more than 10cm/s relative to the average speed during the 3 sec preceding flash onsets. In other words, if a mouse was already running when the flash appeared, it needed to run faster by at least 10cm/s to avoid an electrical shock. Otherwise, such a trial was classified as a miss, and an electric shock was delivered. Consequently, if a mouse maintained a constant running speed throughout a trial, such trial was not considered a hit trial. We have revised the METHODS to clarify response criterion (**page 24, lines 6-7**).

To evaluate the potential contribution of continuous running on behavioural analysis, we examined the number of trials in which mice run (>1 cm/s) continuously throughout the trial and found that such

trials were rare (on average 6.0/125 trials). Additionally, their contribution to d prime was minimal and did not change our conclusions (see figure below).

In the NO-GO trials of our paradigm, no additional sensory cue was provided at the start of each trial, so that mice couldn't anticipate trial onset (**page 24, lines 10-11**). We did so for the following reasons. First, in NO-GO trials of our detection task, there was no distractor, but instead blank stimulus (the absence of target stimulus—flashes). The purpose of NO-GO trials was to evaluate the probability that animals falsely respond to the blank stimulus (false alarm rate). Second, a cueing signal at the start of NO-GO trials is typically used to suppress false responses (*Leland et al. 2008; Roberts et al.2016*). Given the reasonably low false alarm rate in our results, this cueing signal was unnecessary. Third, previous studies have reported that cueing signals at the start of NO-GO trials can in some cases impair behaviour performance by introducing bias and increasing false alarm rates (*Jaffard et al. 2007*). Last, many prior studies have successfully applied behavioural paradigms without such cue in visual detection tasks (*Glickfeld et al. 2013; Cone et al. 2020, 2024; Jin&Glickfeld. 2020; Montij et al. 2015*).

References:

Coneb et al. Mice preferentially use increases in cerebral cortex spiking to detect changes in visual stimuli. J. of Neurosci. 40 (2020):7902-7920.

Cone et al. Stimulus-dependent differences in cortical versus subcortical contributions to visual detection in mice. Current Biology 34 (2024):1940-1952.

Glickfeld et al. Mouse primary visual cortex is used to detect both orientation and contrast changes. J. of Neurosci. 33 (2013):19416-19422.

Glickfeld. Mouse higher visual areas provide both distributed and specialized contributions to visually guided behaviors. Current Biology 30 (2020):4682-4692.

Jaffard et al. Cueing method biases in visual detection studies. Brain research 1179 (2007):106-118.Jin and

Montijn et al. Mouse V1 population correlates of visual detection rely on heterogeneity within neuronal response patterns. Elife 4 (2015):e10163.

Leland et al. "Anterior cingulate cortex and benefit of predictive cueing on response inhibition in stimulant dependent individuals." Biological psychiatry 63 (2008): 184-190.

Roberts et al. "The effects of preresponse cues on inhibitory control and response time in adults with ADHD." Journal of attention disorders 20 (2016): 317-324.

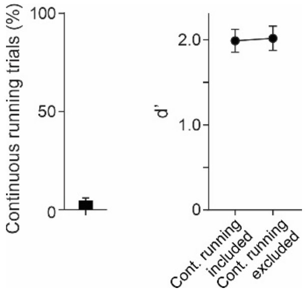

Continuous running rarely occurs in our behavioural paradigm nor affects the quantification of behavioural phenotype.

MINOR COMMENTS

5. Starting from the abstract, and throughout the paper, the word 'modulation' is used to interchangeably mean 'enhancement' or 'impairment'. Please clearly state the nature of the effects under discussion.

We thank the reviewer for the suggestion. In response to it, we replaced the words "modulation" and "modulate" with "impairment", "suppression", "impair", "suppress", "enhance" to define the nature of the effects, where appropriate. In RESULTS and DISCUSSION, we particularly made it clear that

background motion and inhibitory NOT-sSC projections suppress sSC activity and impair visual detection. Please find these changes in the manuscript (**page 1, lines 1&19; page 2, lines 15-20&25; page 3, line 6; page 5, lines 13&16; page 6, line 30; page 7, lines 22&25; page 8, line 21; page 9, line 17; page 10, lines 2&14&17; page 11, lines 16&17; page 12, line 27; page 14, lines 21&28&30; page 25, lines 32&38; page 29, line 15**).

6. Please justify why a punishment training paradigm was required to train this task. The field has largely pivoted to using positive reinforcement not only for animal welfare reasons but also for reliability of results.

We thank the reviewer for the comments. In studies of using mice as the model organism, punishment-based training paradigms have been widely used to understand the functions of brain circuits. In GO/NO-GO based detection tasks, punishments including tail/foot shocks have been successfully used to motivate mice to report the presence of sensory cues (*Makino and Komiyama 2015; Guo et al. 2017; Ma et al. 2021; Wong and Brown 2006*). In GO/NO-GO based discrimination tasks, punishments have often been associated with the distractors to promote the correct rejection of distractors (*Lutas et al. 2019; Yang et al. 2023; Zhu et al. 2018*). In fear conditioning tasks, aversive stimuli including foot shocks have been used as the unconditioned stimulus to create a fear association with a neutral conditioned stimulus (*Shanks et al. 2016; Letzkus et al. 2011; Grewe et al. 2017; Fadok et al. 2017*).

In this study, we chose this punishment-based training paradigm for three reasons. First, when conceptualizing this project based on our unexpected observation of profound inhibitory NOT-sSC projections, we sought a behavioural paradigm that critically depends on SC functions. The SC is well-known to play a central role in visually guided avoidance/escape behaviours for survival (*Hoy and Farrow 2025; Li et al., 2023; Yilmaz and Meister 2013; Shang et al., 2018*). Thus, we designed a training paradigm in which animals run to avoid stimuli associated punishment, mimicking this SC dependent behaviour (**page 24, lines 9-10**). Second, it has been reported that electric shocks are generally more effective for promoting learning than food- or water-based rewards. Tasks using electric shocks can typically be learned within a few days (*Shanks et al. 2016; Makino&Komiyama 2015; Grewe et al. 2017; Fadok et al. 2017; Lutas et al. 2019*), whereas water reward-based tasks often require several weeks of learning (*Cone et al. 2024; Peters et al. 2014; Li et al. 2020; Cheung et al. 2019; Lutas et al. 2019*). Consistently, mice in our study needed only 1-2 weeks to learn the task, and our paradigm consistently produced robust behavioural phenotypes across individuals, including motion-induced impairment in flash detection and reproducible effects of optogenetic perturbations (**Fig. 1f-i,6 Extended Data Fig. 2**). Third, the punishment used in our paradigm consisted of short and mild electrical shocks (0.2 sec, 0.3 mA), which have minimal impact on animal welfare compared with food or water restriction commonly used in reward-based training paradigms. Food or water restriction often results in substantial body weight loss and severe health complications.

References:

Cheung et al. The sensorimotor basis of whisker-guided anteroposterior object localization in head-fixed mice. Current Biology 29 (2019):3029-3040.

Fadok et al. A competitive inhibitory circuit for selection of active and passive fear responses. Nature 542 (2017):96-100.

Grewe et al. Neural ensemble dynamics underlying a long-term associative memory. Nature 543 (2017):670-675.

Guo et al. A corticothalamic circuit for dynamic switching between feature detection and discrimination. Neuron 95 (2017):180-194.

Hoy and Farrow. The superior colliculus. Current Biology 35 (2025):R164-R168.

Letzkus et al. A disinhibitory microcircuit for associative fear learning in the auditory cortex. Nature 480 (2011):331-335.

Li et al. Pathway-specific inputs to the superior colliculus support flexible responses to visual threat. Science Advances 9 (2023):eade3874.

Li et al. Inhibition of impulsive action by projection-defined prefrontal pyramidal neurons. PNAS 117 (2020):17278-17287.

Lutas et al. State-specific gating of salient cues by midbrain dopaminergic input to basal amygdala. Nature neurosci. 22 (2019):1820-1833.

Makino and Komiyama. Learning enhances the relative impact of top-down processing in the visual cortex. Nature neurosci. 18 (2015):1116-1122.

Ma et al. Divergent projections of the paraventricular nucleus of the thalamus mediate the selection of passive and active defensive behaviors. Nature neurosci. 24 (2021):1429-1440.

Peters et al. Emergence of reproducible spatiotemporal activity during motor learning. Nature 510 (2014):263-267.

Shang et al. Divergent midbrain circuits orchestrate escape and freezing responses to looming stimuli in mice. Nature communications 9 (2018):1232.

Shanks et al. Corticothalamic axons are essential for retinal ganglion cell axon targeting to the mouse dorsal lateral geniculate nucleus. J. of Neurosci. 36 (2016):5252-5263.

Wong and Brown. Visual detection, pattern discrimination and visual acuity in 14 strains of mice. Genes, brain, and behavior 5 (2006):389-403.

Yang et al. Plastic and stimulus-specific coding of salient events in the central amygdala. Nature 616 (2023):510-519.

Yilmaz and Meister. Rapid innate defensive responses of mice to looming visual stimuli. Current Biology 23 (2013):2011-2015.

Zhu et al. Dynamic salience processing in paraventricular thalamus gates associative learning. Science 362 (2018):423-429.

7. Please define in the main text what statistic is used to calculate the d prime for the task.

D prime is calculated as Z(Hit rate)-Z(FA rate), where Z(Hit rate) and Z(FA rate) are the Z-scores of Hit and FA rates, respectively. If FA rate was 0, FA rate of 1 over the total number of NO-GO trials was used to calculate Z(FA Rate) (**page 28, lines 16-18**). Following the reviewers' suggestion, we have added the formula of D prime in the main text (**page 4, line 3**).

8. The frequent use of the word "innate", clearly a 'loaded word' these days, is odd and vague: ''innate response" "innate circuit" "innate eye movements" "innate motor control" sounds undefined, or vague enough, that it is difficult to discern the intended meaning. Either be specific, or remove.

We agree with the reviewer's suggestion and have made the following changes. First, we replaced "innate eye movements/behaviour" with "reflexive eye movements/behaviour" to reflect the involuntary nature of the optokinetic reflex (**page 1, line 14; page 3, line 3; page 12, lines 14&26&28&29; page 13, line 12**). Second, we replaced "innate circuits" with "brainstem circuits" to specify the site of the circuits mediating reflexive behaviours (**page 1, line 19; page 3, line 9**). Last, when "innate" is unnecessary, we simply removed them (**page 5, line 32**).

9. The temporo-nasal selectivity of the background movement is tested on neural but not behavioral recordings. It is also unclear why said temporo-nasal direction selectivity of the NOT response would matter for saccadic suppression OKR or head motion, since all of these can occur in any direction, not only temporo-nasally. Please explain or remove these claims.

We thank the reviewer for the comments and suggestions. To address them, we performed a new behavioural experiment in which we presented temporo-nasal or naso-temporal background motion when mice performed the task of flash detection. We found that 1) both motion directions markedly impaired task performance and 2) silencing inhibitory NOT neurons recovered animals' performance only during temporo-nasal background motion, but not during naso-temporal background motion (**Fig. 6i-l**). This result is consistent with our findings in neural recording that 1) background gratings moving either temporo-nasally or naso-temporally can substantially suppress flash-evoked sSC activity and 2) silencing inhibitory NOT neurons alleviated the suppression of sSC activity specifically during temporo-nasal background motion (**Fig. 5j-m**). We have added the results from this experiment in the revised manuscript (**page 11, lines 28-31; page 12, lines 1-3; page 21, lines 12-14; page 33, lines 38,42-43**).

There are several important considerations regarding the interpretation of temporo-nasal bias in our findings. First, motion-induced suppression occurs not only during temporo-nasal background motion but also during other tested motion directions. Second, inhibitory NOT-sSC projections contribute specifically to motion-induced suppression during temporo-nasal motion, consistent with the temporo-nasal direction selectivity of this pathway. Third, the remaining suppression exerted by background motion after silencing this pathway does not have a temporo-nasal bias and may be mediated by circuits other than inhibitory NOT-sSC projections. We have revised the manuscript to reflect these points (**page 10, lines 11-13; page 12, lines 5-7**).

10. Lines 16-17 on page 10: please clarify that only the inhibitory NOT-SC neurons are silenced, rather than writing "we optogenetically silenced inhibitory NOT neurons".

In the behavioural experiment described on page 10, lines 16-17 of previous manuscript, we indeed silenced inhibitory NOT neurons using ArchT. This manipulation included inhibitory NOT-sSC projections, though not exclusively these projections. Nevertheless, we consider silencing inhibitory NOT-neurons to be functionally equivalent to silencing inhibitory NOT-sSC projections, because ArchT-based silencing of inhibitory NOT neurons restored flash-evoked sSC activity to a similar extent as eOPN3-based silencing of inhibitory NOT-sSC projections (**Fig. 5d**, **Extended Data Fig. 10h**). This finding indicates that inhibitory NOT neurons suppress sSC activity primarily through their direct projections to the sSC. Therefore, in the behavioural experiment involving optogenetics, we interpreted the effects of silencing inhibitory NOT neurons as the contribution of inhibitory NOT-sSC projections.

11. Line 6 page 11: please remove the generalization to human perception, where the retinal composition and projections are very different. Use the word 'may' if you refer to mechanisms in other mammals.

We agree with the reviewer's suggestion and have removed the generalization to humans and other mammals in the first paragraph of DISCUSSION (**page 12, line 19**).

12. Line 7 page 11: Which literature does the 'dichotomous view' under discussion comes from? In the mouse, the vast majority of the retinal projections go to the SC, which is the dominant structure for

visuo-motor integration.

The term 'dichotomous view' specifically refers to the division of visual systems involved in processing ***global motion***. To clarify this point, we have added the word "global" to the first sentence of this paragraph (**page 12, line 20**). Along the retina-dLGN-visual cortex pathway, motion-sensitive cortical areas MST/MT compute global motion by integrating local motion signals coming from V1 (*Smith et al., 2006; Fischer et al., 2012; Villeneuve et al., 2012; Wall et al., 2008*). And lesioning these higher visual areas compromises various motion perception tasks, including detection of global motion in the presence of directional noise and discrimination of the direction and speed of global motion (*Rudolph et al. 1999; Pasternak et al. 1994*). These findings support the view that this cortical pathway mediates global motion perception. In parallel, along the retina-accessory optic system (including the NOT) pathway, AOS neurons respond preferentially to global moving patterns (*Klauer et al., 1990; Hoffmann et al., 1975; Hoffmann et al., 1981; Ilg et al., 1996*). And silencing or lesioning AOS nuclei abolishes the optokinetic reflex (*Kato et al., 1986; Schiff et al., 1990; Hoffmann et al., 2001*), indicating that this pathway primarily mediates reflexive eye movements. Thus, a prevailing view in the field is that while the first pathway contributes mainly to motion-related perceptual phenomena, the latter pathway specializes in driving reflexive oculomotor control.

Although ~88% of mouse RGCs project to the SC (*Ellis et al. 2016*), a substantial proportion of RGCs also project to the dLGN, estimated at 25–50% in rodent/rabbit (*Dreher et al. 1985; Linden and Perry 1983; Martin 1986*). Notably, the dLGN shares a large portion of retinal inputs with the SC, as ~80% of dLGN-projecting RGCs also innervate the SC (*Ellis et al. 2016*). These findings indicate that the retina-dLGN-visual cortex pathway is equally important in visual processing in mice. Importantly, the motion-sensitive neurons in the SC respond predominantly to local, rather than global, motion (*Gale and Murphy 2016; De Franceschi and Solomon 2018; Wang et al. 2010;* and **Fig. 1k,m, Extended Data Fig. 3b,f,12e,h**) and therefore are not considered major contributors to global motion processing.

Nevertheless, we acknowledge that "the dichotomous view" is not a commonly used term to describe the functional specialization of global motion processing systems. We have therefore revised this sentence to prevent potential confusion (**page 12, line 20**).

References:

De Franceschi and Solomon. Visual response properties of neurons in the superficial layers of the superior colliculus of awake mouse. The J. of physiology 596 (2018):6307-6332.

Dreher et al. The morphology, number, distribution and central projections of class I retinal ganglion cells in albino and hooded rats. Brain Behav Evol (1985) 26:10–48.

Ellis et al. Shared and distinct retinal input to the mouse superior colliculus and dorsal lateral geniculate nucleus. J Neurophysiol. (2016) 116:602–610.

Fischer et al. Visual motion responses in the posterior cingulate sulcus: a comparison to V5/MT and MST. Cerebral Cortex 22 (2012):865-876.

Gale and Murphy. Active dendritic properties and local inhibitory input enable selectivity for object motion in mouse superior colliculus neurons. J. of Neurosci. 36 (2016):9111-9123.

Hoffmann et al. Directional effect of inactivation of the nucleus of the optic tract on optokinetic nystagmus in the cat. Vision research 41 (2001):3389-3398.

Hoffmann et al. Retinal input to direction selective cells in the nucleus tractus opticus of the cat. Brain Research 99 (1975):359-366.

Hoffmann et al. A quantitative analysis of the direction-specific response of neurons in the cat's nucleus of the optic tract. Experimental Brain Research 42 (1981):146-157.

Ilg et al. Responses of neurons of the nucleus of the optic tract and the dorsal terminal nucleus of the accessory optic tract in the awake monkey. Euro. J of Neurosci. 8 (1996):92-105.

Kato et al. Role of the nucleus of the optic tract in monkeys in relation to optokinetic nystagmus. Brain research 364 (1986):12-22.

Klauer et al. Visual response properties and afferents of nucleus of the optic tract in the ferret. Experimental Brain Research 83 (1990):178-189.

Linden & Perry. Massive retinotectal projection in rats. Brain Res (1983) 272:145–149.

Martin. The projection of different retinal ganglion cell classes to the dorsal lateral geniculate nucleus in the hooded rat. Exp Brain Res (1986) 62:77–88.

Pasternak et al. Motion perception following lesions of the superior temporal sulcus in the monkey. Cerebral Cortex 4 (1994):247-259.

Rudolph et al. Transient and permanent deficits in motion perception after lesions of cortical areas MT and MST in the macaque monkey. Cerebral Cortex 9 (1999):90-100.

Schiff et al. Effects of lesions of the nucleus of the optic tract on optokinetic nystagmus and after-nystagmus in the monkey. Experimental Brain Research 79 (1990):225-239.

Smith et al. Sensitivity to optic flow in human cortical areas MT and MST. Euro. J. of Neurosci. 23 (2006):561-569.

Villeneuve et al. Pattern-motion selective responses in MT, MST and the pulvinar of humans. Euro. J. of Neurosci. 36 (2012):2849-2858.

Wall et al. Selective visual responses to expansion and rotation in the human MT complex revealed by functional magnetic resonance imaging adaptation. Euro. J. of Neurosci. 27 (2008):2747-2757.

Wang et al. Visual receptive field properties of neurons in the superficial superior colliculus of the mouse. J. of Neurosci. 30 (2010):16573-16584.

13. Some figures have minor errors such as words cut off, or resizing issues.

We have carefully checked the figures in the revised manuscript to avoid words cut-off and resizing issues.

To all reviewers:

We would like to thank the reviewers for their helpful comments on the manuscript. We made revisions of the manuscript in the following aspects:

1. We revised the title to "An Inhibitory Brainstem Pathway Reduces Visual Detection During Background Motion".
2. We revised the sentence which defines background motion in the introduction (**page 2**, **line 4-5**).
3. We changed the unit of firing rate to spikes/s in the manuscript and figures.

Here are our replies to reviewers' comments.

Reviewer #1 (Remarks to the Author):

The authors have done a great job in addressing my comments and concerns. I am looking forward to seeing the manuscript in print.

We thank the reviewer again for the comments and suggestions, which certainly improved the quality of this study.

Reviewer #2 (Remarks to the Author):

In the revised submission, the authors have substantially improved the manuscript by providing new analyses and edits, and—admirably—new experiments. These additions meaningfully clarify: the rationale for the study, the structure and design of the task, the contrast dependence of the results, the relevance of the described pathway for gaze shifts and MOB.

We sincerely thank the reviewer for the positive assessment of the revised manuscript and the constructive suggestions below.

Most of our previous concerns have been carefully addressed. A few minor issues remain:

1. Conflation of surrounding (context) shifts and gaze shifts in the introduction. Lines 4–5: 'A prime example of this phenomenon is the perceptual effects of moving backgrounds, which naturally occur when the head, eyes or surroundings shift'. While surrounding shifts (e.g. looking our from a train) cause movement of the background and not the foreground, gaze shifts (e.g. saccades) cause global motion of the visual scene. Please rephrase this sentence to make clear that the type of motion involved is not the same in the two cases, and thus the perceptual mechanisms may differ.

The purpose of this sentence was to provide general examples of scenarios in which background motion occurs. We agree with the reviewer, however, that background motion resulting from gaze shifts differs from that caused by surrounding shifts. In response to this concern, we have removed references to head and eye shifts and now focus exclusively on background motion arising from surrounding shifts (**page 2**, **lines 4-5**). This decision was made because previous studies (**citations**

1-10), as well as our own experiments, have primarily used this form of background motion to demonstrate its modulatory effects on visual perception.

2. Consider rephrasing the title. The phrase "in motional context" is ambiguous— it could refer either to the observer motion or the visual scene motion. In addition, "impairs" may overstate the effect. A rephrasing such as "An inhibitory brainstem pathway reduces visual detection during background motion" may improve clarity, searchability, and overall impact.

Following the reviewer's suggestion, we changed the title to "An Inhibitory Brainstem Pathway Reduces Visual Detection During Background Motion".

3. Units for firing rate: firing rate should be reported in spikes/s rather than Hz. Please adjust throughout the manuscript.

We thank the reviewer for the suggestion and changed the units of firing rate to spikes/s throughout the manuscript (**page 5**, **line 4**; **page 6**, **lines 25&27**; **page 9**, **line 3&4**; **page 26**, **line 2**; **Fig. 1k**, **1l**, **2h**, **2i**, **3c**, **3e**, **3i**, **4b**, **4e**, **5b**, **5c**, **5g**, **5k**; **Supplementary Fig. 3b**, **3d**, **3e**, **6h**, **8d**, **9h**, **10b**, **10c**, **10f**, **10g**, **12b**, **12d**, **12e**, **12g**, **12h**).

4. Direction-specific suppression of SC neurons: the data clearly show that NOT suppression of SC responses is specific to temporo–nasal motion. If available (without requiring new experiments), it would be informative to examine whether this inhibition preferentially targets SC neurons tuned to the temporo–nasal direction as well.

We agree with the reviewer that it would be an interesting question for future investigation whether NOT-SC inhibition preferentially targets temporo-nasally biased SC neurons. However, the data from our experiments cannot address this question, because the background gratings used in this study primarily surrounded, rather than covered, the SC receptive fields and therefore evoked minimal responses in these neurons. As a result, this stimulation paradigm does not allow determination of the preferred direction of SC neurons. We did not present moving gratings of various directions covering the receptive fields of SC neurons, a stimulus configuration that is widely used to determine the direction preference of SC neurons.

Reviewer #3 (Remarks to the Author):

I co-reviewed this manuscript with one of the reviewers who provided the listed reports. This is part of the Nature Communications initiative to facilitate training in peer review and to provide appropriate recognition for Early Career Researchers who co-review manuscripts.

We sincerely thank the reviewer for providing constructive suggestions throughout the reviewing process.
